# Methyl-Thiol-Bridged Oxadiazole and Triazole Heterocycles as Inhibitors of NF-κB in Chronic Myelogenous Leukemia Cells

**DOI:** 10.3390/biomedicines11061662

**Published:** 2023-06-08

**Authors:** Basappa Basappa, Young Yun Jung, Akshay Ravish, Zhang Xi, Ananda Swamynayaka, Mahendra Madegowda, Vijay Pandey, Peter E. Lobie, Gautam Sethi, Kwang Seok Ahn

**Affiliations:** 1 Laboratory of Chemical Biology, Department of Studies in Organic Chemistry, University of Mysore, Mysore 570006, India; salundibasappa@gmail.com (B.B.); akshayrv533@gmail.com (A.R.); 2 Department of Science in Korean Medicine, Kyung Hee University, Seoul 02447, Republic of Korea; ve449@naver.com; 3 Shenzhen Bay Laboratory, Shenzhen 518055, China; zhangxi@szbl.ac.cn (Z.X.); pelobie@sz.tsinghua.edu.cn (P.E.L.); 4 Department of Studies in Physics, University of Mysore, Mysore 570006, India; anandas@physics.uni-mysore.ac.in (A.S.); mahendra@physics.uni-mysore.ac.in (M.M.); 5 Tsinghua Berkeley Shenzhen Institute, Tsinghua Shenzhen International Graduate School, Tsinghua University, Shenzhen 518055, China; vijay.pandey@sz.tsinghua.edu.cn; 6 Institute of Biopharmaceutical and Health Engineering, Tsinghua Shenzhen International Graduate School, Tsinghua University, Shenzhen 518055, China; 7 Department of Pharmacology, Yong Loo Lin School of Medicine, National University of Singapore, Singapore 117600, Singapore

**Keywords:** chronic myelogenous leukemia cells, NF-κB, triazole, oxadiazole, click chemistry

## Abstract

Nuclear factor kappa beta (NF-κB) is a transcriptional factor that plays a crucial role in regulating cancer cell proliferation. Therefore, the inhibition of NF-κB activity by small molecules may be beneficial in cancer therapy. In this report, methyl-thiol-bridged oxadiazole and triazole heterocycles were synthesized via click chemistry and it was observed that the lead structure, 2-(((1-(3,4-dichlorophenyl)-1H-1,2,3-triazol-4-yl)methyl)thio)-5-(4-methoxybenzyl)-1,3,4-oxadiazole (**4c**), reduced the viability of MCF-7 cells with an IC_50_ value of 7.4 µM. Compound **4c** also caused concentration-dependent loss of cell viability in chronic myelogenous leukemia (CML) cells. Furthermore, compound **4c** inhibited the activation of NF-κB in human CML cells as observed by nuclear translocation and DNA binding assays. Functionally, compound **4c** produced PARP cleavage and also suppressed expression of Bcl-2/xl, MMP-9, COX-2, survivin, as well as VEGF, resulting in apoptosis of CML cells. Moreover, ChIP assay showed that compound **4c** decreased the binding of *COX-2* to the p65 gene promoter. Detailed in silico analysis also indicated that compound **4c** targeted NF-κB in CML cells. In conclusion, a novel structure bearing both triazole and oxadiazole moieties has been identified that can target NF-κB in CML cells and may constitute a potential novel drug candidate.

## 1. Introduction

Chronic myelogenous leukemia (CML) primarily affects the blood and bone marrow [1]. According to the American Cancer Society, CML accounts for approximately 15% of all cases of leukemia in adults, and it is estimated that in the US, there is over 9000 new cases and more than 1000 deaths from the disease per year [2]. The most common drugs used to target CML cells are tyrosine kinase inhibitors such as imatinib, dasatinib, nilotinib, bosutinib, and ponatinib [3,4,5,6]. These drugs inhibit the BCR-ABL protein, which can also activate nuclear factor-kappa B (NF-κB) and promote cell proliferation, survival, and resistance to chemotherapy [7]. Inhibition of NF-κB has been reported to be a potential strategy for the treatment of CML [8]. NF-κB is a transcription factor implicated in regulation of a number of physiological and pathological processes [9,10]. NF-κB, a complex structure with subunits (p50, p52, RelA, RelB, and c-Rel), is activated by upstream activating kinases (IKKα, IKKβ, and IKKγ/NEMO) [11,12,13]. Tumor necrosis factor alpha (TNFα) is a pivotal cytokine that activates NF-κB [14,15,16,17]. When cells are exposed to TNFα, NF-κB is activated, which can lead to the production of diverse cellular responses [18]. Numerous disorders are also associated with aberrant activation of NF-κB in organs other than the immune system [19]. This may be attributed to the effect of NF-κB in controlling approximately 500 genes related to, cellular transformation, survival, proliferation, invasion, metastasis and angiogenesis [20,21,22]. Many extracellular signals, mainly through membrane-bound receptors and a series of adaptor and modulator proteins, activate NF-κB, producing nuclear translocation of NF-κB as a result of proteasome-mediated degradation of IκB [23]. The stimulation-induced phosphorylation of IκB by an IκB kinase (IKK) complex promotes proteasome-mediated degradation of IκB. There are two primary pathways involved in NF-κB activation: a classical (or canonical) pathway and an alternate (or non-canonical) pathway [24]. 

**Figure 1:**
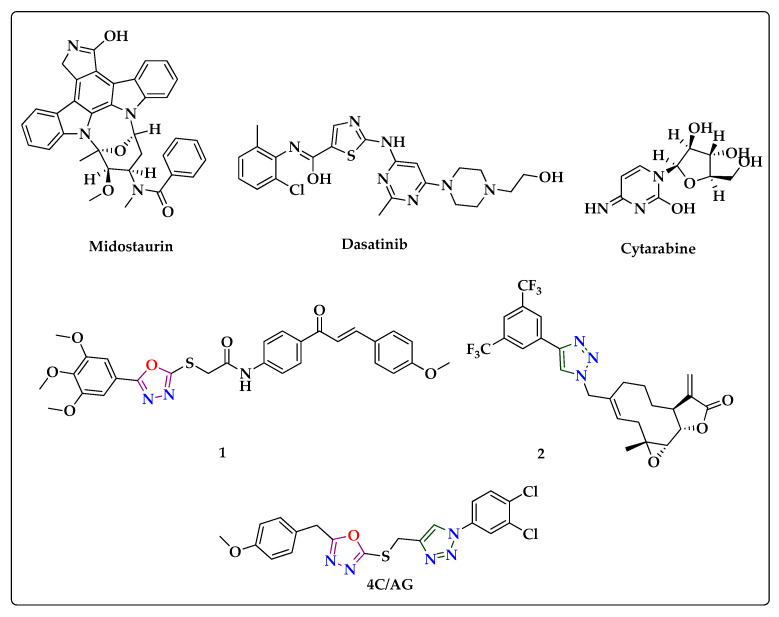
Structure of marketed drugs and reported lead molecules against leakemia (**1** & **2**) structures were shown.

Acute myelogenous, acute lymphoblastic, chronic myelogenous, chronic lymphocytic, and hairy cell leukemia cells are examples of leukemia cells that produce TNFα [25]. Indeed, the TNFα promoter has a variety of polymorphisms that have been linked to leukemia. The pathways involving NF-κB and c-Jun N-terminal kinase/activator protein-1 (JNK/AP-1) can mediate both anti-apoptotic and pro-proliferative actions of TNFα [26]. The IKK complex, which is made up of the catalytic subunits IKKα and IKKβ as well as a regulatory subunit IKKγ, is activated when TNFα binds to TNFR1 via the adaptor protein TNFα receptor-associated death domain (TRADD), with both TNF receptor-associated factor-2 (TRAF2) and receptor-interacting protein (RIP) [27]. When the IKK complex is activated, IκB is phosphorylated and degraded, which causes NF-κB to move into the nucleus [28]. Interestingly, higher levels of TNFα expression are frequently linked to unfavorable clinical characteristics and refractory disease in leukemia [29]. A number of chemists have synthesized small compounds (Figure 1) to inhibit leukemia by targeting TNFα [30]. Fathi et al. synthesized 1,3,4-oxadiazole-chalcone hybrids (**1**), which were reported to be effective against various leukemia variants with IC_50_ values between 1.73 and 2.05 µM [31]. Janganati et al. synthesized melampomagnolide B with 1,2,3-triazoles (**2**) and screened 60 human cancer cell lines to obtain a lead structure of IC_50_ of 400 nM, respectively, against AML cells [32]. We and others have previously explored the impact of novel heterocycles that can target NF-κB and other related oncogenic genes [33,34,35]. As NF-κB inhibitors such as sesquiterpene lactones and alkyl sulfides undergo nucleophilic aromatic substitution reactions with the amino acids of Cys38 of the NF-κB-p65 subunit, methyl-thiol-bridged oxadiazole/triazole-based have been designed as drug seeds to develop novel NF-κB targeting agents [35].

In the present work, novel compounds bearing 1,3,4-oxadiazole and 1,2,3-triazole motifs were synthesized and which are active in breast cancer and leukemia cells. Cell viability assays demonstrated that the newly synthesized compounds **4c**, **4b**, and **4a** possessed IC_50_ values of 7.40, 8.44, and 9.58 µM in MCF-7 cells. The lead compound **4c** was further studied for inhibition of NF-κB activation in CML cells.

## 2. Materials and Methods 

The various chemicals and solvents were obtained from Sigma-Aldrich, Bangalore, India. The completion of the reaction was monitored. ^1^H and ^13^C NMR were recorded on an Agilent (Santa Clara, CA, USA) NMR spectrophotometer (500 MHz), and CDCl_3_ was used as solvent.

### 2.1. General Procedure for the Synthesis of 1,2,3-Triazole Derivatives

Substituted phenylacetic hydrazides (**1**) were synthesized from phenylacetic acids. Further, hydrazides were treated with CS_2_ (1.2 mmol) under basic media for 8 h, the reaction was monitored by TLC, and after the completion of the reaction, neutralized by 30% HCl, the solid oxadiazole (**2**) formed was filtered and air dried [Calcd. for C_10_H_10_N_2_O_2_S^+^: 222.0463, Found = 223.1142 [M + 1]^+^. Oxadiazole (**2**) was reacted with propargyl bromide (1.2 mmol) and K_2_CO_3_ (1.5 mmol) for 1–2 h, and intermediate compound (**3**) was obtained and used to the next step without purification [Calcd. for C_13_H_12_N_2_O_2_S^+^: 260.0619, Found = 261.1316 [M + 1]^+^. Intermediate (**3)** (1.0 mmol), azide (1.2 mmol), CuI (0.2 mmol), and 3–5 drops of Et_3_N were stirred at room temperature in THF for 1–2 h. After the completion of the reaction, THF was distilled off and the crude was purified by column chromatography using ethyl acetate and hexane.

### 2.2. 2-(((1-(4-Chlorophenyl)-1H-1,2,3-triazol-4-yl)methyl)thio)-5-(4-methoxybenzyl)-1,3,4-oxadiazole (***4a***)

Yellow solid; mp: 92–94 °C; 87% yield; ^1^H NMR (500 MHz) CDCl_3_; δ 8.12 (s, 1H, Ar-H), 7.62 (d, J = 9.0 Hz, 2H, Ar-H), 7.47 (d, J = 9.0 Hz, 2H, Ar-H), 7.19 (d, J = 8.5 Hz, 2H, Ar-H), 6.84 (d, J = 8.5 Hz, 2H, Ar-H), 4.56 (s, 2H, -CH_2_), 4.08 (s, 2H, -CH_2_), 3.76 (s, 3H, -OCH_3_); ^13^C NMR (100 MHz) CDCl_3_; δ 167.18, 111.97, 111.45, 56.01, 164.30, 149.28, 148.65, 125.78, 121.78, 121.55, 144.05, 135.37, 134.76, 130.01, 121.18, 31.58, 26.68.

### 2.3. 2-(((1-(4-Bromophenyl)-1H-1,2,3-triazol-4-yl)methyl)thio)-5-(4-methoxybenzyl)-1,3,4-oxadia-zole (***4b***)

Yellow solid; mp: 86–88 °C; 89% yield; ^1^H NMR (500 MHz) CDCl_3_; δ 8.11 (s, 1H, Ar-H), 7.63 (d, J = 9.0 Hz, 2H, Ar-H), 7.56 (d, J = 9.0 Hz, 2H, Ar-H), 7.19 (d, J = 8.5 Hz, 2H, Ar-H), 6.84 (d, J = 8.5 Hz, 2H, Ar-H), 4.55 (s, 2H, -CH_2_), 4.08 (s, 2H, -CH_2_), 3.77 (s, 3H, -OCH_3_); ^13^C NMR (100 MHz) CDCl_3;_ δ 167.28, 135.86, 132.97, 130.05, 125.41, 164.26, 159.16, 144.13, 55.38, 31.14, 26.67, 122.62, 122.03, 121.50, 114.42; C_19_H_16_BrN_5_O_2_S: the calculated mass of the M + 2 ion is 458.3316, the resulting mass is 460.1086 [M + 2]^+^.

### 2.4. 2-(((1-(3,4-Dichlorophenyl)-1H-1,2,3-triazol-4-yl)methyl)thio)-5-(4-methoxybenzyl)-1,3,4-oxadiazole (***4c***)

Yellow solid; mp: 90–92 °C; 85% yield; ^1^H NMR (500 MHz) CDCl_3_; δ 8.13 (s, 1H, Ar-H), 7.85 (d, J = 2.0 Hz, 1H, Ar-H), 7.63–7.52 (m, 1H, Ar-H), 7.60–7.48 (m, 1H, Ar-H), 7.19 (d, J = 8.5 Hz, 2H, Ar-H), 6.84 (d, J = 9.0 Hz, 2H, Ar-H), 4.55 (s, 2H, -CH_2_), 4.08 (s, 2H, -CH_2_), 3.77 (s, 3H, -OCH_3_); ^13^C NMR (100 MHz) CDCl_3;_ δ 167.45, 164.61, 159.22, 143.40, 130.09, 129.34, 125.25, 122.53, 121.91, 55.41, 31.16, 26.76; C_19_H_15_Cl_2_N_5_O_2_S: the calculated mass of the M + 1 ion is 447.0324, the resulting mass is 448.0500.

### 2.5. 2-(4-Methoxybenzyl)-5-(((1-phenyl-1H-1,2,3-triazol-4-yl)methyl)thio)-1,3,4-oxadiazole (***4d***)

Brown solid; mp: 60–62 °C; 90% yield; ^1^H NMR (500 MHz) CDCl_3_; δ 8.12 (s, 1H, Ar-H), 7.66 (d, J = 8.0 Hz, 2H, Ar-H), 7.55–7.42 (m, 2H, Ar-H), 7.48–7.35 (m, 1H, Ar-H), 7.18 (d, J = 8.5 Hz, 2H, Ar-H), 6.83 (d, J = 9.0 Hz, 2H, Ar-H), 4.57 (s, 2H, -CH_2_), 4.08 (s, 2H, -CH_2_), 3.75 (s, 3H, -OCH_3_); ^13^C NMR (100 MHz) CDCl_3_; δ 167.22, 164.29, 159.13, 143.78, 136.91, 130.04, 129.82, 128.96, 125.45, 121.64, 120.67, 114.41, 55.37, 31.14, 26.78; C_19_H_17_N_5_O_2_S: the calculated mass of the M + 1 ion is 379.1103, the resulting mass is 380.1978.

### 2.6. 2-(((1-(3-Chlorophenyl)-1H-1,2,3-triazol-4-yl)methyl)thio)-5-(4-methoxybenzyl)-1,3,4-oxadiazole (***4e***)

Yellow solid; mp: 94–96 °C; 82% yield; ^1^H NMR (500 MHz) CDCl_3_; δ 8.13 (s, 1H, Ar-H), 7.31 (d, J = 2.0 Hz, 1H, Ar-H), 7.55–7.42 (m, 1H, Ar-H), 7.47–7.34 (m, 2H, Ar-H), 7.18 (d, J = 8.5 Hz, 2H, Ar-H), 6.83 (d, J = 8.5 Hz, 2H, Ar-H), 4.55 (s, 2H, -CH_2_), 4.08 (s, 2H, -CH_2_), 3.75 (s, 3H, -OCH_3_); ^13^C NMR (100 MHz) CDCl_3_; δ 167.27, 164.22, 159.15, 144.12, 137.72, 135.67, 130.89, 130.04, 129.02, 125.41, 121.60, 120.89, 118.59, 114.42, 55.37, 31.13, 26.66; C_19_H_16_ClN_5_O_2_S: the calculated mass of the M + 1 ion is 413.0713, the resulting mass is 414.1585.

### 2.7. 2-(4-Methoxybenzyl)-5-(((1-(4-methoxyphenyl)-1H-1,2,3-triazol-4-yl)methyl)thio)-1,3,4-oxadiazole (***4f***)

Orange solid; mp: 90–92 °C; 90% yield; ^1^H NMR (500 MHz) CDCl_3_; δ 8.03 (s, 1H, Ar-H), 7.56 (d, J = 9.0 Hz, 2H, Ar-H), 7.19 (d, J = 8.5 Hz, 2H, Ar-H), 6.98 (d, J = 9.0 Hz, 2H, Ar-H), 6.84 (d, J = 8.5 Hz, 2H, Ar-H), 4.56 (s, 2H, -CH_2_), 4.08 (s, 2H, -CH_2_), 3.85 (s, 3H, -OCH_3_), 3.76 (s, 3H, -OCH_3_); ^13^C NMR (100 MHz) CDCl_3_; 167.19, 164.34, 159.96, 159.13, 143.55, 130.36, 130.04, 125.47, 122.31, 121.75, 114.81, 114.42, 55.72, 55.37, 31.14, 26.83; C_20_H_19_N_5_O_3_S: the calculated mass of the M + 1 ion is 409.1209, the resulting mass is 410.2111.

### 2.8. 4-(4-(((5-(4-Methoxybenzyl)-1,3,4-oxadiazol-2-yl)thio)methyl)-1H-1,2,3-triazol-1-yl)phenol (***4g***)

Yellow solid; mp: 94–96 °C; 80% yield; ^1^H NMR (500 MHz) CDCl_3_; δ 7.99 (s, 1H, Ar-H), 7.46 (d, J = 9.0 Hz, 2H, Ar-H), 7.19 (d, J = 9.5 Hz, 2H, Ar-H), 6.93 (d, J = 9.0 Hz, 2H, Ar-H), 6.84 (d, J = 9.0 Hz, 2H, Ar-H), 4.55 (s, 2H, -CH_2_), 4.09 (s, 2H, -CH_2_), 3.76 (s, 3H, -OCH_3_); ^13^C NMR (100 MHz) CDCl_3_; δ 167.42, 164.58, 159.19, 156.88, 143.37, 130.06, 129.31, 125.22, 122.53, 121.88, 116.45, 114.46, 55.38, 31.13, 26.73; Calcd. for C_19_H_17_N_5_O_3_S: the calculated mass of the M + 1 ion is 395.1052, the resulting mass is 396.1937.

### 2.9. 2-(4-Methoxybenzyl)-5-(((1-(o-tolyl)-1H-1,2,3-triazol-4-yl)methyl)thio)-1,3,4-oxadiazole (***4h***)

Brown thick mass; 87% yield; ^1^H NMR (500 MHz) CDCl_3_; δ 7.84 (s, 1H, Ar-H), 7.38 (t, J = 7.3 Hz, 1H, Ar-H), 7.39–7.26–7.22 (m, 3H, Ar-H), 7.18 (d, J = 8.5 Hz, 2H, Ar-H), 6.82 (d, J = 8.5Hz, 2H, Ar-H), 4.57 (s, 2H, -CH_2_), 4.07 (s, 2H, -CH_2_), 3.75 (s, 3H, -OCH_3_), 2.14 (s, 3H, -CH_3_); ^13^C NMR (100 MHz) CDCl_3_; δ 167.19, 164.27, 159.13, 142.83, 136.32, 133.61, 131.57, 130.01, 126.92, 126.03, 125.48, 124.96, 114.41, 55.37, 31.12, 26.85, 17.95.

### 2.10. 2-(4-Methoxybenzyl)-5-(((1-(p-tolyl)-1H-1,2,3-triazol-4-yl)methyl)thio)-1,3,4-oxadiazole (***4i***)

Yellow solid; mp: 104–106 °C; 90% yield; ^1^H NMR (500 MHz) CDCl_3_; δ 7.84 (s, 1H, Ar-H), 7.38 (t, J = 7.3 Hz, 1H, Ar-H), 7.39–7.22 (m, 3H, Ar-H), 7.18 (d, J = 8.5 Hz, 2H, Ar-H), 6.82 (d, J = 8.5Hz, 2H, Ar-H), 4.57 (s, 2H, -CH_2_), 4.07 (s, 2H, -CH_2_), 3.75 (s, 3H, -OCH_3_), 2.14 (s, 3H, -CH_3_); ^13^C NMR (100 MHz) CDCl_3_; δ 167.19, 164.32, 159.12, 143.60, 139.09, 134.62, 130.30, 130.04, 125.46, 121.60, 120.57, 114.41, 55.36, 31.13, 26.81, 21.19; C_20_H_19_N_5_O_2_S: the calculated mass of the M + 1 ion is 393.1259, the resulting mass is 394.2163.

### 2.11. 2-(((1-(4-Chlorophenyl)-1H-1,2,3-triazol-4-yl)methyl)thio)-5-(3,4-dimethoxybenzyl)-1,3,4-oxadiazole (***4j***)

Yellow solid; mp: 72–74 °C; 85% yield; ^1^H NMR (500 MHz) CDCl_3_; δ 8.12 (s, 1H, Ar-H), 7.63 (d, J = 8.5 Hz, 2H, Ar-H), 7.47 (d, J = 9.0 Hz, 2H, Ar-H), 6.81 (d, J = 2.0 Hz, 1H, Ar-H), 6.80 (s, 1H, Ar-H), 6.78 (d, J = 2.0 Hz, 1H, Ar-H), 4.56 (s, 3H, -CH_2_), 4.08 (s, 3H, -CH_2_), 3.84 (s, 3H, -OCH_3_), 3.84 (s, 3H, -OCH_3_); ^13^C NMR (100 MHz) CDCl_3_; δ 167.18, 164.30, 149.28, 148.65, 144.05, 135.37, 134.76, 130.01, 125.78, 121.78, 121.55, 121.18, 111.97, 111.45, 56.01, 31.58, 26.68; C_20_H_18_ClN_5_O_3_S: the calculated mass of the M + 1 ion is 443.0819, the resulting mass is 444.1035.

### 2.12. 2-(((1-(4-Bromophenyl)-1H-1,2,3-triazol-4-yl)methyl)thio)-5-(3,4-dimethoxybenzyl)-1,3,4-oxadiazole (***4k***)

Yellow solid; mp: 98–100 °C; 83% yield; ^1^H NMR (500 MHz) CDCl_3_; δ 8.12 (s, 1H, Ar-H), 7.63 (d, J = 9.0 Hz, 2H, Ar-H), 7.56 (d, J = 9.0 Hz, 2H, Ar-H), 6.81 (d, J = 2.0 Hz, 1H, Ar-H), 6.80 (s, 1H, Ar-H), 6.78 (d, J = 2.0 Hz, 1H, Ar-H), 4.56 (s, 3H, -CH_2_), 4.08 (s, 3H, -CH_2_), 3.84 (s, 3H, -OCH_3_), 3.84 (s, 3H, -OCH_3_); ^13^C NMR (100 MHz) CDCl_3_; δ 167.18, 164.29, 149.28, 148.65, 144.08, 135.85, 132.98, 125.78, 122.64, 122.00, 121.48, 121.18, 111.97, 111.45, 56.01, 31.58, 26.68; C_20_H_18_BrN_5_O_3_S: the calculated mass of the M + 2 ion is 488.3576, the resulting mass is 490.0195.

### 2.13. 2-(((1-(3,4-Dichlorophenyl)-1H-1,2,3-triazol-4-yl)methyl)thio)-5-(3,4-dimethoxybenzyl)-1,3,4-oxadiazole (***4l***)

Yellow solid; mp: 102–104 °C; 87% yield; ^1^H NMR (500 MHz) CDCl_3_; δ 8.14 (s, 1H, Ar-H), 7.85 (d, J = 2.5 Hz, 1H, Ar-H), 7.57 (s, 1H, Ar-H), 7.55 (d, J = 2.0 Hz, 1H, Ar-H), 6.81 (d, J = 2.0 Hz, 1H, Ar-H), 6.80 (s, 1H, Ar-H), 6.78 (d, J = 2.0 Hz, 1H, Ar-H), 4.56 (s, 2H, -CH_2_), 4.08 (s, 2H, -CH_2_), 3.84 (s, 3H, -OCH_3_), 3.84 (s, 3H, -OCH_3_); ^13^C NMR (100 MHz) CDCl_3_; δ 167.23, 164.26, 149.28, 148.66, 144.33, 135.88, 134.10, 133.10, 131.54, 125.75, 122.35, 121.53, 121.19, 119.51, 111.98, 111.45, 56.00, 31.58, 26.59; C_20_H_17_Cl_2_N_5_O_3_S: the calculated mass of the M + 1 ion is 477.0429, the resulting mass is 478.1408.

### 2.14. 2-(3,4-Dimethoxybenzyl)-5-(((1-phenyl-1H-1,2,3-triazol-4-yl)methyl)thio)-1,3,4-oxadiazole (***4m***)

Brown solid; mp: 46–48 °C; 90% yield; ^1^H NMR (500 MHz) CDCl_3_; δ 8.13 (s, 1H, Ar-H), 7.67 (d, J = 8.0 Hz, 2H, Ar-H), 7.56–7.43 (m, 2H, Ar-H), 7.48–7.35 (m, 1H, Ar-H), 6.81 (d, J = 2.0 Hz, 1H, Ar-H), 6.80 (s, 1H, Ar-H), 6.78 (d, J = 1.5 Hz, 1H, Ar-H), 4.58 (s, 2H, -CH_2_), 4.08 (s, 2H, -CH_2_), 3.83 (d, J = 1.3 Hz, 6H, -OCH_3,_ -OCH_3_); ^13^C NMR (100 MHz) CDCl_3_; δ 167.13, 164.34, 149.27, 148.62, 143.74, 136.90, 129.83, 128.98, 125.82, 121.62, 121.17, 120.65, 111.95, 111.45, 56.00, 31.58, 26.79; C_20_H_19_N_5_O_3_S: the calculated mass of the M + 1 ion is 409.1209, the resulting mass is 410.2111.

### 2.15. 2-(((1-(3-Chlorophenyl)-1H-1,2,3-triazol-4-yl)methyl)thio)-5-(3,4-dimethoxybenzyl)-1,3,4-oxadiazole (***4n***)

Yellow solid; mp: 70–72 °C; 87% yield; ^1^H NMR (500 MHz) CDCl_3_; δ 8.14 (s, 1H, Ar-H), 7.79–7.69 (m, 1H, Ar-H), 7.53–7.51 (m, 1H, Ar-H), 7.49–7.36 (m, 1H, Ar-H), 7.45–7.34 (m, 1H, Ar-H), 6.86–6.74 (m, 1H, Ar-H), 6.78 (d, J = 1.5 Hz, 1H, Ar-H), 4.56 (s, 2H, -CH_2_), 4.08 (s, 2H, -CH_2_), 3.83 (s, 3H, -OCH_3_), 3.83 (s, 3H, -OCH_3_); ^13^C NMR (100 MHz) CDCl_3_; δ 167.19, 164.27, 149.27, 148.64, 144.08, 137.71, 135.68, 130.91, 129.03, 125.78, 121.59, 121.18, 120.87, 118.57, 111.97, 111.45, 56.00, 31.58, 26.66; C_20_H_18_ClN_5_O_3_S: the calculated mass of the M + 1 ion is 443.0819, the resulting mass is 444.1768.

### 2.16. 2-(3,4-Dimethoxybenzyl)-5-(((1-(4-methoxyphenyl)-1H-1,2,3-triazol-4-yl)methyl)thio)-1,3,4-oxadiazole (***4o***)

Yellow solid; mp: 76–78 °C; 88% yield; ^1^H NMR (500 MHz) CDCl_3_; δ 8.03 (s, 1H, Ar-H), 7.56 (d, J = 9.0 Hz, 2H, Ar-H), 6.98 (d, J = 9.0 Hz, 2H, Ar-H), 6.81 (d, J = 2.0 Hz, 1H, Ar-H), 6.80 (s, 1H, Ar-H), 6.83–6.73 (m, 1H, Ar-H), 4.56 (s, 2H, -CH_2_), 4.08 (s, 2H, -CH_2_), 3.84 (s, 3H, -OCH_3_), 3.84 (s, 3H, -OCH_3_), 3.83 (s, 3H, -OCH_3_); ^13^C NMR (100 MHz) CDCl_3_; δ 167.11, 164.38, 159.97, 149.27, 148.62, 143.50, 130.34, 125.83, 122.28, 121.73, 121.17, 114.82, 111.95, 111.45, 56.00, 55.72, 31.58, 26.83; C_21_H_21_N_5_O_4_S: the calculated mass of the M + 1 ion is 439.1314, the resulting mass is 440.2345.

### 2.17. 2-(4-(((5-(3,4-Dimethoxybenzyl)-1,3,4-oxadiazol-2-yl)thio)methyl)-1H-1,2,3-triazol-1-yl)phenol (***4p***)

Yellow solid; mp: 122–124 °C; 87% yield; ^1^H NMR (500 MHz) CDCl_3_; δ 8.00 (s, 1H, Ar-H), 7.47 (d, J = 8.5 Hz, 2H, Ar-H), 6.93 (d, J = 9.0 Hz, 2H, Ar-H), 6.81 (d, J = 2.0 Hz, 1H, Ar-H), 6.80 (s, 1H, Ar-H), 6.78 (d, J = 1.2 Hz, 1H, Ar-H), 4.56 (s, 2H, -CH_2_), 4.09 (s, 2H, -CH_2_), 3.83 (s, 3H, -OCH_3_), 3.83 (s, 3H, -OCH_3_); ^13^C NMR (100 MHz) CDCl_3_; δ 167.27, 156.69, 149.08, 143.45, 137.97, 134.65, 130.18, 125.67, 122.51, 121.82, 121.20, 116.42, 111.96, 111.47, 56.00, 31.57, 26.77; C_20_H_19_N_5_O_4_S: the calculated mass of the M + 1 ion is 425.1158, the resulting mass is 426.2071.

### 2.18. 2-(3,4-Dimethoxybenzyl)-5-(((1-(p-tolyl)-1H-1,2,3-triazol-4-yl)methyl)thio)-1,3,4-oxadia-zole (***4q***)

Yellow solid; mp: 92–94 °C; 87% yield; ^1^H NMR (500 MHz) CDCl_3_; δ 8.08 (s, 1H, Ar-H), 7.53 (d, J = 8.0 Hz, 2H, Ar-H), 7.27 (d, J = 8.5 Hz, 2H, Ar-H), 6.81 (d, J = 1.5 Hz, 1H, Ar-H), 6.79 (s, 1H, Ar-H), 6.78 (d, J = 1.5 Hz, 1H, Ar-H), 4.57 (s, 2H, -CH_2_), 4.08 (s, 2H, -CH_2_), 3.83 (s, 6H, -OCH_3_, -OCH_3_), 2.40 (s, 3H, -CH_3_); ^13^C NMR (100 MHz) CDCl_3_; δ 167.10, 164.36, 149.27, 148.61, 143.56, 139.11, 134.62, 130.31, 125.84, 121.57, 121.17, 120.54, 111.95, 111.44, 55.99, 31.58, 26.83, 21.19.

### 2.19. 2-(3,4-Dimethoxybenzyl)-5-(((1-(o-tolyl)-1H-1,2,3-triazol-4-yl)methyl)thio)-1,3,4-oxadia-zole (***4r***)

Brown thick mass; 65% yield;^1^H NMR (500 MHz) CDCl_3_; δ 7.86 (s, 1H, Ar-H), 7.45–7.32 (m, 1H, Ar-H), 7.39–7.26 (m, 1H, Ar-H), 7.34–7.21 (m, 1H, Ar-H), 6.80 (s, 1H, Ar-H), 6.84–6.72 (m, 1H, Ar-H), 4.59 (s, 2H, -CH_2_), 4.08 (s, 2H, -CH_2_), 3.83 (s, 2H, -OCH_3_), 3.83 (s, 3H, -OCH_3_), 2.15 (s, 3H, -CH_3_); C_21_H_21_N_5_O_3_S: C_20_H_19_N_5_O_4_S: the calculated mass of the M + 1 ion is 423.1365, the resulting mass is 424.2278.

### 2.20. Reagents

Tris-base, Gly, NaCl, SDS, MTT, and BSA were purchased from Sigma-Aldrich (St. Louis, MO, USA). The LightShift^®^ Chemiluminescent EMSA Kit was purchased from Thermo Fisher Scientific Inc. (Waltham, MA, USA). Anti-phospho-IKKα/β, anti-IKKβ, anti-IκBα anti-IKKα, anti-phospho-IκBα antibodies were purchased from Cell Signaling Technology (Beverly, MA, USA). Anti-phospho-p65, anti-p65, anti-survivin, anti-Bcl-2, anti-Bcl-xl, anti-*COX-2*, anti-VEGF, anti-MMP-9, anti-PARP, anti-Lamin B, and anti-β-actin antibodies were purchased from Santa Cruz Biotechnology (Santa Cruz, CA, USA). Alexa Fluor^®^ 594 donkey anti-rabbit IgG (H + L) antibody was obtained from Life Technologies (Grand Island, NY, USA). The FITC Annexin V Apoptosis Detection Kit I was purchased from (BD Biosciences, Becton-Dickinson, Franklin Lakes, NJ, USA).

### 2.21. Cell Lines and Culture Conditions

MCF-7 cells were purchased from Procell Life Science and Technology Co., Ltd., Wuhan, China, and cultured in MEM or Leibovitz’s L-15 medium containing 2% FBS. MCF-7 cells were maintained in a 5% CO_2_-humidified atmosphere at 37 °C. KCL22, LAMA84, and K562 cells were obtained from the American Type Culture Collection (Manassas, VA, USA). The KBM5 cell line was previously provided by Dr. Bharat Aggarwal (University of Texas M.D. Anderson Cancer Center, Houston, TX, USA). KBM5 cells were cultured in IMDM medium with 10% inactivated FBS and 1% penicillin-streptomycin. KCL22, LAMA84, and K562 cells were cultured in RPMI with 10% FBS and 1% penicillin-streptomycin antibiotics and maintained at 37 °C in a 5% CO_2_ chamber.

### 2.22. MTT Assay

Cells (1 × 10^4^ cells/well) were seeded on a 96-well plate and treated with **4c** (0, 5, 10, 15, 30, 50 µM) for 24 h. After 24 h of treatment, 30 µL of MTT solution (2 mg/mL) for 2 h, followed by MTT lysis overnight. Cell viability was analyzed by measuring the absorbance of lysed MTT crystals using VARIOSKAN LUX (Thermo Fisher Scientific Inc., Waltham, MA, USA) at 570 nm.

### 2.23. Electrophoretic Mobility Shift Assay (EMSA)

CML cells (1 × 10^6^ cells/well) were treated with indicated concentrations of **4c** (0, 5, 15, 30 µM) for 2 h, then TNFα (0.5 nM) for 10 min. Nuclei extracts were prepared from whole cell pellets and incubated with NF-κB oligonucleotides (5′-AGTTGAGGGGACTTTCCCAGGC-3′ and 5′-GCCTGGAAAGTCCCCTCAACT-3′), and Oct-1 (5′-TTCTAGTGATTTGCATTCGACA-3′ and 5′-TGTCGAATGCAAA -TCACTAGAA-3′) was used for loading control. The protein–DNA complex was run on a 5% gel, and the respective proteins were detected using the EMSA kit (Waltham, MA, USA).

### 2.24. Immunocytochemistry

CML cells (1 × 10^6^ cells/well) were treated with **4c** (0 or 30 µM) for 2 h, followed by TNFα (0.5 nM) addition for 10 min. Further, the CML cells were fixed, permeabilized, and blocked with 5% BSA-PBS. The cells were visualized by using primary anti-p65 antibodies and secondary antibodies called Alexa Fluor^®^ 594 donkey anti-rabbit IgG (H + L). Nuclei were stained with DAPI, and the immobilized cells were mounted with Fluorescent Mounting Medium (Golden Bridge International Labs, Mukilteo, WA, USA) and finally quantified using an Olympus FluoView FV1000 confocal microscope (Tokyo, Japan).

### 2.25. Western Blot Analysis

CML cells were treated with compound **4c**, harvested, and the cell lysates were obtained. The required proteins of our interest were separated on SDS-PAGE and transferred into the nitrocellulose membrane. The membrane was incubated with primary antibody and HRP-conjugated secondary antibody in a sequential manner. The ECL kit (EZ-Western Lumi Femto, DOZEN) was used to detect the respective proteins.

### 2.26. Live and Dead Assay

CML cells (1 × 10^6^ cells/well) were incubated with compound **4c** (0 or 30 µM) for 24 h, and then TNFα (0.5 nM) was used as an inducer of NF-κB for 10 min. Thereafter, the cells were stained with Calcein AM and Ethd-1 (ethidium homodimer-1) reagents and detected using an Olympus FluoView FV1000 confocal microscope (Tokyo, Japan).

### 2.27. Annexin V Assay

CML cells (1 × 10^6^ cells/well) were incubated with compound **4c** (0 or 30 µM) for 24 h and then treated with TNFα (0.5 nM) for 10 min. The washed cells were tagged with FITC-bound annexin V antibodies and stained with PI for 15 min at room temperature. Finally, CML cells were analyzed by the BD Accuri™ C6 Plus flow cytometer (BD Biosciences, Becton-Dickinson, Franklin Lakes, NJ, USA) and analyzed with BD Accuri C6 Plus software.

### 2.28. Cell Cycle Analysis

CML cells (1 × 10^6^ cells/well) were incubated with compound **4c** (0 or 30 µM) for 24 h, then treated with TNFα (0.5 nM) for 10 min, washed, fixed, and finally incubated with 1 mg/mL RNase A for 1 h at 37 °C. After staining with PI, the CML cells were analyzed by the BD Accuri™ C6 Plus flow cytometer (BD Biosciences, Becton-Dickinson, Franklin Lakes, NJ, USA) and quantified using BD Accuri C6 Plus software.

### 2.29. Chromatin Immunoprecipitation (ChIP) Assay

CML cells (1 × 10^6^ cells/well) were incubated with compound **4c** (5, 15, 30 µM) for 24 h and immunoprecipitated with anti-NF-κB p65 antibody. RT-PCR was performed with *COX-2* at 94 °C for 5 min, 94 °C for 30 s, 58 °C for 30 s, 72 °C for 30 s with 30 cycles, and extension at 72 for 7 min. All the experiments were performed 3 times as individual repeats.

### 2.30. In Silico DFT Calculations

Computational DFT studies for the molecules were performed using Gaussian09 [35]. The molecule was built and visualized by the Gaussview 5 program package. The molecular geometry of the molecules was optimized at the B3LYP level with a 6-311++G (d,p) basis set [36].

### 2.31. Molecular Docking and Dynamics Analysis

The molecular docking simulation was performed with Autodock 4.2 [37] to analyze the best binding pose and the binding affinity of **4c** in the binding pocket of the P65 subunit of NF-κB. The crystal structure of the IκBalpha/NF-κB complex was obtained from the RCSB Protein Data Bank, having PDB ID: 1IKN (https://www.rcsb.org/) (accessed on 23 January 2023). Initially, to begin with, the receptor was prepared to dock by removing the heteroatoms and water molecules present in it. Followed by the addition of polar hydrogens and Kollman charges. The docking model was built with a grid box size of cell dimensions of 126 × 126 × 126 Å with 0.219 Å spacing. The empirical free-energy function and the Lamarckian genetic algorithm were used to perform molecular docking with the macromolecule. The initial population consisted of 150 randomly assigned individuals, a maximum of 2,500,000 energy evaluations, a mutation rate of 0.02, a crossover rate of 0.80, and 30 docking runs. The binding energy value of the ligand and the contact bonds with NF-κB were investigated. The docking interactions were visualized and studied using Maestro and PyMOL (v2.5.2) [38,39,40].

Desmond, the Schrödinger program [41], was used for MD calculations. The chosen ligand–protein combinations were first submerged in a TIP3P water box 10 Å beyond any of the complex’s atoms. Counter ions (Na^+^ and Cl^−^ ions) were introduced to balance charges. The salt content was adjusted to 0.15 M sodium and chloride ions to simulate physiological conditions. The MD was carried out in the NPT ensemble over 50 ns at 300 K and 1.63 bar pressure, with recording periods of 1.2 ps for energy and 100 ps for trajectory. The OPLS-3e force field was used in the simulations.

### 2.32. Statistical Analysis

All the numerical values have been represented as the mean ± SD. Statistical significance of the data compared with the untreated control was determined using the Student’s unpaired *t*-test. Significance was set at * *p* < 0.05, ** *p* < 0.01, and *** *p* < 0.001.

## 3. Results

### 3.1. Method of Synthesis

**Scheme 1:**
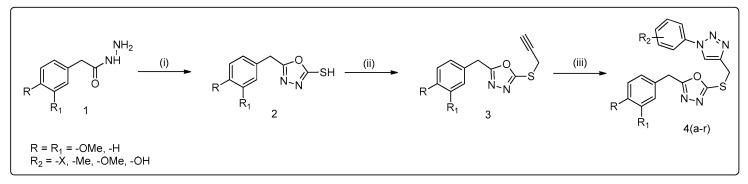
Reaction condition: (i) CS_2_, KOH, EtOH, reflux, 8 h; (ii) propargyl bromide, K_2_CO_3_, acetone reflux, 1–2 h; (iii) azide, CuI, THF, rt, 1–2 h.

We have previously synthesized various types of oxadiazoles and triazoles and reported their biological properties [42,43]. Herein, we have synthesized methoxy derivatives of 1-phenyl-1H-1,2,3-triazol-4yl)methyl)thio)-1,3,4-oxadiazoles using 4-methoxyphenyl-acetic acid and 3,4-dimethoxyphenylacetic acid to form their respective esters by refluxing with ethanol and a catalytic amount of sulfuric acid; further treating with hydrazine hydrate yields respective hydrazides (**1**). Later, in the presence of ethanolic KOH, cyclizing with carbon disulfide yields oxadiazole (**2**), which is then reacted with propargyl bromide in acetone under basic conditions to yield compound (**3**). Then, by using “click chemistry,” 4-methoxyphenyl and 3,4-dimethoxyphenyl-oxadiazole-triazole derivatives (**4a–r**) were synthesized by 1,3-dipolar cycloaddition with various substituted azides, generally termed click chemistry (Scheme 1). Using spectroscopic methods, we characterized the title compounds (Table 1).

### 3.2. Efficacy of Title Compounds in Breast Cancer Cells

A number of studies have shown that the NF-κB pathway is crucial for the growth and progression of breast cancer, and thereby it serves as a potential target for breast cancer prevention and treatment [44]. We therefore screened all title compounds for loss of viability of MCF-7 cells. The results summarized in Table 1 show that the title compounds significantly inhibited the viability of MCF-7 cells. Compounds bearing 4-methoxyphenyl derivatives such as **4a**, **4b**, **4c**, and **4e** produced significant loss of viability in MCF-7 cells with IC_50_ values of 9.58, 8.44, 7.40, and 16.86 µM, respectively. Furthermore, the most active compounds, such as **4a**, **4b**, **4c**, **4e**, and **4q**, exhibited IC_50_ values in MCF-10A cells greater than 100 µM, thus indicating a minimal effect on normal mammary epithelial cells (please refer to Supplementary Data S1). Compound **4c** was observed to be the most effective among the tested title compounds. Among the newly synthesized oxadiazole and triazole compounds, compounds bearing a 4-methoxyphenyl group were observed to be more active than those bearing a 3,4-dimethoxyphenyl group. Furthermore, triazole compounds bearing halogen (**4a**, **4b**, **4c**/AG, and **4e**) showed the lowest IC_50_ values of 9 to 16 µM. Hence, it was assumed that 4-methoxy and halides substituted for oxadiazole and triazole, respectively, are the most active among methyl-thiol-bridged oxadiazole and triazole molecules. 

### 3.3. ***4c*** Suppressed the Viability of CML Cells

**Figure 2:**
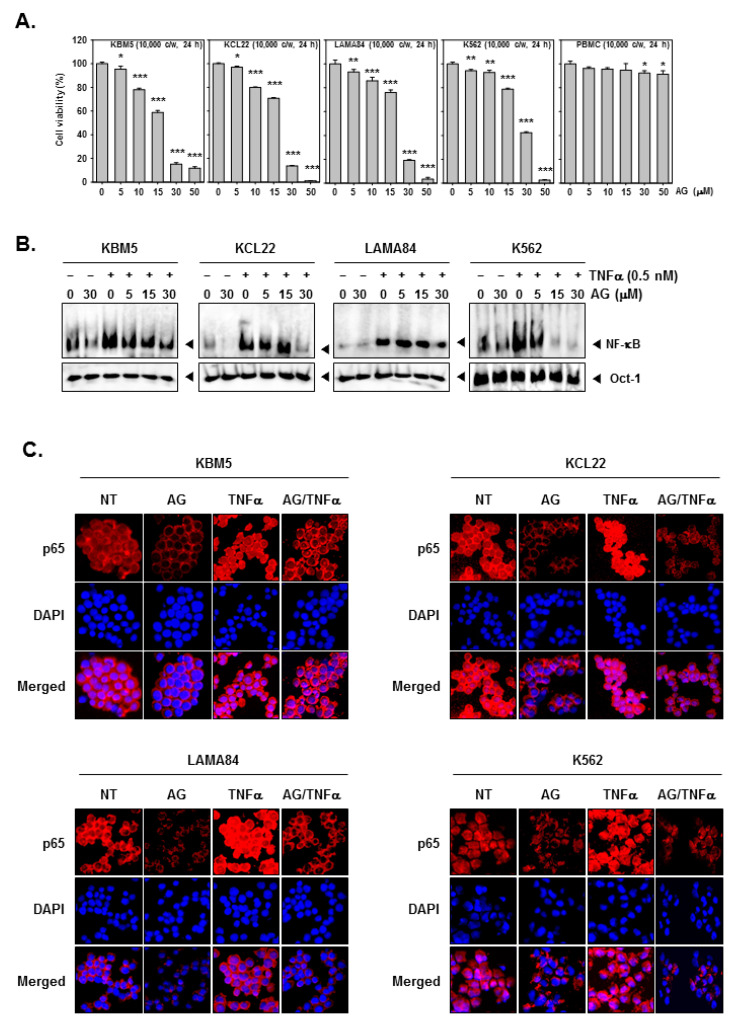
Inhibitory effects of **4c** on NF-κB activation in CML cells. (**A**) Cell viability of KMB5, KCL22, LAMA84, K562, and PBMC was measured by MTT assay. (**B**) Nuclear extracts were prepared for EMSA assay to evaluate NF-κB. (**C**) Cells were treated as described above. Immunocytochemistry was done to analyze p65 translocation into the nucleus. *** *p* < 0.001 vs. non-treated (NT) cells; ** *p* < 0.01 vs. non-treated (NT) cells; * *p* < 0.05 vs. non-treated (NT) cells. The results shown are representative of at least three independent experiments.

Preclinical studies of IT-901, a novel and selective NF-κB inhibitor, revealed that it significantly reduced the tumor burden in a xenograft model that was implanted with chronic lymphocytic leukemia patient-derived cells [45]. Therefore, we tested the most active compound **4c** against leukemia cell viability (KMB5, KCL22, LAMA84, and K562 cell lines) using a cell viability assay. Compound **4c** was applied to cells in a dose-dependent manner (0, 5, 10, 15, 30, and 50 µM) for 24 h. Compound **4c** was observed to produce loss of cell viability in CML cells (30 µM). However, cell viability in the normal PBMC cells was maintained at greater than 80% even at drug concentrations of 30 and 50 µM (Figure 2A). These results encouraged us to understand the mode of action of compound **4c**.

### 3.4. Compound ***4c*** Inhibited the TNFα Induced NF-κB Activation in CML Cells

TNFα, acting as a proinflammatory cytokine, can activate NF-κB, which leads to the transcriptional regulation of genes that are involved in cancer cell proliferation. An EMSA assay was used to detect the binding of NF-κB to its respective DNA binding sites. The inhibitory effects of **4c** on TNFα-induced NF-κB activation in CML cells were therefore investigated. For this purpose, CML cells were incubated with **4c** (0, 5, 15, 30 µM) for 2 h, followed by TNFα (0.5 nM) incubation for 10 min. After the reaction, nuclear extracts of CML cells were used to equally quantify NF-κB oligonucleotides that bound to DNA. It was observed that in all the tested CML cells, the binding of NF-κB to DNA was induced by TNFα, whereas the binding pattern was substantially inhibited by compound **4c** (Figure 2B).

Furthermore, immunocytochemistry (ICC) was used to determine the effect of **4c** on the translocation of NF-κB-p65 in CML cells. CML cells were grown on coverslips and treated with compound **4c** (0 or 30 µM) for 2 h, followed by TNFα (0.5 nM) for 10 min. Treatment with compound **4c** inhibited translocation of NF-κB-p65 in CML cells compared with non-treated (NT) cells; NF-B-p65 antibodies stained the nucleus more strongly in cells treated with TNFα alone. Despite TNFα stimulation, **4c** suppressed the translocation of NF-κB-p65 to nuclei (Figure 2C). The results of this study revealed that compound **4c** inhibited NF-κB activation in human CML cells.

### 3.5. Compound ***4c*** Inhibited NF-κB Complex Formation in CML Cells

**Figure 3:**
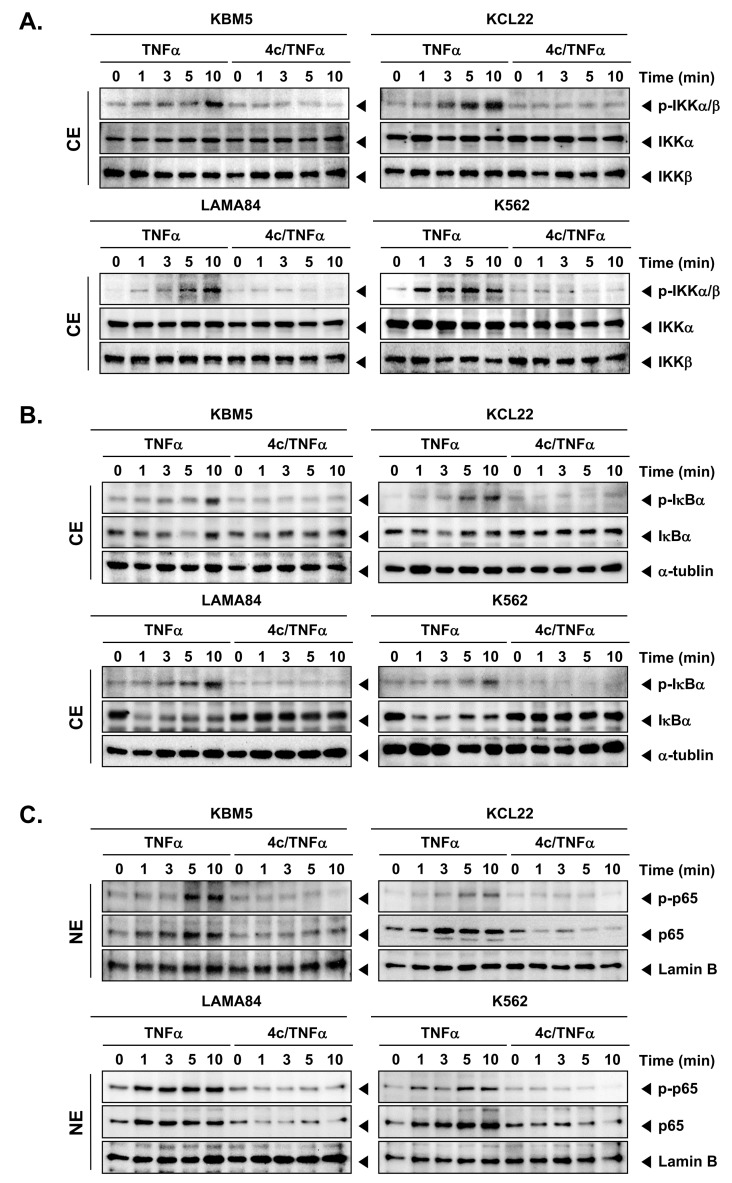
Modulation of IκBα degradation by **4c**. (**A**) Cytoplasmic extracts were prepared to evaluate the expression of phospho-IKKα/β, IKKα, and IKKβ by Western blot analysis. (**B**) Cytoplasmic and nuclear extracts were analyzed to evaluate NF-κB activation and IκBα degradation. (**C**) p65 expression was determined by Western blot analysis using nuclear extracts. The results shown are representative of at least three independent experiments.

CML cells were stimulated by TNFα, which binds to the TNF receptor via several intermediate steps to engage the IκB kinase (IKK) complex, which then phosphorylates IκB, and ultimately causes IκB degradation. The heterodimer (consisting of p65 and p50 subunits) then translocate into the nucleus and bind to the target DNA sequences. Since NF-κB targeting drugs inhibit the activation of the NF-κB complex, a Western blot analysis was performed. Therefore, CML cells were pre-treated compound **4c** (0 or 30 µM), followed by the incubation with TNFα (0.5 nM) at various time points. The cytosol and nuclear extracts of CML cells were prepared and the expression of protein levels was analyzed by Western blot. It was confirmed that compound **4c** inhibited NF-κB complex-related subunits such as p-IKKα/β and p-ΙκΒα, which were induced by TNFα in the cytoplasmic region of CML cells, when compared to **4c**-untreated cells (Figure 3A,B). Furthermore, p65 activation by TNFα was determined, wherein **4c** suppressed phospho-p65 in nuclei (Figure 3C). In summary, compound **4c** targets NF-κB activation in CML cells by diverse mechanisms.

### 3.6. Compound ***4c*** Inhibits Binding of COX-2 to p65 Promoter in CML Cells

**Figure 4:**
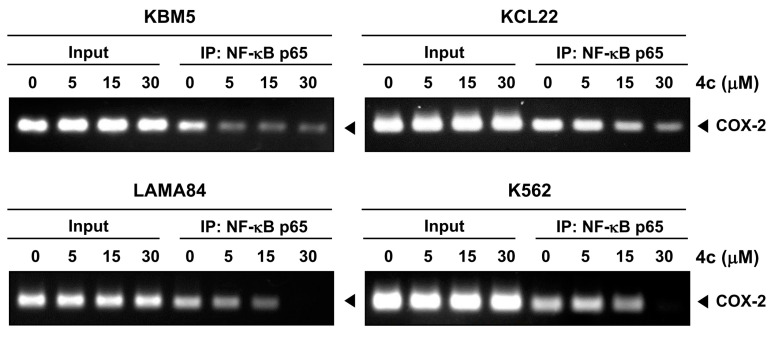
Inhibition effects of **4c** on *COX-2* binding to p65 promoter. The results shown are representative of at least three independent experiments.

It is important to note that the EMSA method only detects the binding of NF-κB to DNA and does not directly measure the downstream effects of NF-κB activation. Therefore, ChIP assays were performed to confirm the inhibition of the binding of *COX-2* to the promoter region of p65, a subunit of the NF-κB transcription factor, in the presence and absence of **4c**. For this study, CML cells were treated with compound **4c** (0, 5, 15, 30 µM), and the respective chromatin was fragmented, followed by immunoprecipitation using an antibody specific to *COX-2*. It was demonstrated that compound **4c** treatment markedly inhibited *COX-2* binding to the p65 promoter compared to the untreated control sample. It is therefore concluded that compound **4c** disrupted the interaction between *COX-2* and the p65 promoter and exerted inhibitory effects on the expression of genes regulated by NF-κB (Figure 4).

### 3.7. Frontier Molecular Orbitals (FMO) Calculations for the Title Compounds That Targets NF-κB

**Figure 5:**
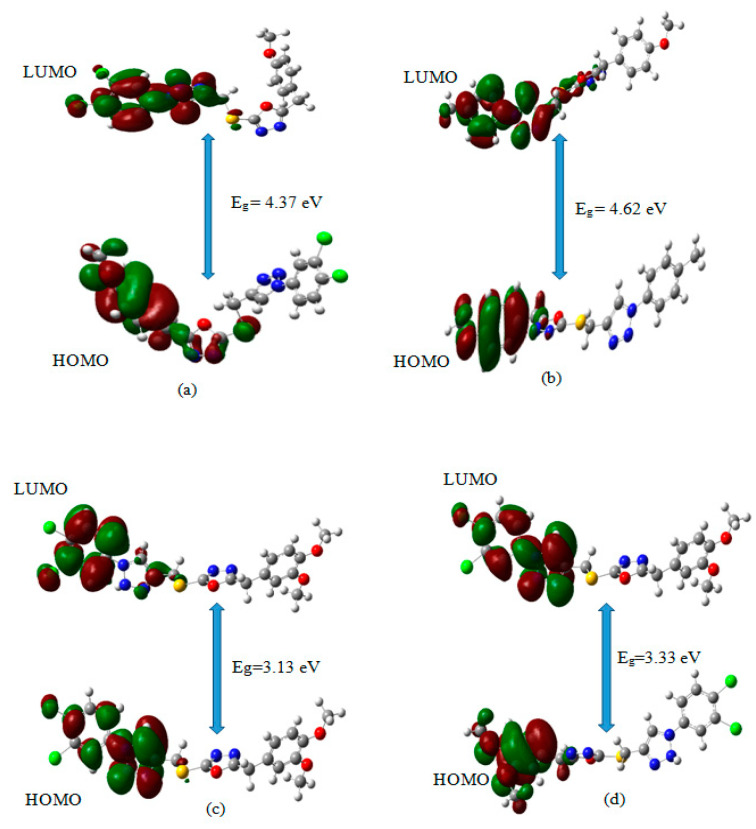
(**a**) **4c**, (**b**) (**4i**), and (**c**) and (**d**) alpha and beta molecular orbitals of (**4l**).

FMO analysis of compound **4c** was performed using the B3LYP method with a 6-311 (2d, p) basis set. The HOMO of a molecule represents the site where the electron density is highest and is involved in electron donation, whereas the LUMO represents the site where the electron density is lowest and is involved in electron acceptance. The E_LUMO_-E_HOMO_ energy gap is directly related to the molecule’s chemical reactivity and kinetic stability. The molecule has a smaller energy gap, which is attributed to less stability and more reactivity. In contrast, the molecule has a large energy gap, attributed to being less chemically reactive and having high chemical stability with greater hardness. The computed HOMO-LUMO energy gaps of **4c** and **4i** were found to be 4.37 eV and 4.62 eV, respectively. Thus, the title compounds had high stability, were less chemically reactive, and exhibited poor conductivity. The HOMO-LUMO energy gap of alpha and beta electrons of the **4l** was found to be 3.13 eV and 3.33 eV, respectively. It is observed that the HOMO-LUMO energy gap of **4l** decreased by 1.24 eV and 1.04 eV in alpha and beta orbitals, respectively, when compared with the **4c** molecule. The HOMO-LUMO plots of **4c**, **4i**, and also the alpha and beta molecular orbitals with the unpaired electrons of **4l** are depicted in Figure 5, indicating that HOMO orbitals were localized on the methoxybenzyl group and the oxadiazole ring except for the oxygen atom and sulphur atom. In contrast, LUMO orbitals were localized on dichlorophenyl, triazole groups, and sulphur atoms of the lead molecule **4c**. The FMO’s energy and chemical reactivity descriptor values were calculated and are summarized in Table 2. The chemical potential values for **4c** and **4i** were estimated to be −4.23 eV and −3.91 eV, respectively, and reflects their significant electron-accepting capacity. A high chemical potential indicates a strong electron-accepting ability, whereas a low chemical potential indicates a strong donating ability [46]. **4c** showed a high chemical hardness value (i.e., 2.18 eV^−1^) and a low value of chemical softness (0.22 eV^−1^). According to these findings, **4c** exhibited a lower tendency to exchange its electron cloud with the surrounding environment. A similar result was observed with **4i**, possessing chemical hardness and softness values of 2.312 eV and 0.216 eV^−1^. The electrophilicity index quantifies the energy required to stabilize a molecule, and it was found to be 4.100 eV, and 3.307 eV for **4c** and **4i**, respectively.

### 3.8. Molecular Electrostatic Potential (MEP) Analysis of Title Compounds

**Figure 6:**
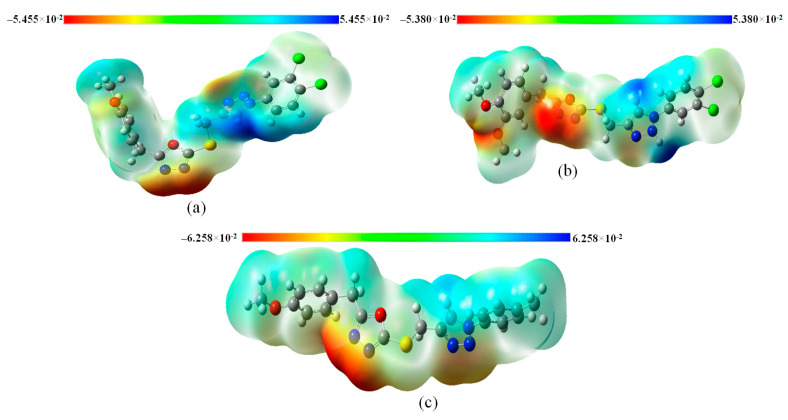
Molecular electrostatic potential map of (**a**) **4c**, (**b**) (**4l**), and (**c**) (**4i**) molecule.

Molecular electrostatic potential (MEP) analysis is used to determine the chemical reactivity of the molecule and predict the electrophilic and nucleophilic sites on the molecule and their intermolecular interactions. The MEP surfaces of **4c**, **4i**, and **4l** were generated from the optimized structure using the B3LYP method with a 6-311++G (d, p) basis set (Figure 6). The potential surface’s colors ranged from −5.455 × 10^−2^ to 5.455 × 10^−2^ a.u, −6.258 × 10^−2^ to 6.258 × 10^−2^ a.u., and −5.380 × 10^−2^ to 5.380 × 10^−2^ a.u. for **4c**, **4i**, and **4l** molecules, respectively. The electrostatic potential regions have been represented by different color codes: red represents the nucleophilic (i.e., region of negative electrostatic potential or electron rich), blue represents the electrophilic (i.e., region of positive electrostatic potential or electron deficient), and green denotes the region of zero potential. From Figure 6, the negative potential spots (−5.455 × 10^−2^ a.u.) in the red color regions are located on the nitrogen atom of the oxadiazole group, representing the most reactive site for the electrophilic attack. In contrast, the positive potential (5.455 × 10^−2^ a.u.) in the blue color regions localized at the hydrogen atom of the triazole group is the most reactive site for nucleophilic attack of the **4c** molecule. Similar electrophilic and nucleophilic attacks were observed in **4i** and **4l** molecules (Figure 6).

### 3.9. Natural Bond Orbital (NBO) Analysis

Natural bond orbital analysis is an efficient method for evaluating intra- and intermolecular bonding and anti-bonding interactions in molecular systems, and it also provides a useful platform for researching charge transfer or conjugative interactions. The second-order Fock matrix was employed in the NBO analysis to examine the donor–acceptor interactions [47]. For each donor (i) and acceptor (j), the stabilization energy E(2) is related to the delocalization i→j. The bigger the E(2) value, the more intense the interaction between electron donors and electron acceptors, i.e., the greater the giving tendency of electron donors to electron acceptors and the stronger the overall system’s conjugation. The possible intensive interactions are listed in Table 3. Electron density delocalization between occupied Lewis-type (bond or lone pair) orbitals and formally unoccupied non-Lewis orbitals corresponds to a stabilizing donor–acceptor interaction.

NBO analysis was performed for the **4c** molecule using the DFT/B3LYP/6-311++G (d, p) level of theory to determine the intra-molecular, rehybridization, and delocalization of electron density inside the molecule. The electron donation from the lone pair LP(2) to the anti-bonding acceptor (π *) is associated with the resonance in the molecule and contributes to its structural stability. LP (2) O12 → [π *(N10–C11) and π *(N10–C11)] interactions were observed with stabilization energies of 136.231 and 132.888 kJ/mol, respectively. The strong intra-molecular hyper-conjugative interaction of the σ and π electrons of C–C with the C–C anti-bonding orbital leads to the stabilization of the molecule. The results indicate that the most important intra-molecular charge transfer (ICT) in the molecule has a high stabilization energy. The most excellent E^(2)^ was found to be 1232.816 and 1104.492 kJ/mol for the conjugation of the molecule π (C1–C2) → [π *(C3–C4) and π *(C5–C6)] and 458.943, 359.154 and 195.099 kJ/mol for π (N17–C20) → [π *(C26–C27), π *(C23–C24) and π *(N18–N19)]. The strong intra-molecular hyper-conjugative interaction of N18-N19 from π (N17-C20) → π *(N18–N19), which increases the ED(j) value of 0.46421e, weakens the respective bonds, leading to a stabilization of 195.099 kJ/mol. The conjugative interactions LP (1) N9 → σ *(C8–O12) and LP (1) N10 → σ *(C11–O12) have the most significant electron donor occupancies of 1.933e and 1.93**4e**, which weaken the respective bonds with stabilization energies of 43.388 and 42.927 kJ/mol, respectively. The (N9–N10) atoms in the oxadiazole group have an electron-rich occupancy of 1.960e. It is observed in the σ(N9–N10) → [σ *(C11–S13) and σ *(C7–C8)] interaction along with stabilization energies of 29.12064 and 25.689 kJ/mol. The above-mentioned electron-rich occupation is also noticed in the MEP plot of **4c**. Further, the other interactions are listed in Table 3, which shows the intra-molecular charge transfer of the **4c** molecule corresponding to the stabilization of the molecule.

### 3.10. Molecular Docking and Dynamics Analysis of Compound ***4c*** That Targets NF-κB

**Figure 7:**
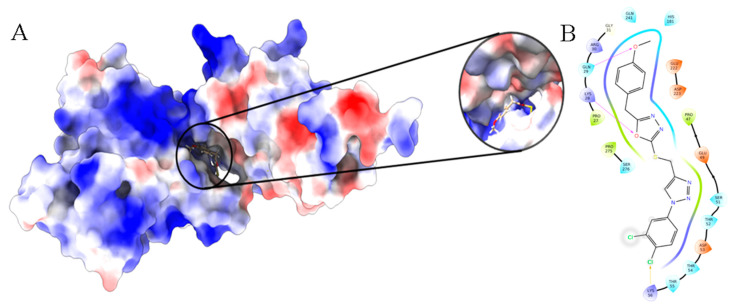
(**A**) The binding pose of **4c** in the active site of the p65 subunit of NF-κB and (**B**) The 2D interaction plot of **4c** in the binding pocket of p65.

Molecular docking studies were performed to identify the specific regions of the p65 subunit of NF-κB that interacted with **4c** by using reported methods [48]. Figure 7A illustrates the binding pose of the ligand molecule in the active site of the p65 subunit of NF-κB. Figure 7B displays a 2D interaction plot of the ligand in the binding pocket of p65. The docking resulted in a binding score of −9.13 kcal/mol. The oxygen atoms of the 1-methoxy-4-methylbenzene group and 1,3,4-oxadiazole groups in **4c** have been linked to GLN29 and LYS28 amino acids via hydrogen bonding with a length of 2.03 Å and 2.06 Å, respectively. Further, the chlorine atom of 1,2-dichlorobenzene has formed halogen interactions with LYS56. The complex’s stability is further enhanced by the hydrophobic interaction in the active site of the protein. Because of these interactions with amino acids, precise interactions with DNA are prohibited. This results in the synthesized **4c** acting as a potent inhibitor for the transcription factor NF-κB.

**Figure 8:**
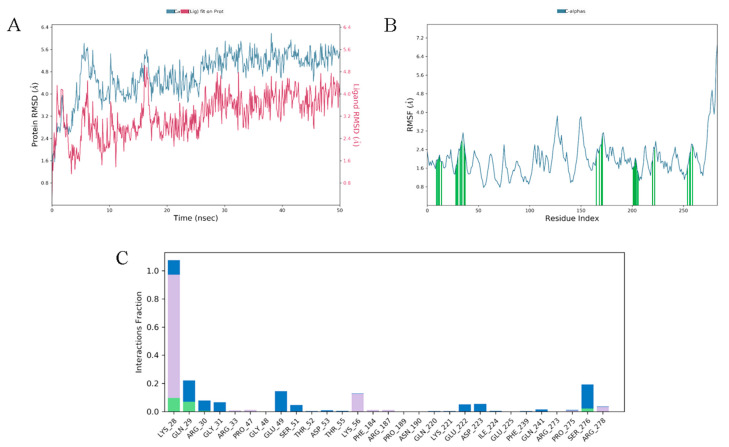
(**A**) The root-mean-square deviations (RMSDs) of **4c** with respect to p65 subunit of NF-κB; (**B**) RMSF plot of protein molecule (NF-κB); and (**C**) protein–ligand contacts map.

Molecular dynamics simulations were performed to determine the stability of the compound in the p65 subunit for 50 ns, along with other coordinates associated with the trajectory file. Figure 8A depicts the root-mean-square deviations (RMSDs) of **4c** concerning its target and binding pocket (red colored) with respect to protein (blue color). It was observed that the values of ligand RMSD were lower than protein RMSD throughout the simulation time, especially after 10 ns, when the ligand in the active site is bound firmly and there are no fluctuations. Root mean square fluctuation (RMSF) observations (Figure 8B) depict the changes along protein chains. It was observed that the fluctuations of residues interacting with the ligand atoms were considerably reduced. Figure 8C explains the interaction contacts of proteins concerning the ligand. The hydrogen bond was formed with the residues LYS28, GLN29, ARG30, and SER276. Out of which, the formation of hydrogen bonds was present with LYS28 for about 20% of the simulation time, and later it was mediated by hydrophobic and water bridges. This resulted in a strong H-bond and the stability of the molecule within the pocket site.

### 3.11. Compound ***4c*** Induces Apoptosis in CML Cells

**Figure 9:**
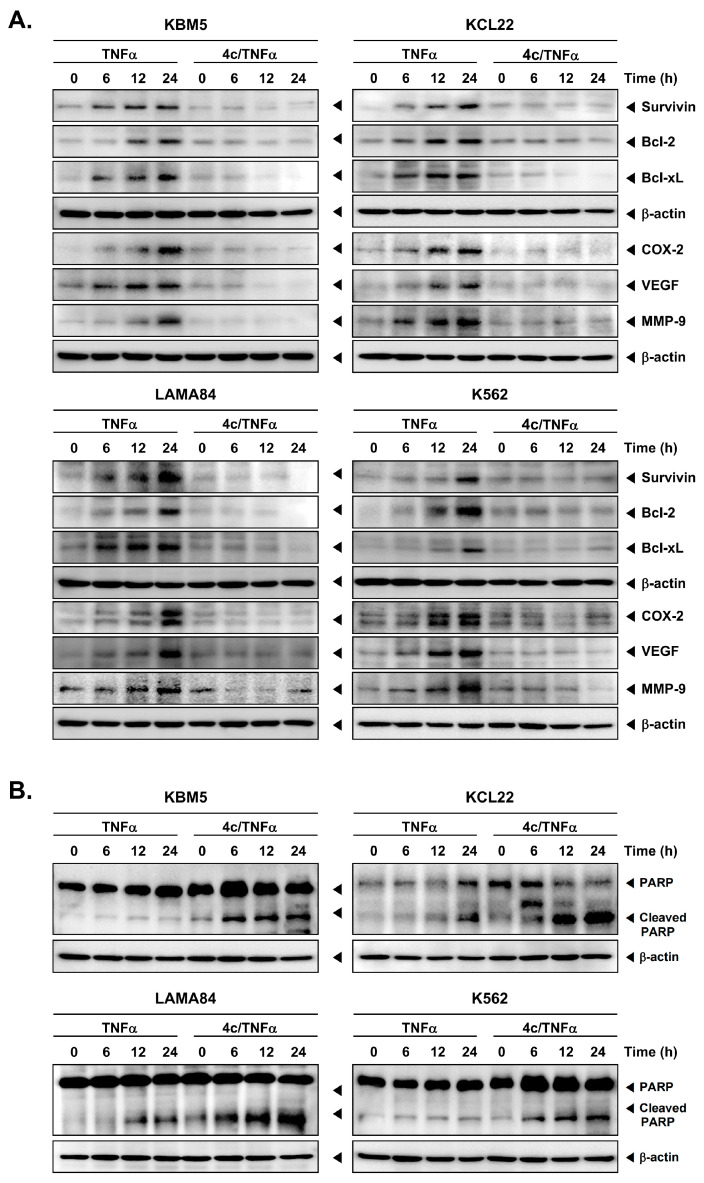
Induction of apoptosis by compound **4c**. (**A**) Western blot analysis was performed to determine the expression of various oncogenic proteins. (**B**) Expression of PARP was evaluated by Western blot analysis. The results shown are representative of at least three independent experiments.

A number of drugs that target NF-κB activation decrease the expression of anti-apoptotic and oncogenic genes in CML cells. The decrease in the expression levels of survivin, MMP-9, Bcl-2/xl, VEGF, and COX-2 suggests that the NF-κB targeting drugs could modulate the survival and growth of CML cells by promoting apoptosis. Therefore, we investigated the effect of compound **4c** on apoptosis by measuring the protein expression of various NF-κB regulated genes. For this purpose, CML cells were pretreated with **4c** (0 or 30 µM), and incubated with TNFα (0.5 nM) for 0, 6, 12, and 24 h.. The results of the study revealed that TNFα induced the expression of various anti-apoptotic proteins in CML cells whereas compound **4c** suppressed the expression of these proteins (Figure 9A).

The cleavage of PARP (poly ADP-ribose polymerase) ultimately leads to apoptosis. Therefore, **4c**-induced PARP cleavage was determined using Western blot analysis. For this purpose, CML cells were pretreated with compound **4c** (0 or 30 µM) and incubated with TNFα (0.5 nM) for 0, 6, 12, and 24 h. Compound **4c** enhanced the cleavage of PARP in CML cells which indicated that it increased apoptosis of these cells (Figure 9B). 

### 3.12. Compound ***4c*** Induced Apoptotic-Cell Death in CML Cells

**Figure 10:**
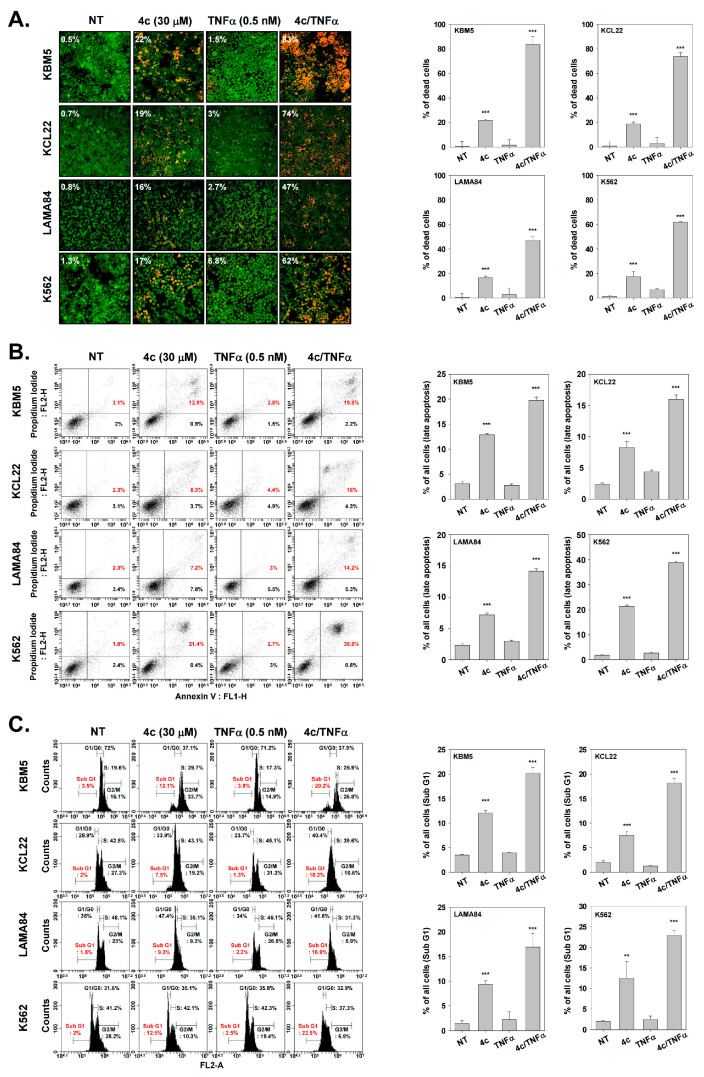
Promotion of apoptosis by **4c** in CML cells. (**A**) Apoptotic cells were observed by live and dead assay. (**B**) Apoptotic cells were analyzed by Annexin V assay. (**C**) Cells were stained with PI, and cell cycle was analyzed by flow cytometry. *** *p* < 0.001 vs. non-treated (NT) cells; ** *p* < 0.01 vs. non-treated (NT) cells. The results shown are representative of at least three independent experiments.

Next, live and dead assays were performed following treatment with compound **4c**. CML cells were treated with compound **4c** (0 or 30 µM) alone or with TNFα (0.5 nM) in combination, and the fluorescent signal was observed, which showed an increased percentage of red signal in CML cells. Additionally, **4c** and TNFα combined treatment displayed an enhanced effect on cell death, resulting in a much higher percentage of dead cells than **4c** alone (Figure 10A).

In another assay, CML cells were treated with **4c** and TNFα and the apoptotic cells were determined by using Annexin V and FITC/PI in FACS. In the scatter plot, the upper right panel showed late apoptotic cells, and the lower right panel showed early apoptotic cells. In CML cells, treatment with **4c** mainly increased late apoptotic cells more than early apoptotic cells, which was further augmented in the presence of TNFα (Figure 10B).

Finally, an effect of **4c** on cell cycle arrest was also observed. Cell cycle analysis showed that compound **4c** arrested the cell cycle, and cells were accumulated in the sub G1 phase before the G0/G1 phase (Figure 10C).

## 4. Discussion

The representative characteristic of CML cells is the presence of the Philadelphia chromosome (Ph), which arises due to the reciprocal translocation of the (9;22) chromosome and produces BCR-ABL [49]. When CML enters the myeloid blast crisis phase, BCR-ABL promotes disease progression. A number of small molecule kinase inhibitors, which inhibit BCR-ABL, have been successful to control CML progression [50,51]. However, resistance to these inhibitors may develop in some patients [52]. The reports of the development of drug resistance indicate the necessity of developing further therapeutic strategies for CML patients. Therefore, the goal of this study was to investigate the possible effects of **4c** on NF-κB in human CML cells.

First, we confirmed cell viability at selected cancer cell-specific toxic concentrations and evaluated the inhibitory effect of NF-κB activity in CML cells at selected concentrations. NF-κB is a complex of p50, p65, and IκBα, and remains under unstimulated conditions in the cytoplasm [53]. When upstream kinases such as IκB kinase (IKK) are activated and IκBα is ubiquitinated as well as degraded, NF-κB is released into the nucleus to initiate the transcription of various genes involved in tumorigenesis. Therefore, we investigated whether **4c** could inhibit the oncogenic activity of CML cells by targeting NF-κB. **4c** reduced the activity of NF-κB by inhibiting its binding to DNA and translocation into the nucleus. Moreover, it was confirmed that **4c** modulated the activation of upstream kinases rather than simply reducing the activity of NF-κB. First, **4c** inhibited expression of p-IKKα/β, and then also inhibited p-IκBα, which activates NF-κB by being degraded through phosphorylation. Thereafter, the protein expression levels of p65 and p-p65 were also found to be decreased by **4c**.

**Figure 11:**
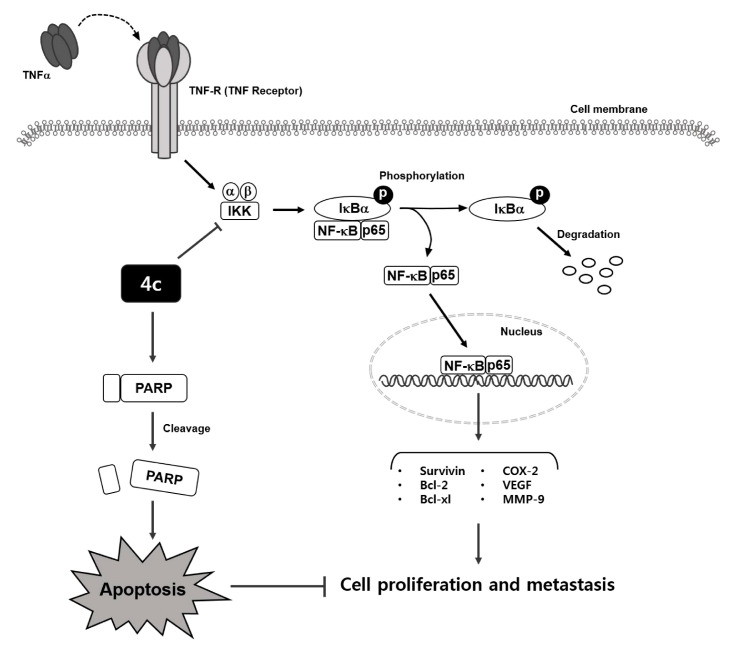
A schematic diagram depicting the possible mechanism through which compound **4c** targets TNFα-induced NF-κB inactivation in CML cells.

Cancer cells often undergo apoptosis upon exposure to therapeutic agents by modulation of survival protein function [54]. Hence, herein the inhibitory effect of **4c** on the expression of survivin, MMP-9, VEGF, Bcl-2/xl, and COX-2 was observed, with **4c** reducing the expression of these proteins even in presence of TNFα. It was also confirmed that PARP cleavage was induced upon treatment with **4c**. Also, **4c** enhanced apoptosis of CML cells as evidenced in Annexin V assays. Compound 4c therefore promotes loss of cell viability by multiple downstream mechanisms (Figure 11) and could potentially serve as a hit to develop a molecule abrogating the development of resistance in CML. 

## 5. Conclusions

In conclusion, this study demonstrated that compound **4c** can effectively inhibit NF-κB activation while inducing apoptosis in CML cells. Compound **4c** not only regulates NF-κB but also upstream kinases, and regulation of these molecular mechanisms by **4c** may contribute to the development of novel therapies for CML.

## Figures and Tables

**Table 1:** Effect of newly synthesized oxadiazole and triazole bridged with methylene-thiol derivatives on viability of MCF-7 cell line.

Compound	R_1_	R_2_	IC_50_ (µM)
**4a**	4-methoxy	4-Cl	9.58 ± 0.98
**4b**	4-methoxy	4-Br	8.44 ± 0.93
**4c**	4-methoxy	3,4-Cl_2_	7.40 ± 0.87
**4d**	4-methoxy	H	25.80 ± 1.41
**4e**	4-methoxy	3-Cl	16.86 ± 1.23
**4f**	4-methoxy	4-methoxy	34.50 ± 1.54
**4g**	4-methoxy	4-OH	29.73 ± 1.47
**4h**	4-methoxy	2-CH_3_	26.22 ± 1.42
**4i**	4-methoxy	4-CH_3_	45.39 ± 1.66
**4j**	3,4-dimethoxy	4-Cl	40.53 ± 1.61
**4k**	3,4-dimethoxy	4-Br	31.90 ± 1.50
**4l**	3,4-dimethoxy	3,4-Cl_2_	23.79 ± 1.38
**4m**	3,4-dimethoxy	H	28.26 ± 1.45
**4n**	3,4-dimethoxy	3-Cl	23.16 ± 1.37
**4o**	3,4-dimethoxy	4-methoxy	35.83 ± 1.55
**4p**	3,4-dimethoxy	4-OH	65.04 ± 1.81
**4q**	3,4-dimethoxy	4-CH_3_	19.73 ± 1.30
**4r**	3,4-dimethoxy	2-CH_3_	35.70 ± 1.55
Tamoxifen			1.45 ± 0.16
Doxorubicin			0.91 ± 0.04

**Table 2:** HOMO-LUMO energy values of active compounds.

Properties	4c	(4i)	(4l)	
			Alpha Orbitals	Beta Orbitals
E_HOMO_ eV	−6.4199	−6.2245	−4.5581	−5.8096
E_LOMO_ eV	−2.0479	−1.5989	−1.4182	−2.4781
∆E_LUMO−HOMO_ eV	4.372	4.6256	3.1399	3.3313
Ionization potential (I)	6.4199	6.2245	4.5581	5.8096
Electron affinity (A)	2.0479	1.5989	1.4182	2.4781
Global hardness (ɳ)	2.1860	2.3128	1.5699	1.6656
Softness (S)	0.2287	0.2161	0.3184	0.3001
Chemical potential (μ)	−4.2339	−3.9117	−2.9881	−4.1438
Electronegativity (χ)	4.2339	3.9117	2.9881	4.1438
Electrophilicity (ᴪ)	4.1001	3.3079	2.8437	5.1546

**Table 3:** Second-order perturbation theory analysis of Fock matrix (F (i,j) ^b^ a.u) at 6-311++G (d, p) basis level corresponding to the intra-molecular interactions of the **4c** molecule.

Donor (i)	ED (i) (e)	Acceptor (j)	ED (j)(e)	E^(2)^ kJ/mol	∆E ^a^	F (i,j) ^b^ a.u.
π (C1–C2)	0.38305	π *(C3–C4)	0.30446	1232.816	0.01	0.083
	0.38305	π *(C5–C6)	0.35079	1104.492	0.01	0.08
π (N17–C20)	0.90032	π *(C26–C27)	0.37632	458.943	0.07	0.1
	0.90032	π *(C23–C24)	0.32671	359.1546	0.08	0.097
	0.90032	π *(N18–N19)	0.46421	195.0999	0.03	0.043
	1.62866	π *(C15–C16)	0.33318	98.57504	0.36	0.083
LP (1) C25	1.07521	π *(C26–C27)	0.37632	254.2198	0.16	0.103
	1.07521	π *(C23–C24)	0.32671	248.6133	0.17	0.107
π (N10–C11)	0.30658	π *(C8–N9)	0.24318	202.4219	0.02	0.054
π (N18–N19)	0.46421	π *(C15–C16)	0.33318	201.9617	0.05	0.069
π (C23–C24)	1.67802	π *(N17–C20)	0.90032	142.0468	0.2	0.088
LP (2) O12	1.71189	π *(N10–C11)	0.30658	136.231	0.35	0.095
	1.71189	π *(C8–N9)	0.24318	132.8838	0.37	0.097
LP (2) O21	1.84250	π *(C1–C2)	0.38305	126.0639	0.34	0.096
π (C26–C27)	1.67623	π *(N17–C20)	0.90032	125.5618	0.21	0.085
	0.37632	π *(C23–C24)	0.32671	43.97384	0.01	0.016
π (C15–C16)	1.76426	π *(N18–N19)	0.46421	109.7882	0.25	0.076
π (N17–C20)	1.62866	π *(N18–N19)	0.46421	104.6000	0.31	0.08
π (N17–C20)	1.62866	π *(C15–C16)	0.33318	98.57504	0.36	0.083
π (C1–C2)	1.66747	π *(C5–C6)	0.35079	91.33672	0.3	0.072
π (C3–C4)	1.70804	π *(C1–C2)	0.38305	89.87232	0.28	0.071
π (C5–C6)	1.69157	π *(C3–C4)	0.30446	87.61296	0.29	0.069
LP (2) S13	1.86175	π *(N10–C11)	0.30658	72.46688	0.24	0.06
π (C5–C6)	1.69157	π *(C1–C2)	0.38305	72.13216	0.28	0.063
π (C8–N9)	1.90918	π *(N10–C11)	0.30658	44.64328	0.32	0.056
LP (1) N9	1.93333	σ *(C8–O12)	0.06577	43.38808	0.72	0.077
LP (1) N10	1.93465	σ *(C11–O12)	0.07854	42.92784	0.71	0.076
σ (N9–N10)	1.96066	σ *(C11–S13)	0.03347	29.12064	0.96	0.073
	1.96066	σ *(C7–C8)	0.02796	25.68976	1.18	0.076
σ (C15–C16)	1.97644	σ *(N17–C20)	0.03907	25.3132	1.10	0.073
σ (C14–H37)	1.98275	σ *(C15–C16)	0.02903	21.84048	1.09	0.068
σ (C24–C25)	1.97360	σ *(C26–Cl28)	0.02784	20.0832	0.89	0.058

E^(2)^ is the stabilization energy in KJ/mol. ^a^ Energy difference between donor orbital i and acceptor j orbital ∆E = E(i) − E(j) a.u. ^b^ F(i,j) is the Fock matrix element between i and j NBO orbitals in a.u. unit. * represents anti-bonding molecular orbital.

## Data Availability

All data are freely available with this article.

## Supplementary Materials

The following supporting information can be downloaded at: https://www.mdpi.com/article/10.3390/biomedicines11061662/s1, Supplementary Data contains NMR, LCMS and IC_50_ of all the synthesized compounds **4(a-r)**.
