# Supplementary material for: Methyl-Thiol-Bridged Oxadiazole and Triazole Heterocycles as Inhibitors of NF-κB in Chronic Myelogenous Leukemia Cells
Source: Biomedicines. 2023 Jun 8;11(6):1662. doi: 10.3390/biomedicines11061662 (PMC10296415; doi:10.3390/biomedicines11061662)
Supplement: Supplementary file 1 [file biomedicines-11-01662-s001.zip › biomedicines-2363167-supplementary.pdf]

Methyl-thiol-bridged oxadiazole and triazole heterocycles as inhibitors of NF-kB in chronic myelogenous leukemia cells

Basappa Basappa,Young Yun Jung, Akshay Ravish, Zhang Xi, Ananda Swamynayaka, Mahendra Madegowda, Vijay Pandey, Peter E. Lobie, Gautam Sethi, Kwang Seok Ahn and

Supplementary Data

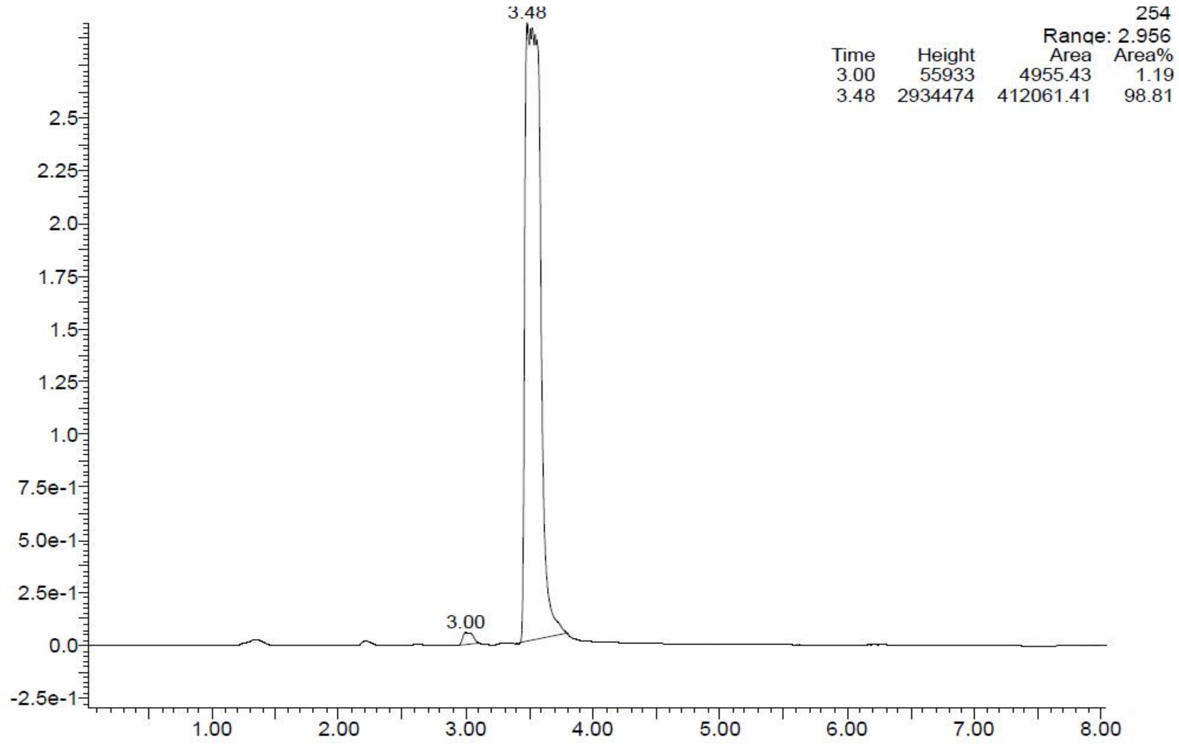

Liquid chromatogram of **2**

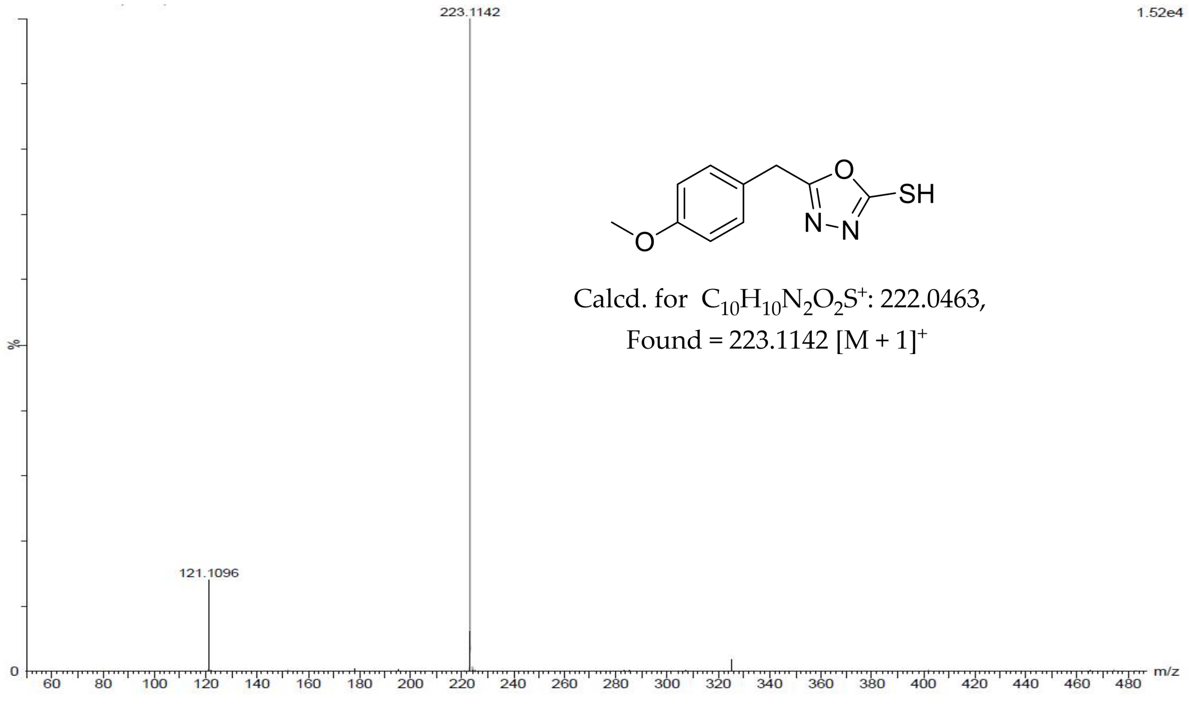

Mass spectra of **2**

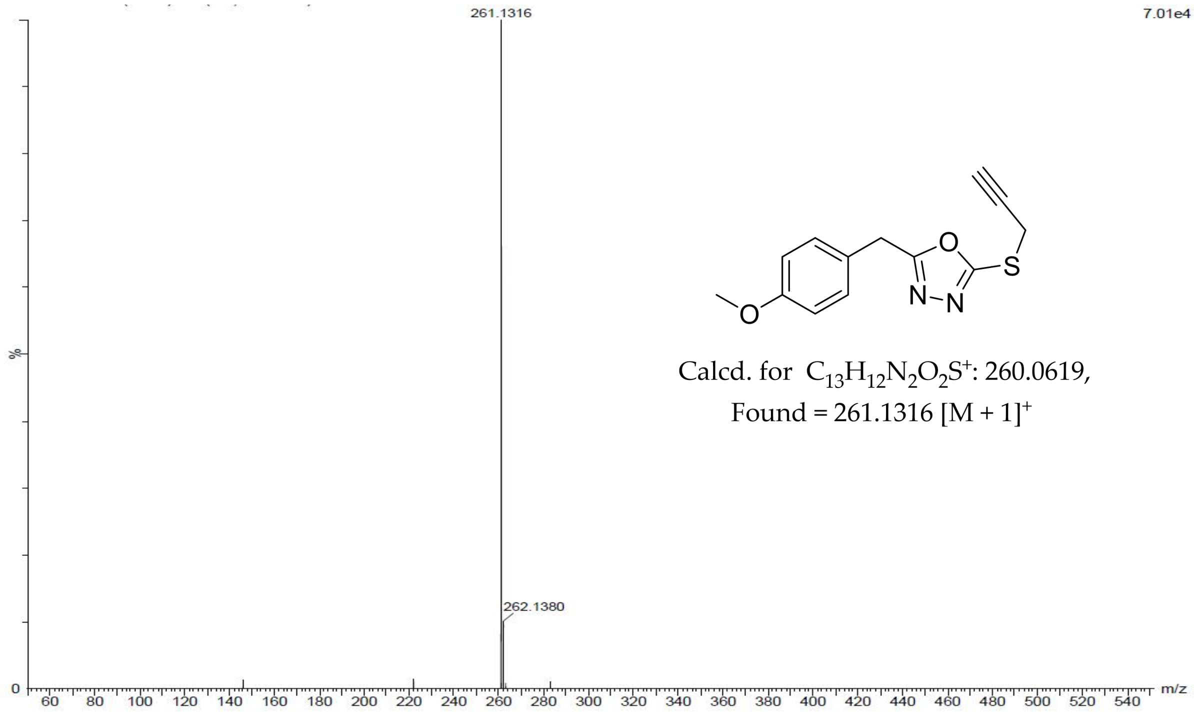

Mass spectra of **3**

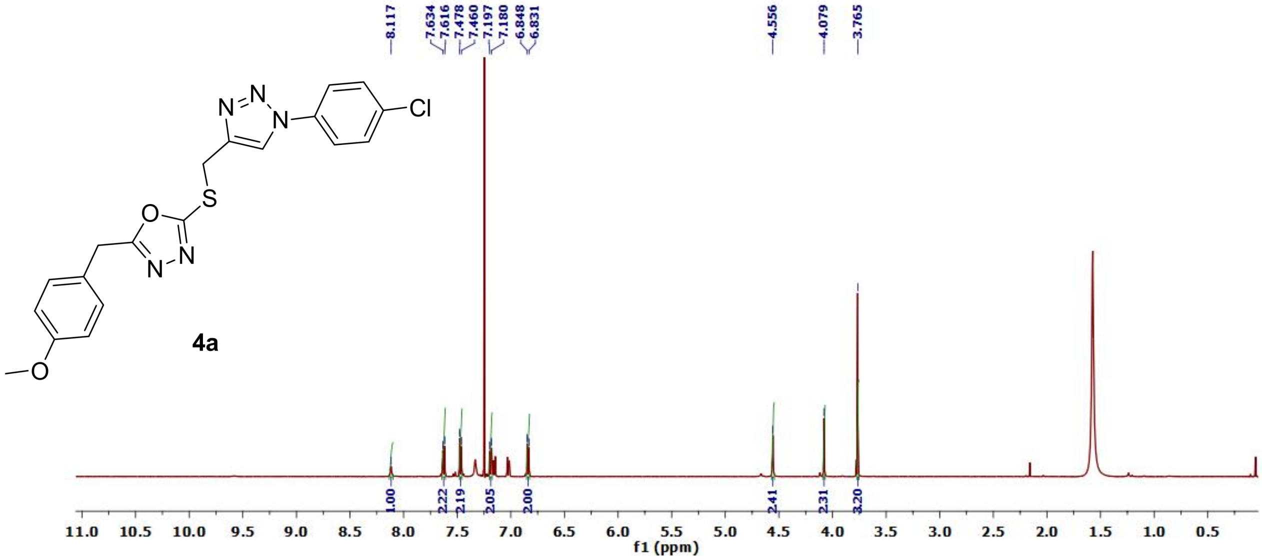

^{1}H NMR of **4a**

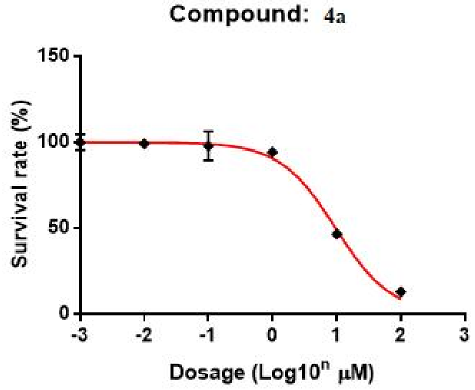

IC_{50} (μM) = 9.58
Log IC_{50} (μM) = 0.98

- Cell line: MCF7 (2000 cells/per well[96])
- Treated time: 72hrs
- Assay: alamarBlue (4hrs incubated)
- Data: 4a

Conc. (μM)	Viability	
	AVE.	± SD.
0	100.00	4.56
0.01	99.04	2.24
0.1	97.79	8.53
1	94.16	2.86
10	46.46	2.84
100	12.78	0.19

Cytotoxicity assay of **4a**

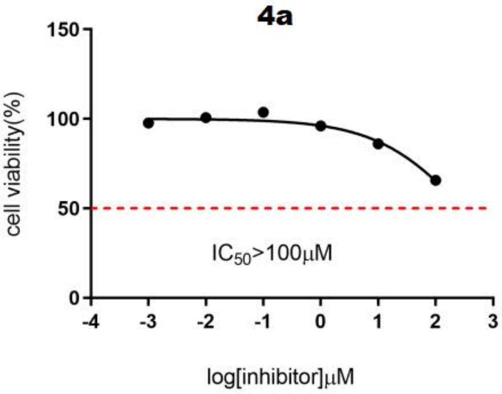

- Cell line: MCF10A(2000cells/per well 96-well plate)
- Treated time: 72hrs
- Assay: alamarBlue(4hrs incubated) N=3

Conc.(μM)	Viability	
	AVE.	±SD
0	100.00	2.52
0.01	100.67	1.15
0.1	103.67	1.53
1	96.00	1.00
10	86.00	2.65
100	65.67	1.53

Cytotoxicity assay for the compound **4a**

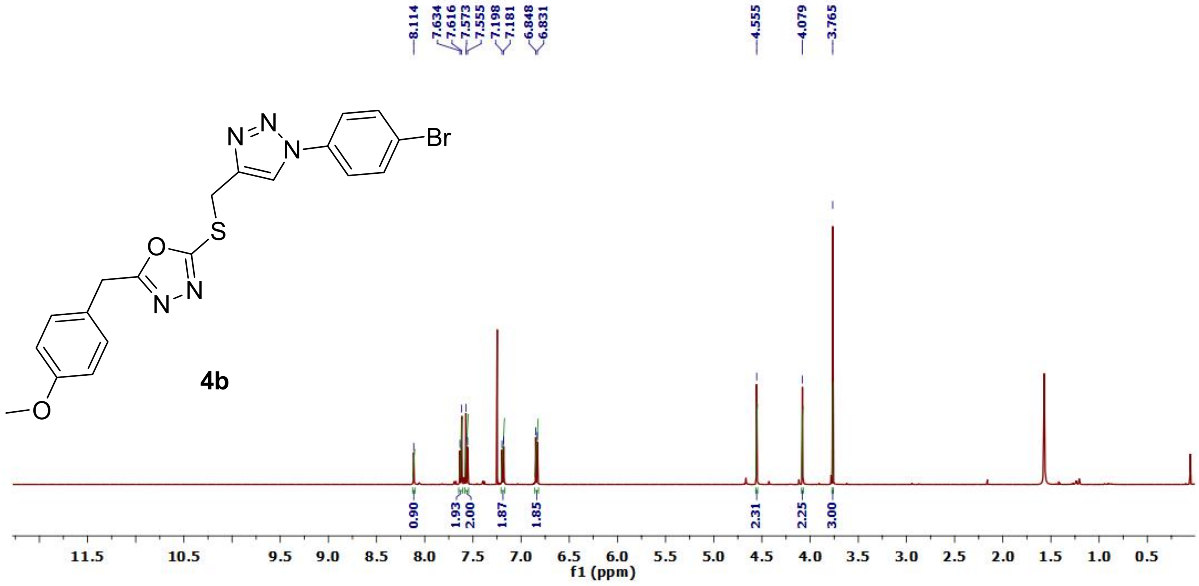

^{1}H NMR of **4b**

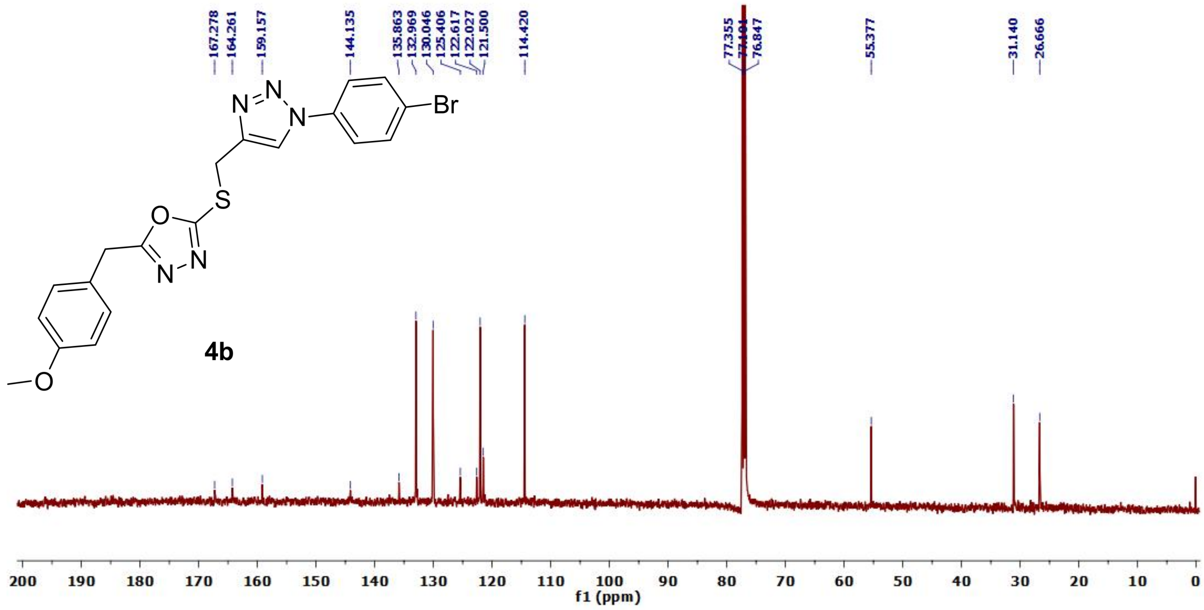

^{13}C NMR of **4b**

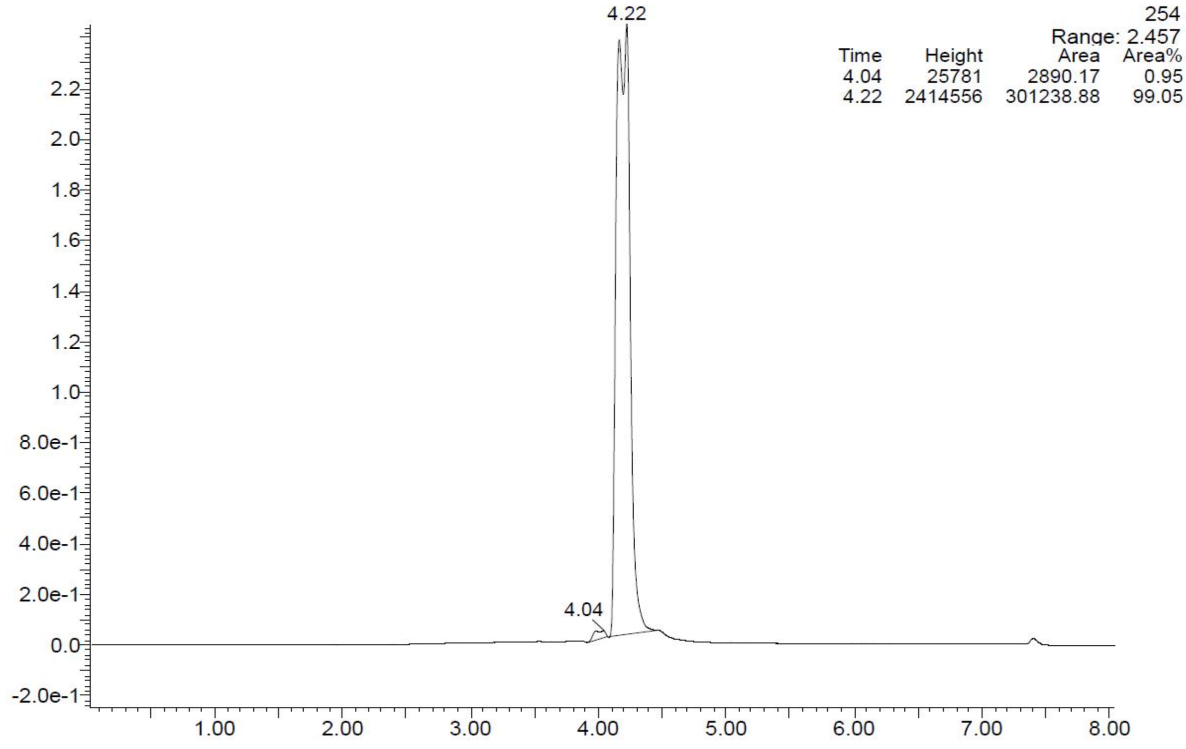

Liquid chromatogram of **4b**

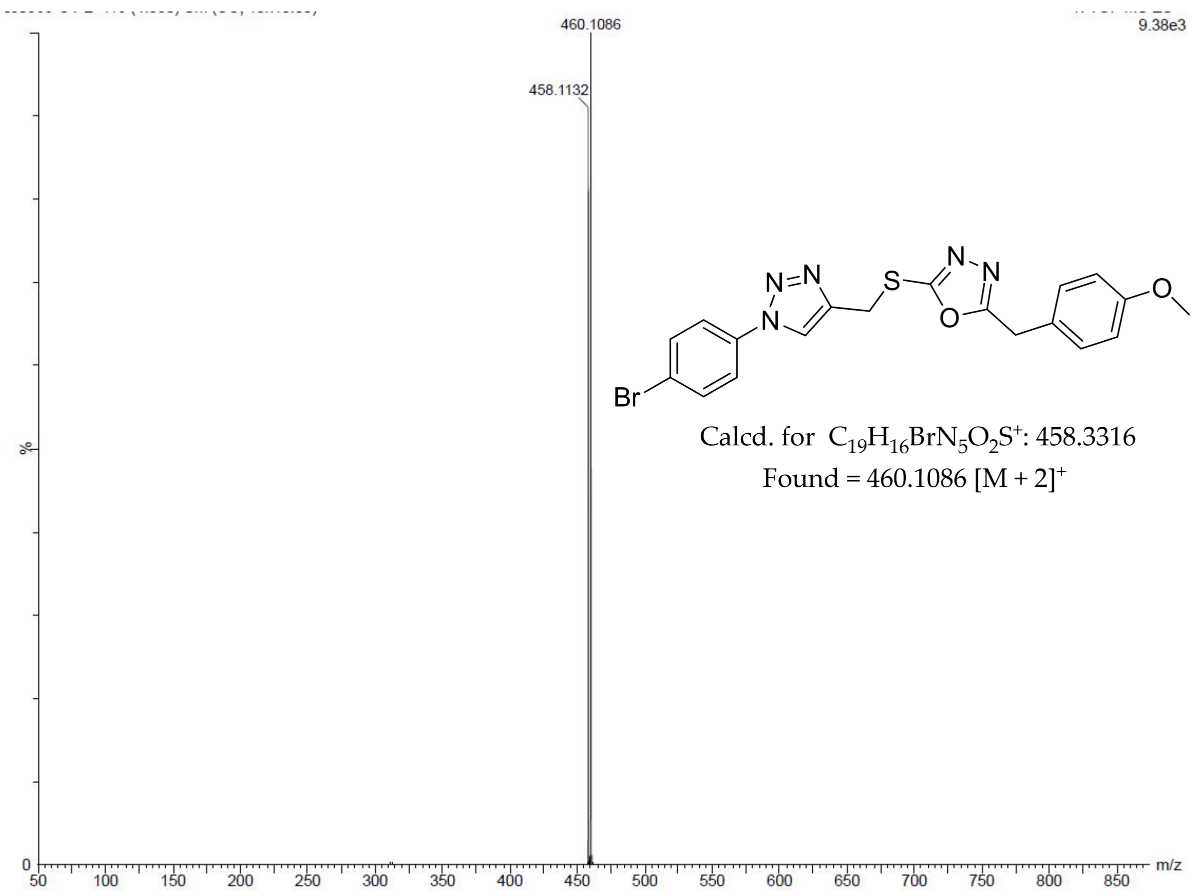

Mass spectra of **4b**

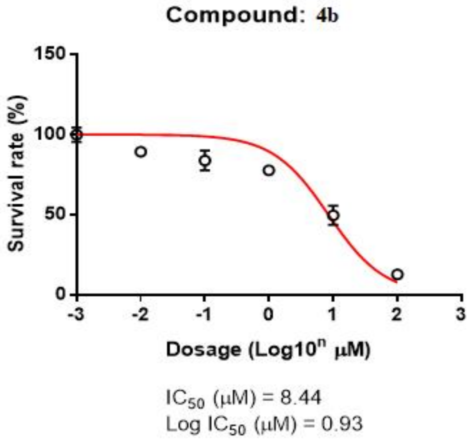

- **Cell line: MCF7 (2000 cells/per well[96])**
- **Treated time: 72hrs**
- **Assay: alamarBlue (4hrs incubated)**
- **Data: 4b**

**Conc. (μM)**	**Viability**	
	**AVE.**	**± SD.**
**0**	100.00	4.56
**0.01**	89.28	1.80
**0.1**	83.87	6.15
**1**	77.69	2.03
**10**	49.55	6.07
**100**	12.73	0.24

Cytotoxicity assay of **4b**

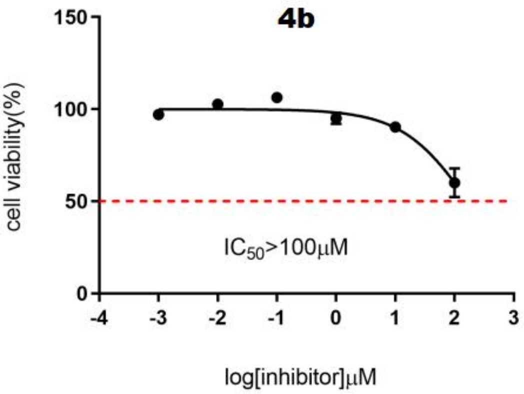

Conc.(μM)	Viability	
	AVE.	±SD
0	100.00	1.73
0.01	102.67	1.15
0.1	106.33	2.31
1	95.00	3.00
10	90.33	0.58
100	60.00	7.81

Cytotoxicity assay for the compound **4b (MCF-10A)**

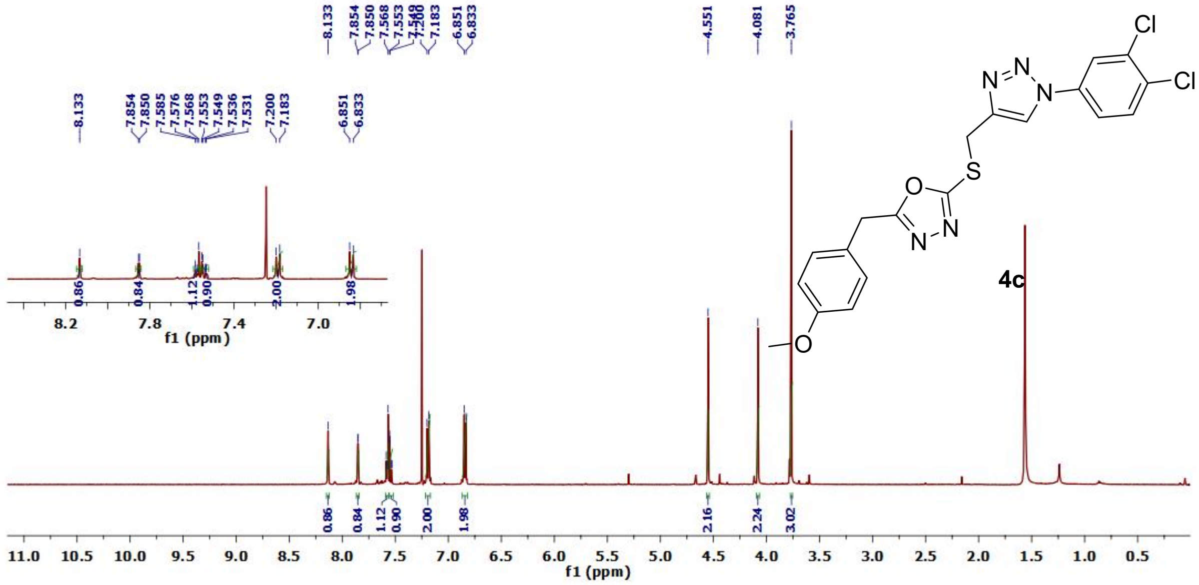

^{1}H NMR of **4c**

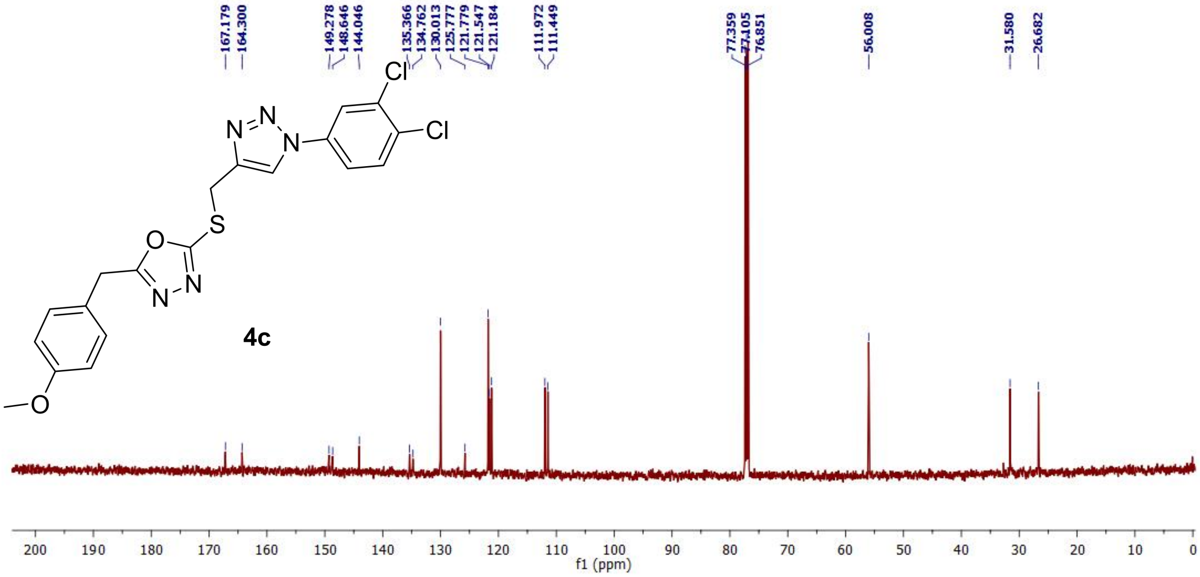

^{13}C NMR of **4c**

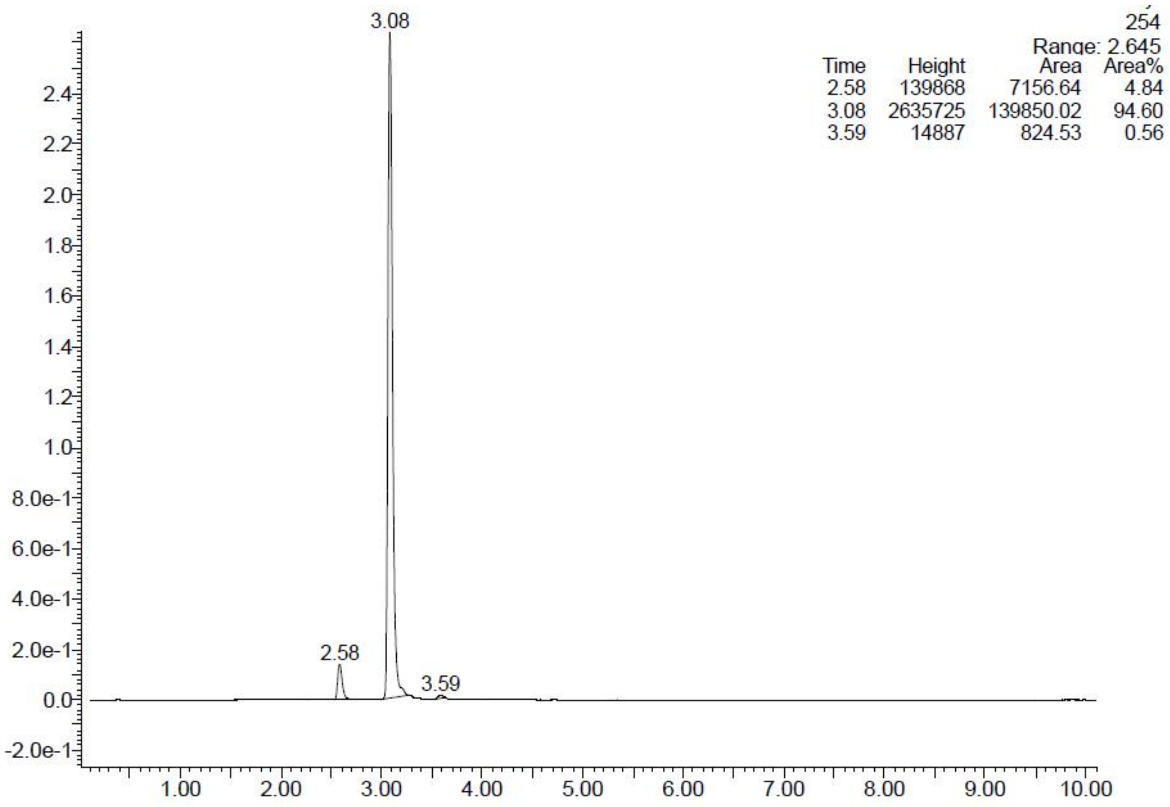

Liquid chromatogram of **4c**

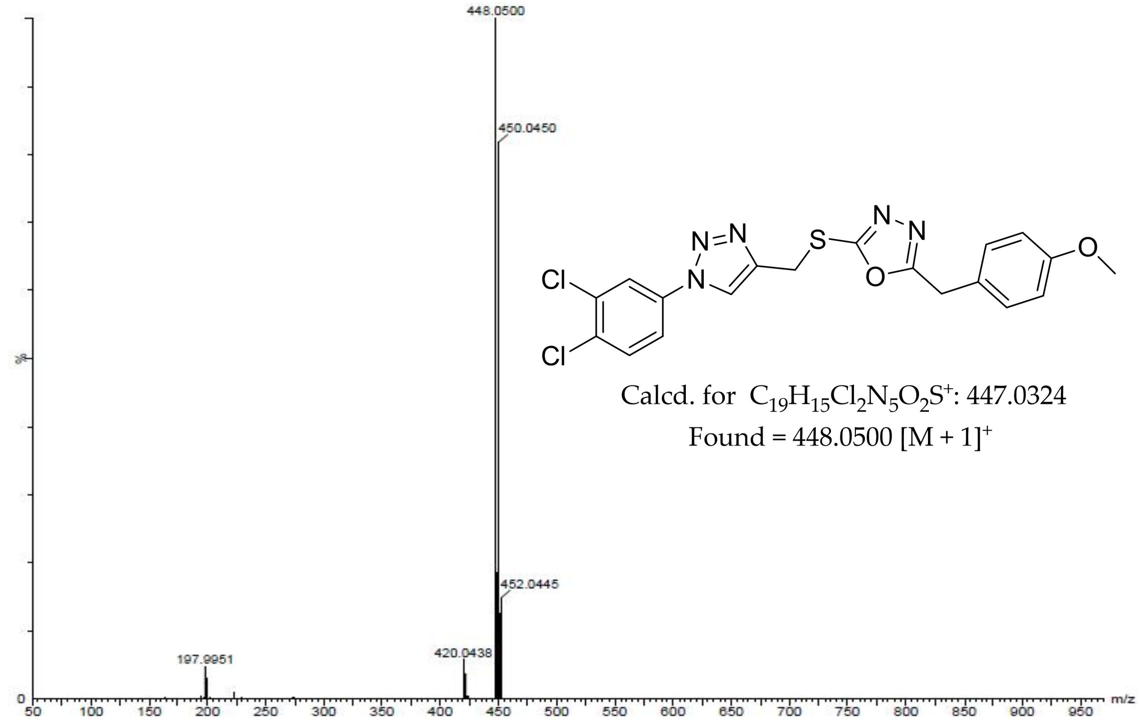

Mass spectra of **4c**

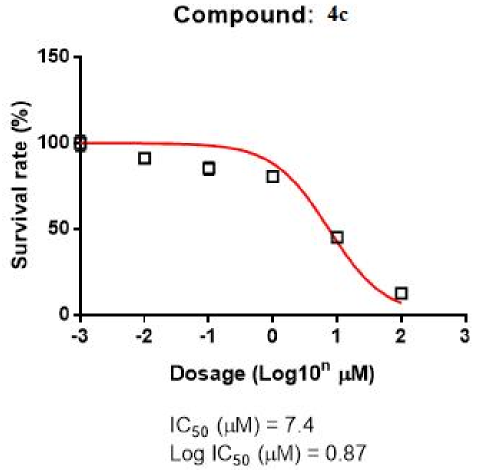

- Cell line: MCF7 (2000 cells/per well[96])
- Treated time: 72hrs
- Assay: alamarBlue (4hrs incubated)
- Data: **4c**

Conc. (μM)	Viability	
	AVE.	± SD.
**0**	100.00	4.56
**0.01**	91.20	2.49
**0.1**	85.17	3.49
**1**	80.40	2.80
**10**	45.19	1.37
**100**	12.54	0.18

Cytotoxicity assay of **4c**

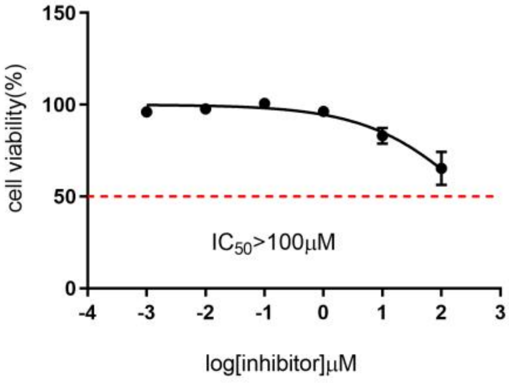

Conc.(μM)	Viability	
	AVE.	±SD
0	100.00	1.73
0.01	97.67	2.52
0.1	100.67	2.52
1	96.33	1.53
10	83.00	4.36
100	65.33	9.02

Cytotoxicity assay for the compound **4c (MCF-10A)**

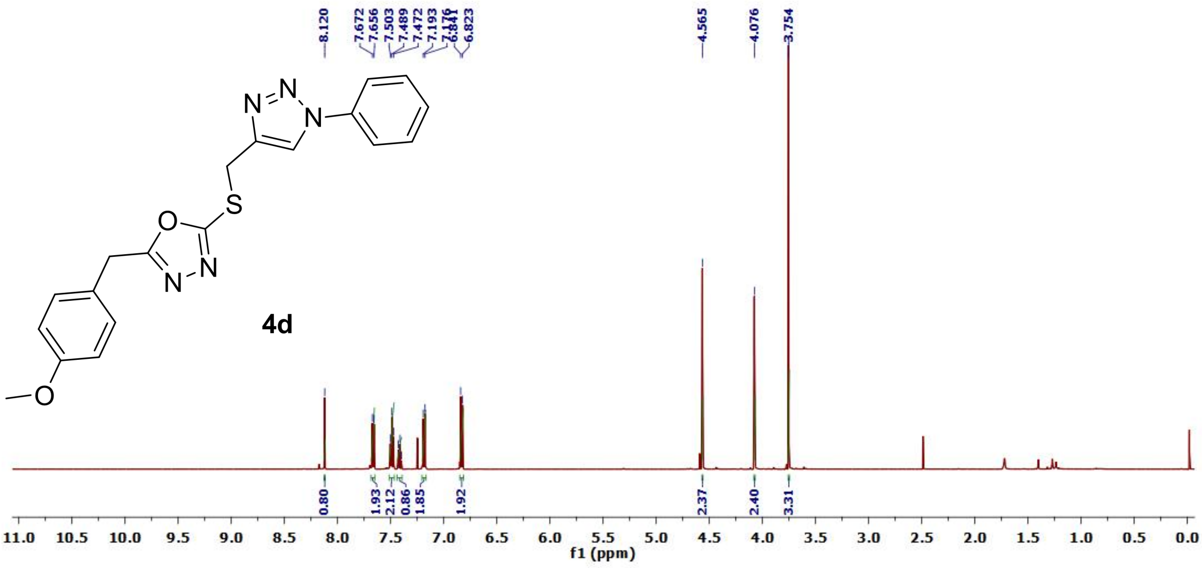

^{1}H NMR of **4d**

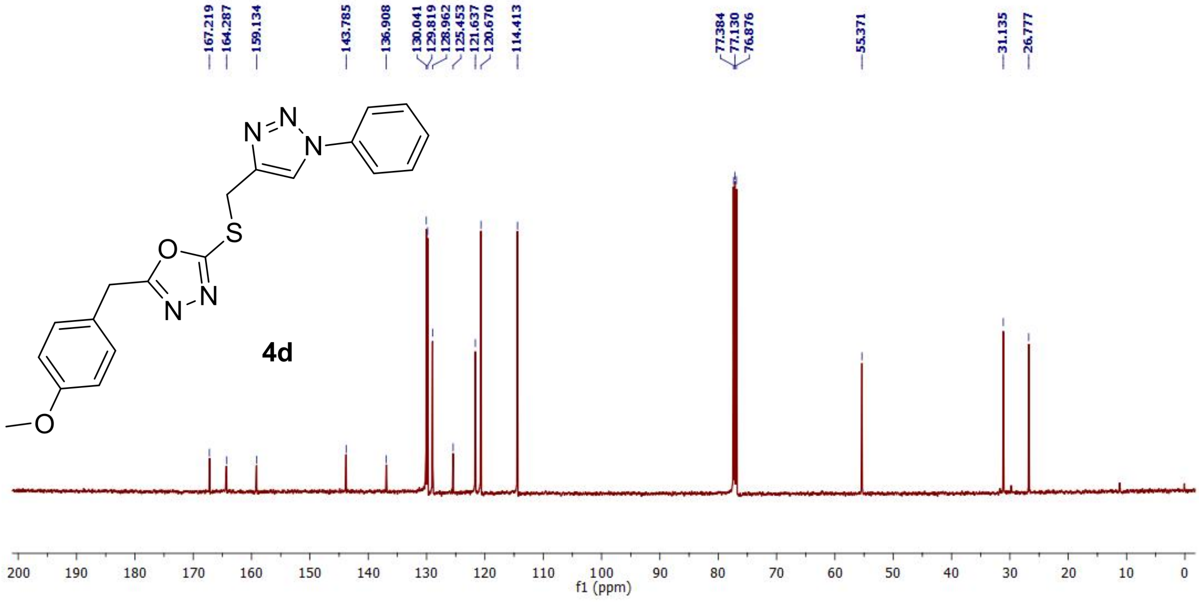

^{13}C NMR of **4d**

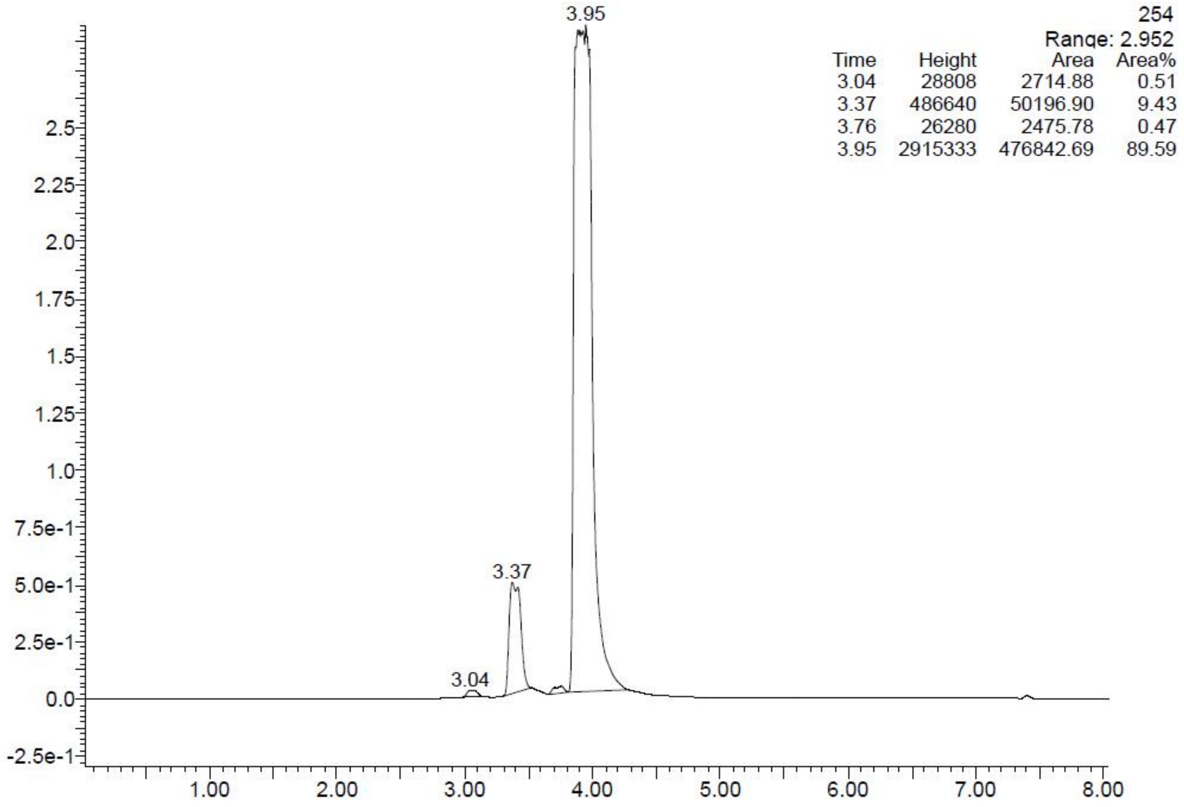

Liquid chromatogram of **4d**

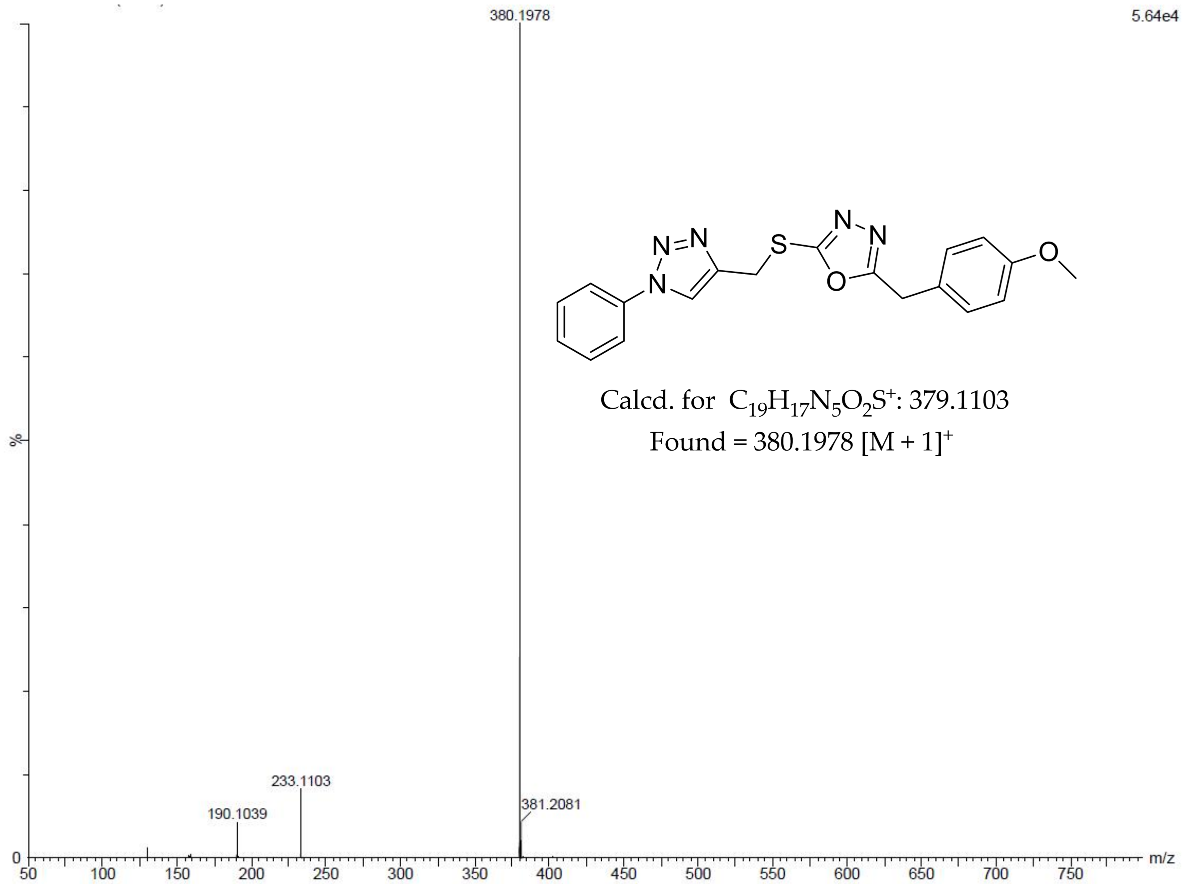

Mass spectra of **4d**

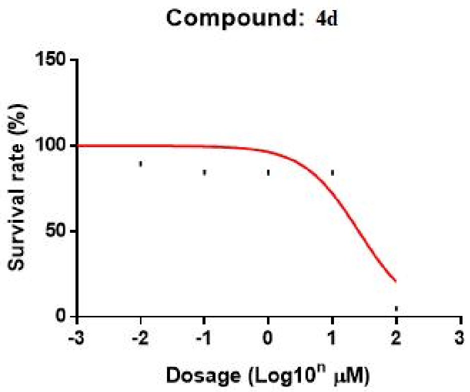

IC$_{50}$ (μM) = 25.80
Log IC$_{50}$ (μM) = 1.41

- Cell line: MCF7 (2000 cells/per well[96])
- Treated time: 72hrs
- Assay: alamarBlue (4hrs incubated)
- Data: 4d

Conc. (μM)	Viability	
	AVE.	± SD.
**0**	100.00	1.75
**0.01**	90.90	1.08
**0.1**	85.88	2.97
**1**	85.87	1.98
**10**	85.74	2.98
**100**	6.28	0.02

Cytotoxicity assay of **4d**

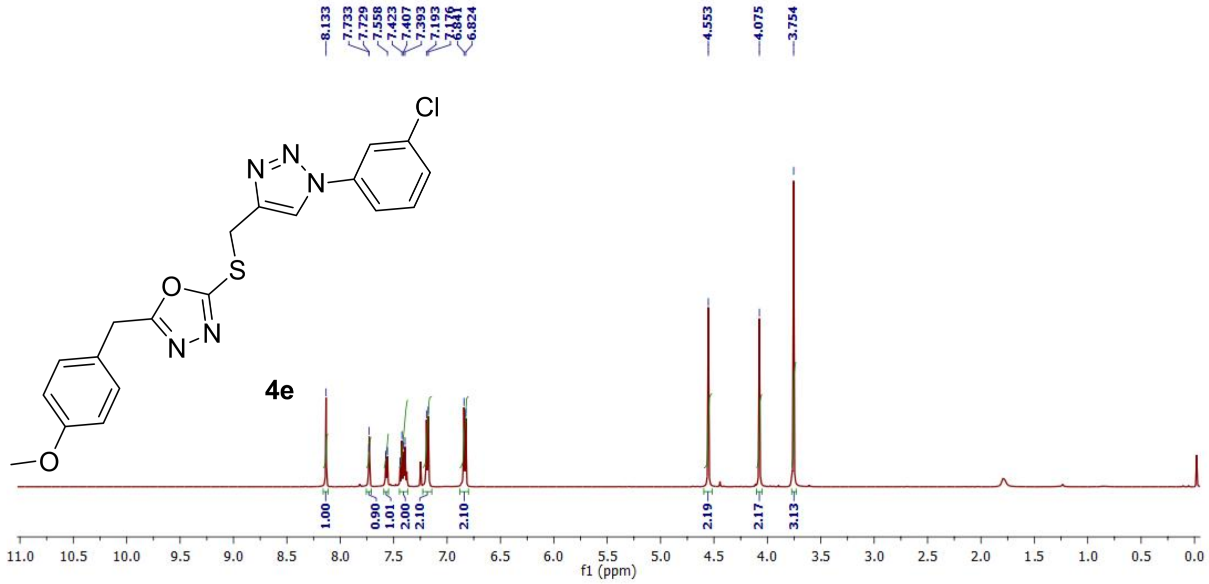

^{1}H NMR of **4e**

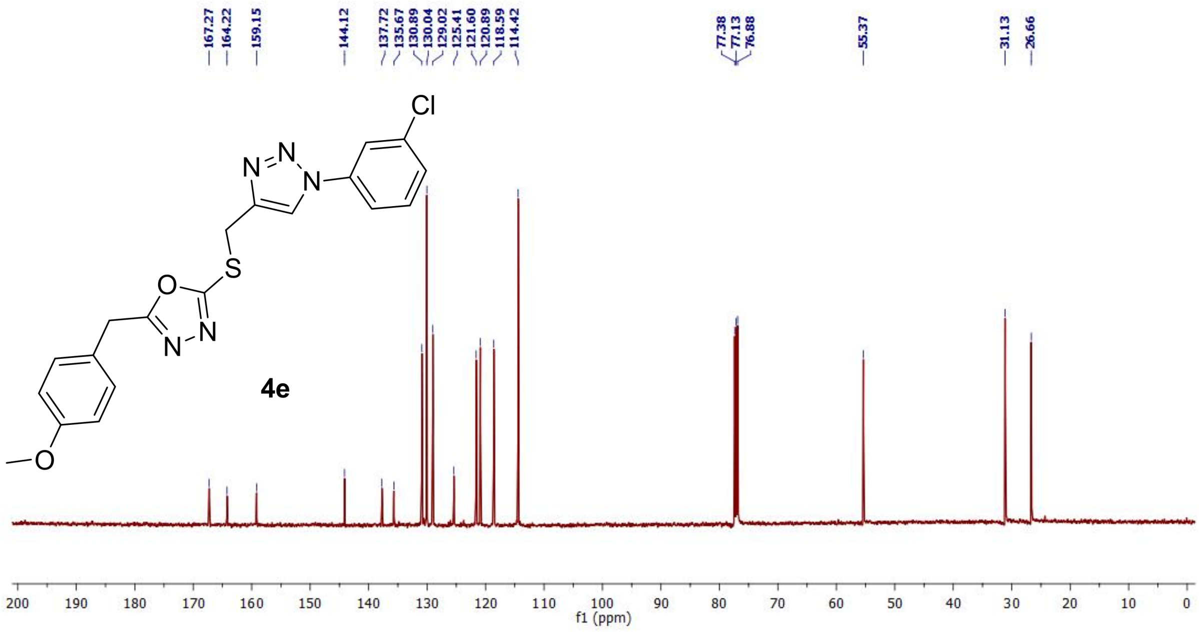
167.27
164.22
159.15
144.12
137.72
135.67
130.89
130.04
129.02
125.41
121.60
120.89
118.59
114.42
77.38
77.13
76.88
55.37
31.13
26.66
Cl
N
N
N
S
O
N
N
O
4e
f1 (ppm)

^{13}C NMR of **4e**

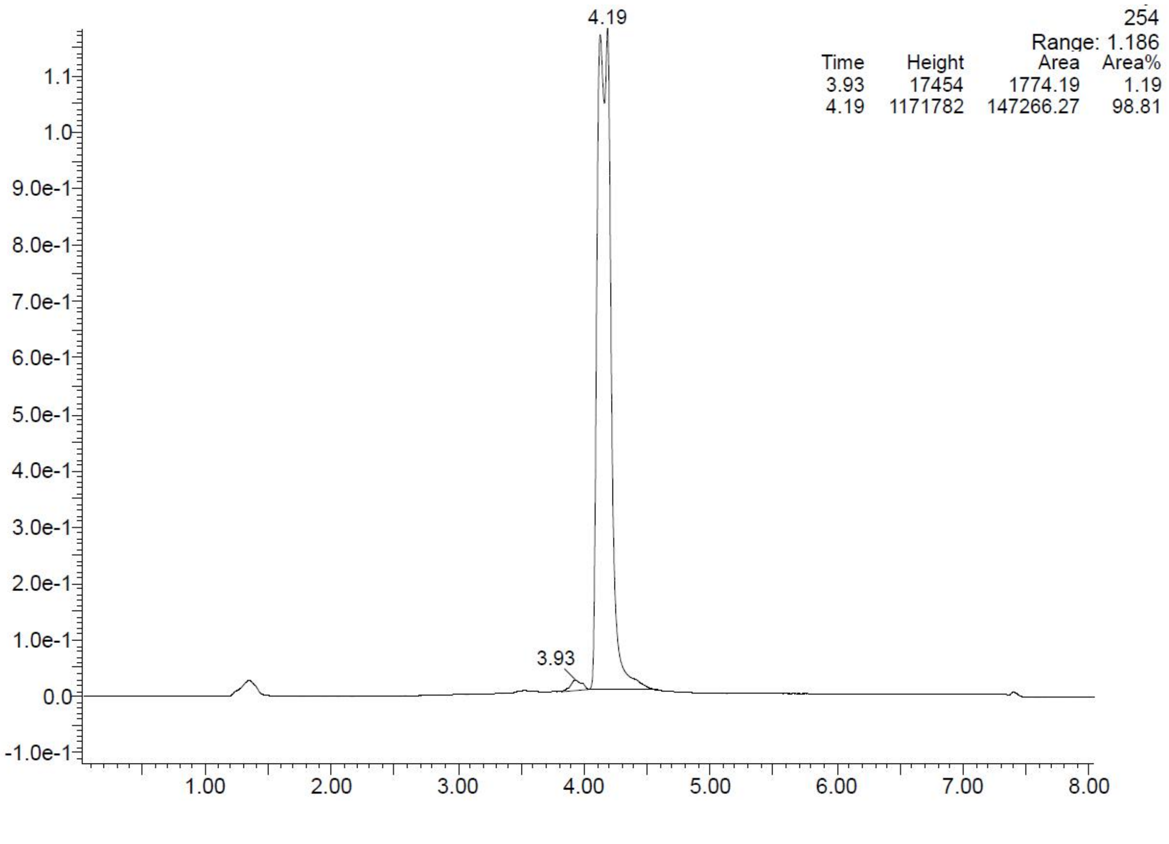
4.19
254
Range: 1.186
Time Height Area Area%
3.93 17454 1774.19 1.19
4.19 1171782 147266.27 98.81
3.93

Liquid chromatogram of

4e

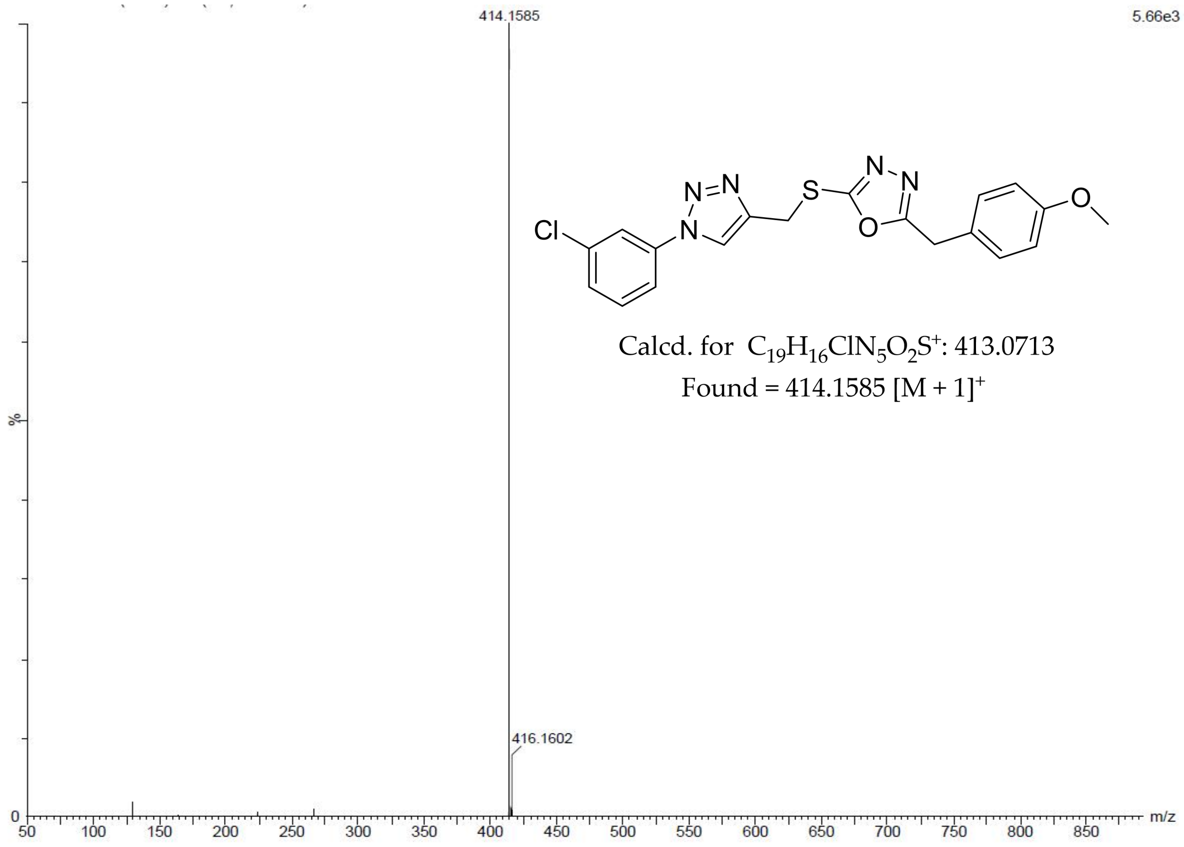

Mass spectra of **4e**

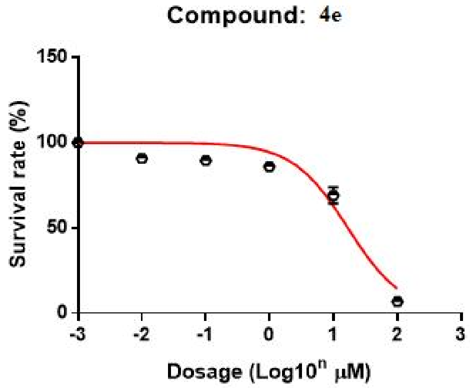

IC_{50} (μM) = 16.86
Log IC_{50} (μM) = 1.23

- **Cell line: MCF7 (2000 cells/per well[96])**
- **Treated time: 72hrs**
- **Assay: alamarBlue (4hrs incubated)**
- **Data: 4e**

Conc. (μM)	Viability	
	AVE.	± SD.
**0**	100.00	1.75
**0.01**	90.74	2.48
**0.1**	89.40	0.28
**1**	85.88	1.21
**10**	69.05	4.71
**100**	6.70	0.07

Cytotoxicity assay of **4e**

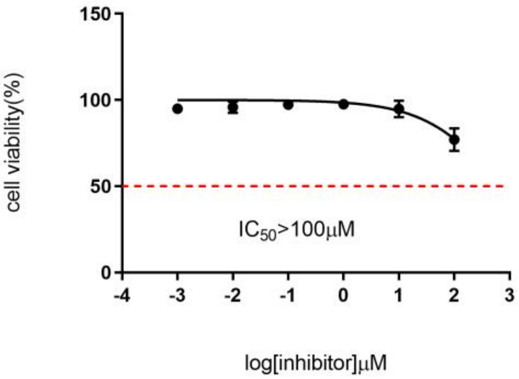

Conc.(μM)	Viability	
	AVE.	±SD
0	100.00	2.76
0.01	95.80	3.21
0.1	97.33	1.15
1	97.50	2.29
10	94.87	4.86
100	77.10	6.59

Cytotoxicity data for the compound 4e

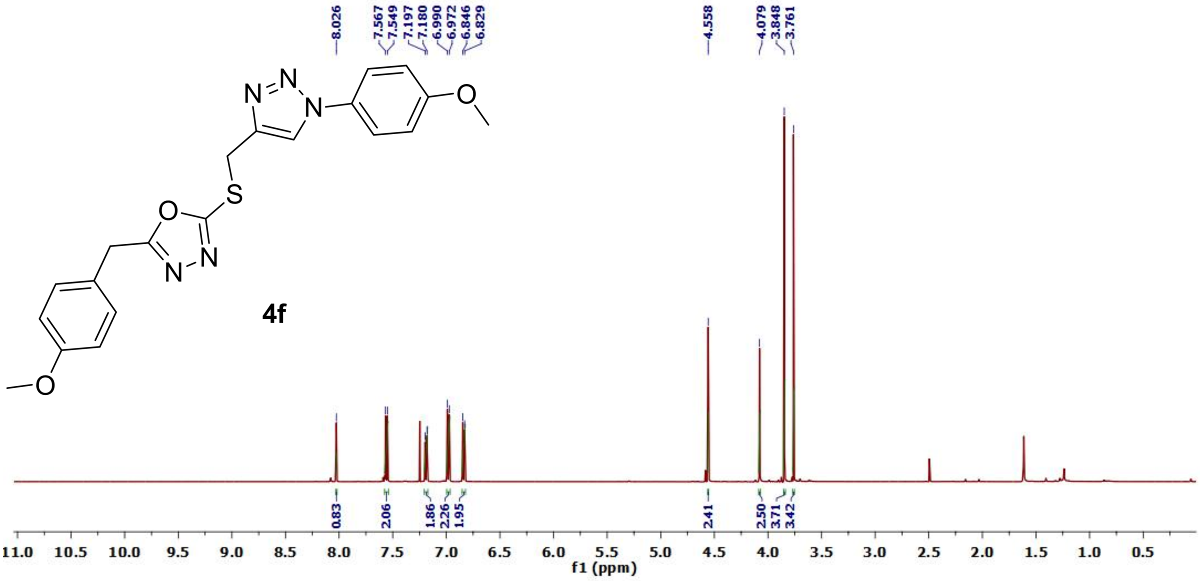

^{1}H NMR of **4f**

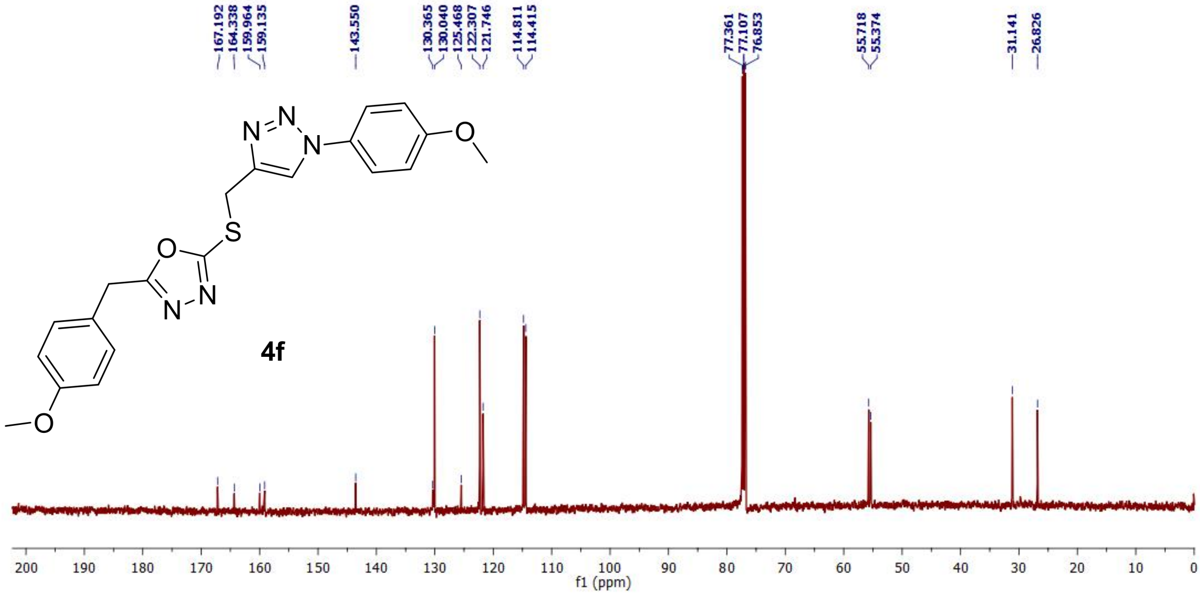

^{13}C NMR of **4f**

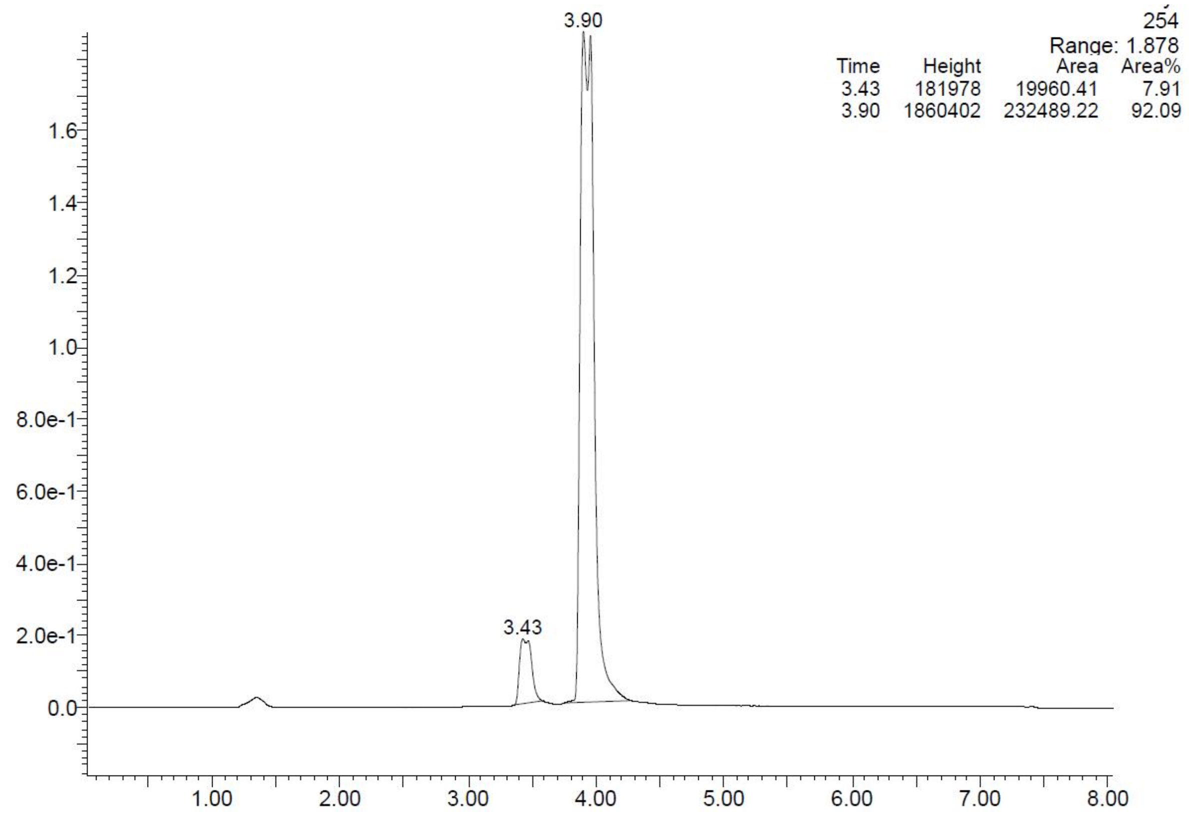

Liquid chromatogram of **4f**

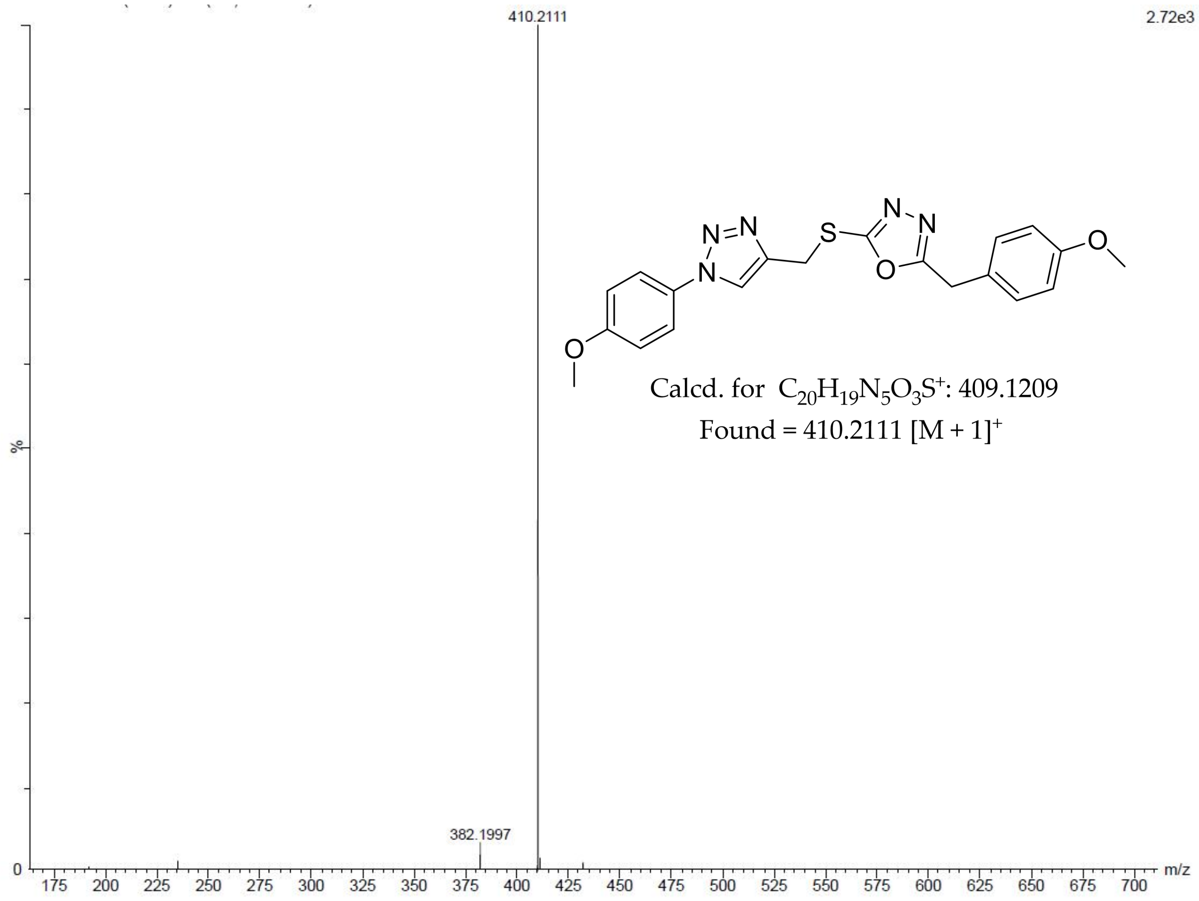

Calcd. for $C_{20}H_{19}N_5O_3S^+$: 409.1209

Found = 410.2111 $[M + 1]^+$

Mass spectra of **4f**

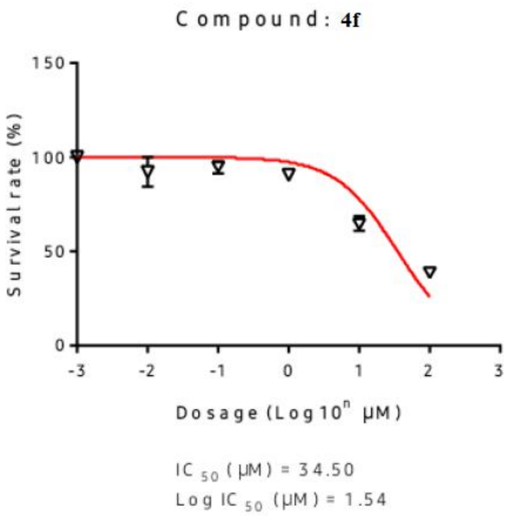

- Cell line: MCF-7 (2000 cells/per well 96-well plate)
- Treated time: 72hrs
- Assay: alamarBlue (4hrs incubated)
- Data: **4f**

Conc. (μM)	Viability	
	AVE.	± SD.
0	100.00	2.45
0.01	91.99	7.77
0.1	94.69	3.12
1	90.48	1.89
10	64.48	4.02
100	38.62	2.45

Cytotoxicity assay of **4f**

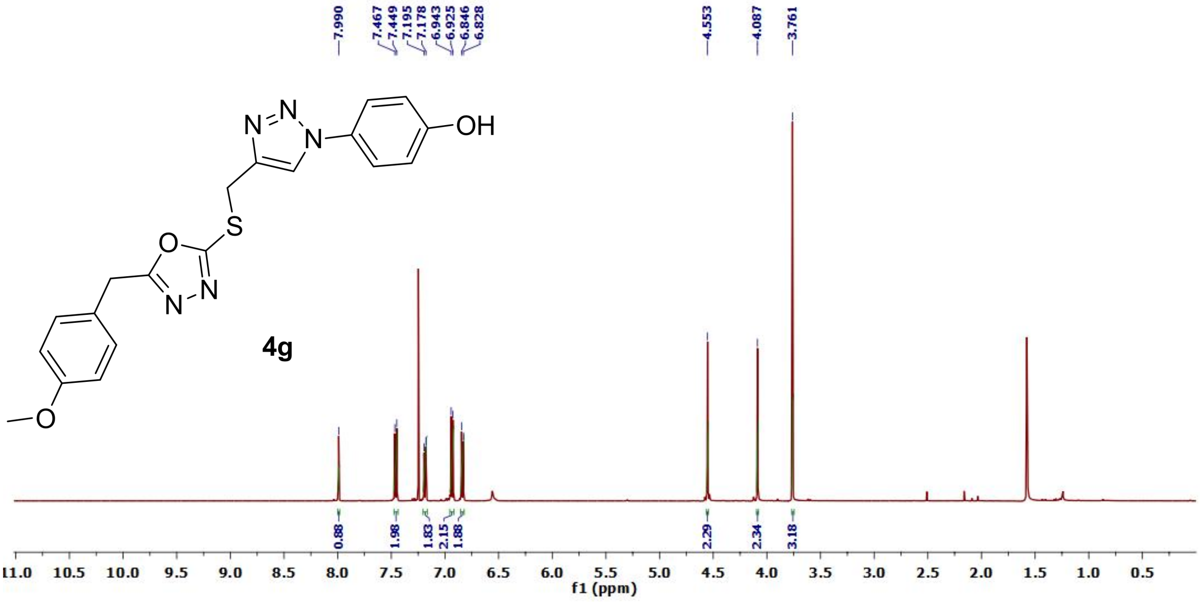

^{1}H NMR of **4g**

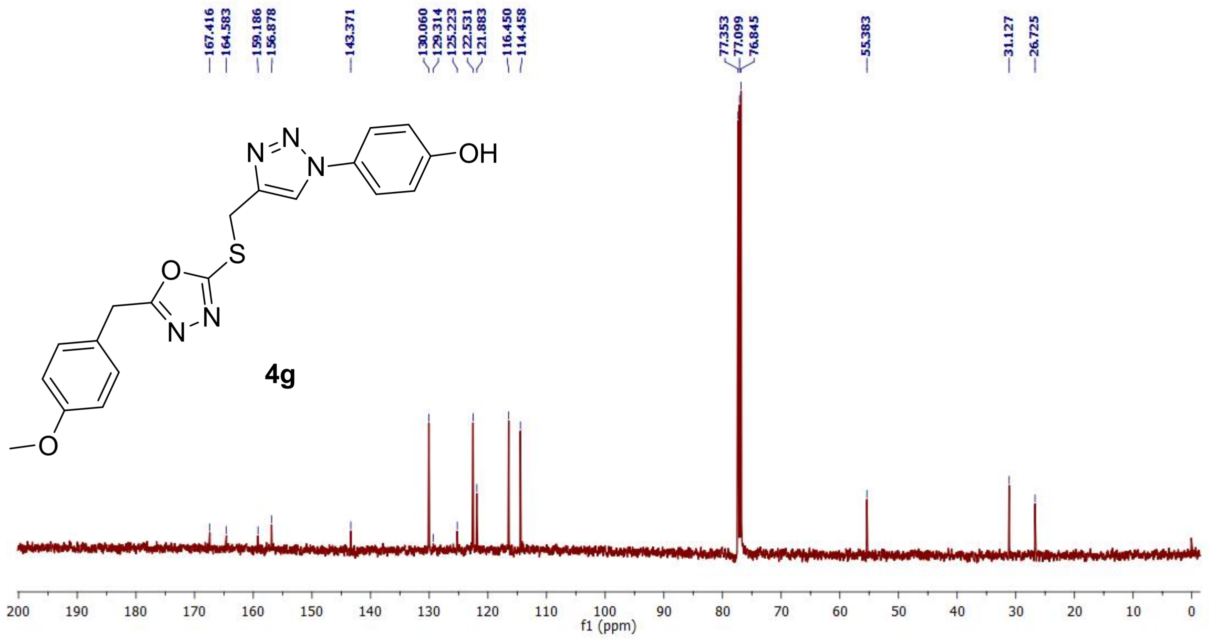

167.416
164.583
159.186
156.878
143.371
130.060
129.314
125.223
122.531
121.883
116.450
114.458
77.353
77.099
76.845
55.383
31.127
26.725
OH
4g
f1 (ppm)

^{13}C NMR of **4g**

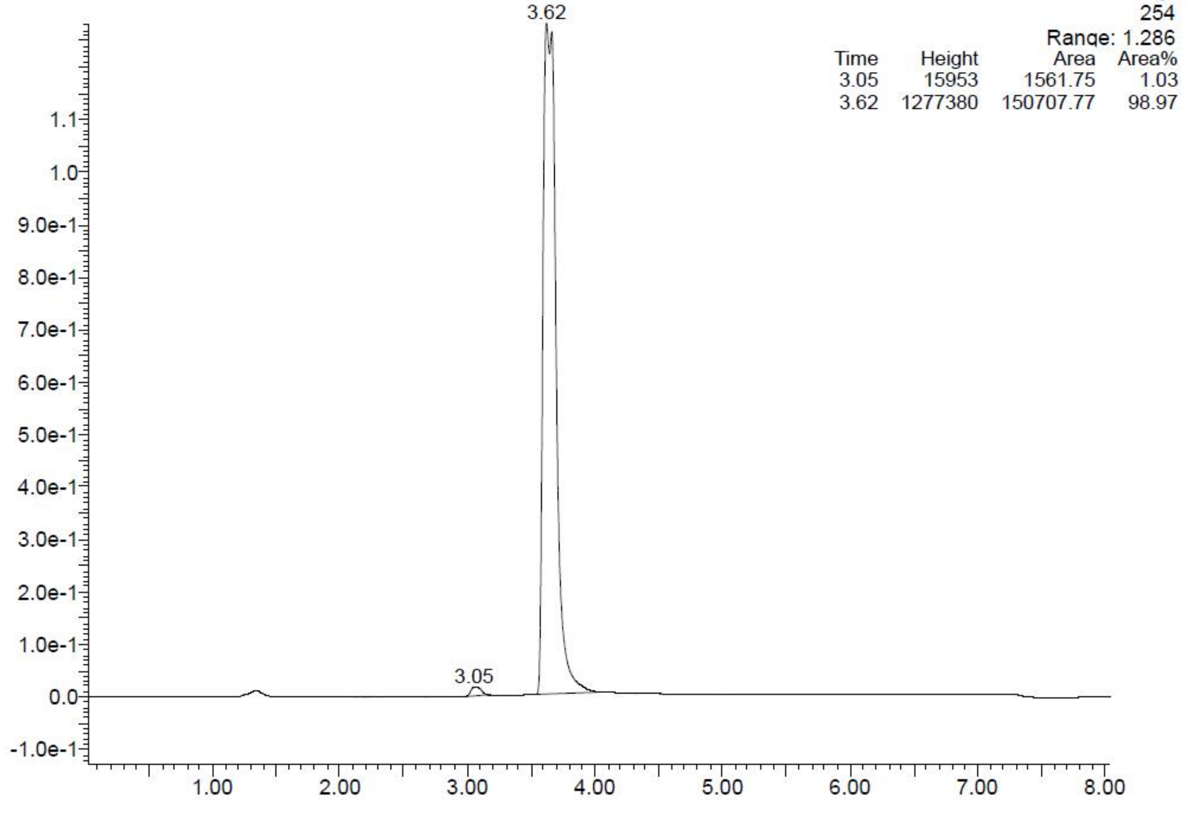

254
Range: 1.286
Time Height Area Area%
3.05 15953 1561.75 1.03
3.62 1277380 150707.77 98.97
3.62
3.05

Liquid chromatogram of **4g**

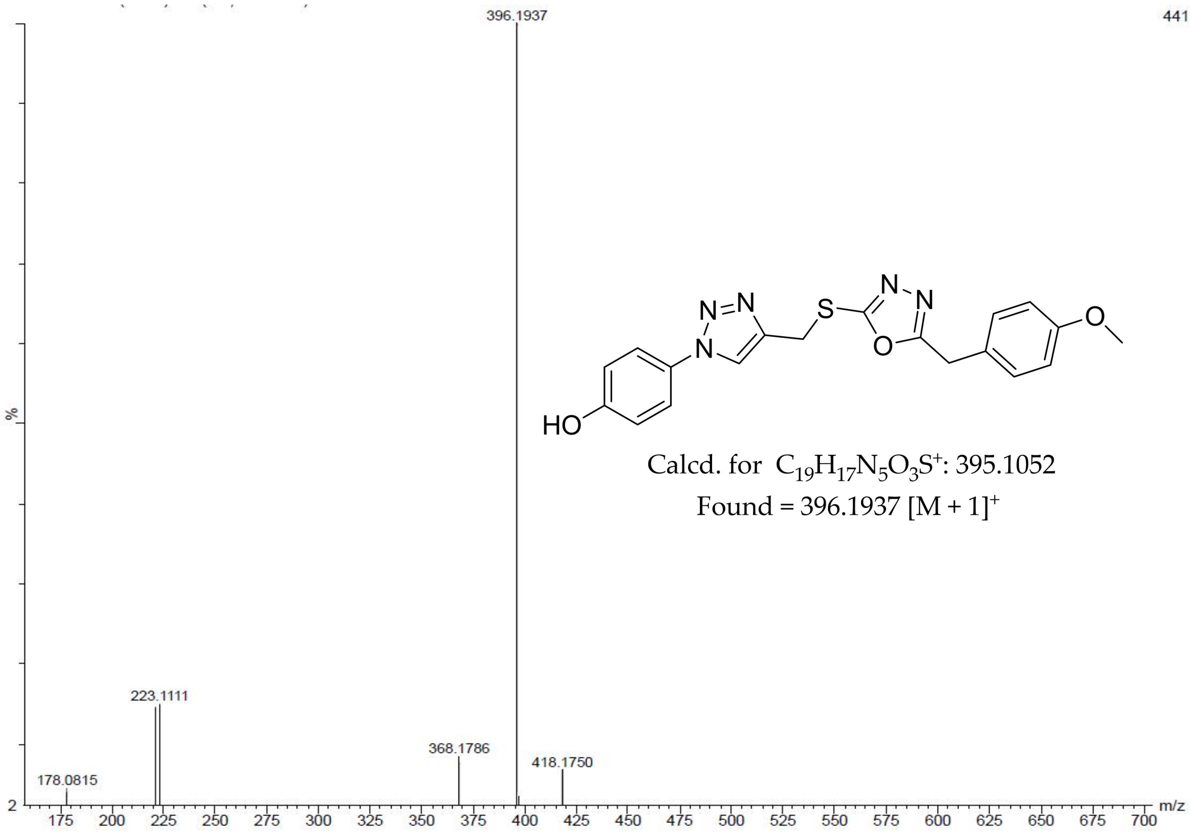

Mass spectra of **4g**

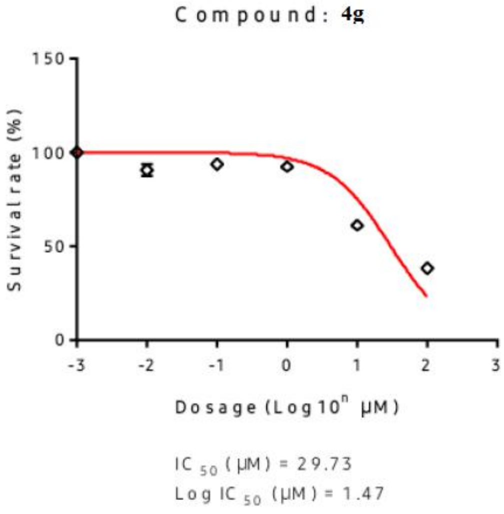

- Cell line: MCF-7 (2000 cells/per well[96-well plate])
- Treated time: 72hrs
- Assay: alamarBlue (4hrs incubated)
- Data: 4g

Conc. (μM)	Viability	
	AVE.	± SD.
0	100.00	2.45
0.01	90.38	3.38
0.1	93.43	0.64
1	92.40	2.29
10	61.24	1.19
100	38.32	1.29

Cytotoxicity assay of **4g**

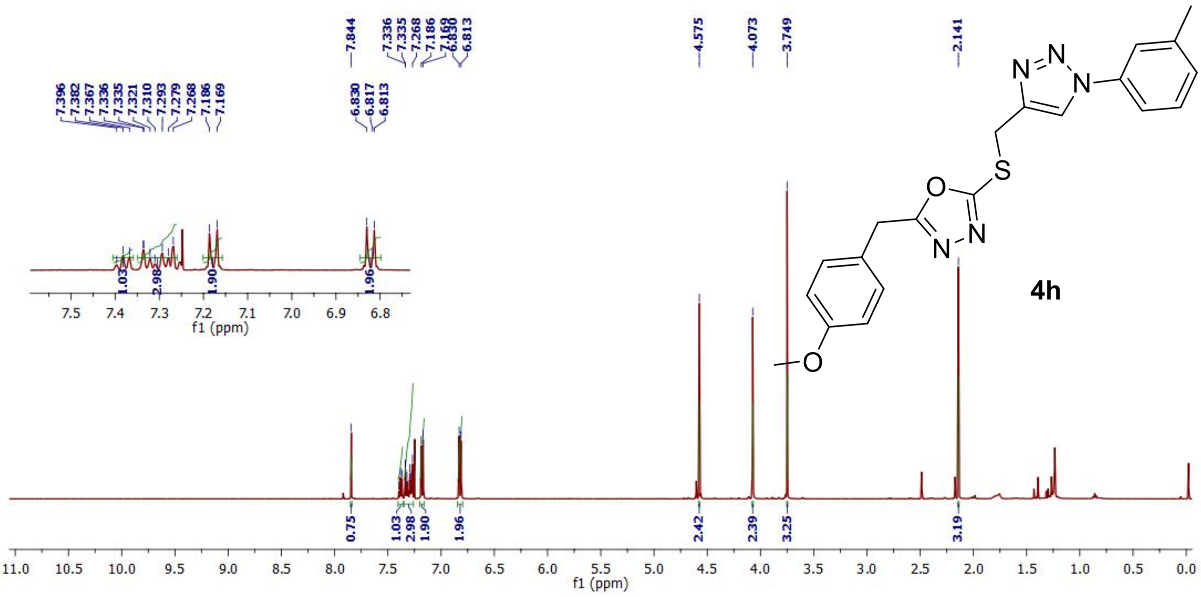

^{1}H NMR of **4h**

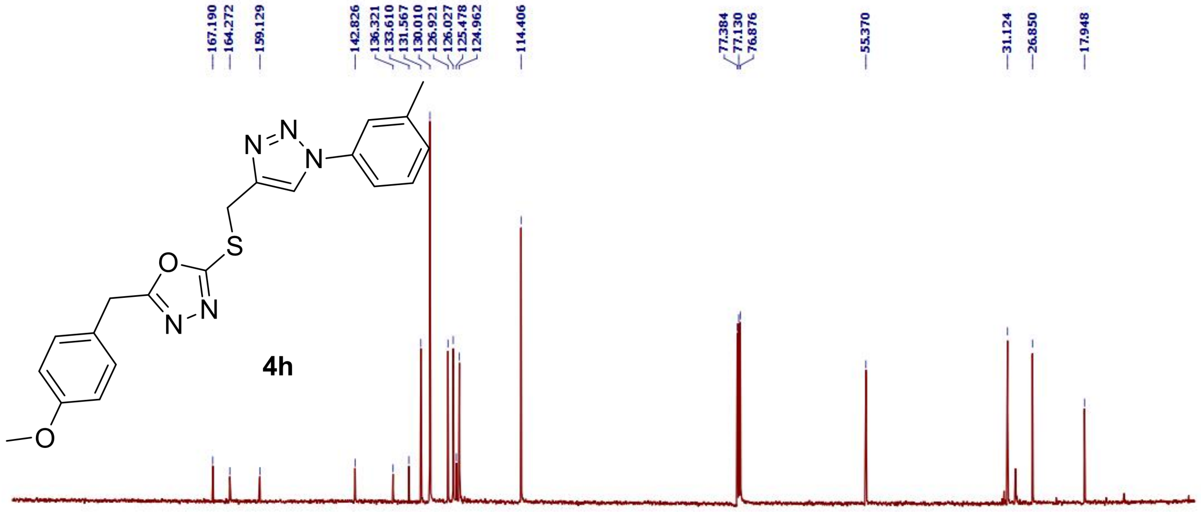

^{13}C NMR of **4h**

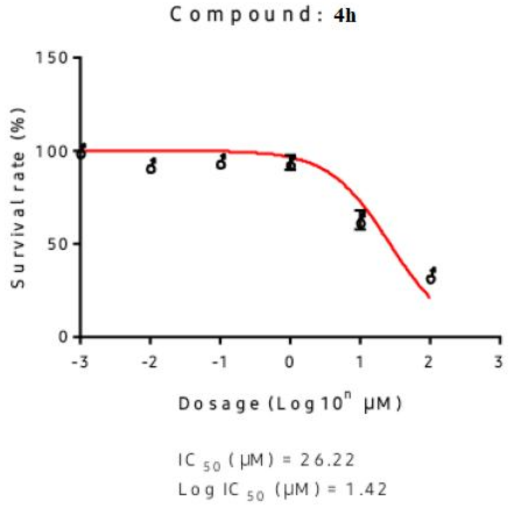

- Cell line: MCF-7 (2000 cells/per well 96-well plate)
- Treated time: 72hrs
- Assay: alamarBlue (4hrs incubated)
- Data: **4h**

Conc. (μM)	Viability	
	AVE.	± SD.
0	100.00	0.89
0.01	92.00	1.86
0.1	94.38	1.70
1	93.81	4.02
10	62.82	4.93
100	33.08	1.38

Cytotoxicity assay of **4h**

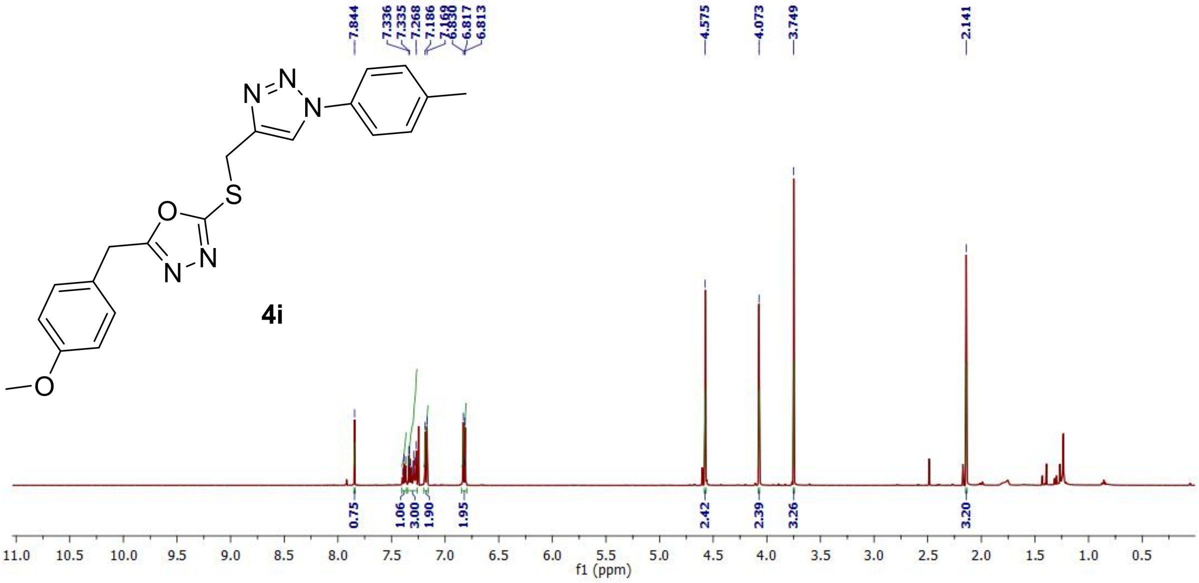

^{1}H NMR of **4i**

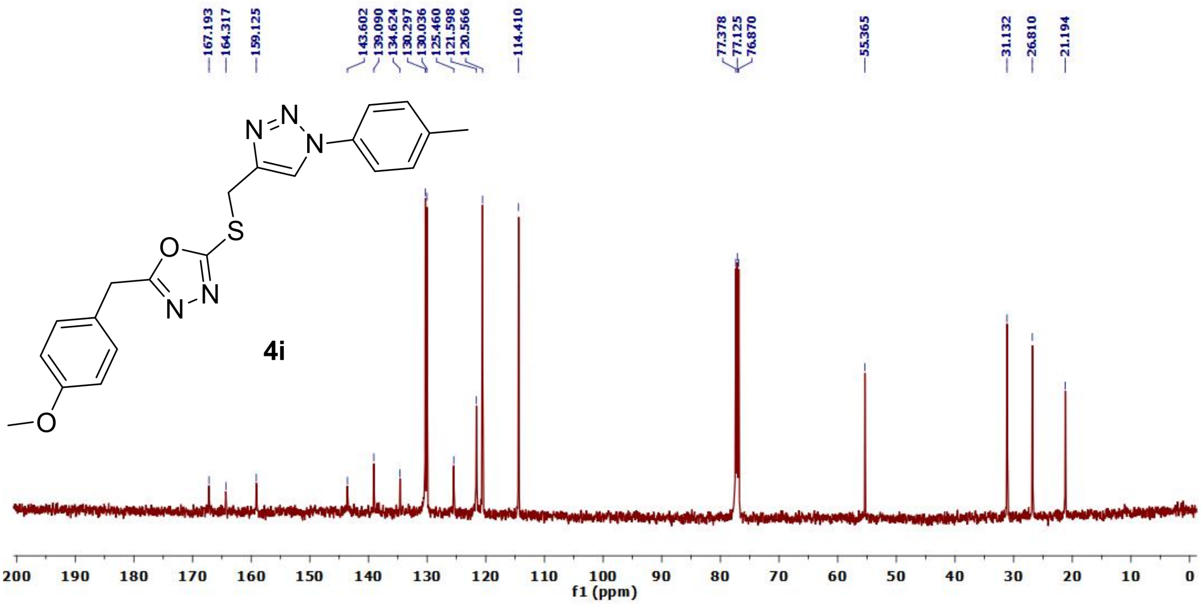

^{13}C NMR of **4i**

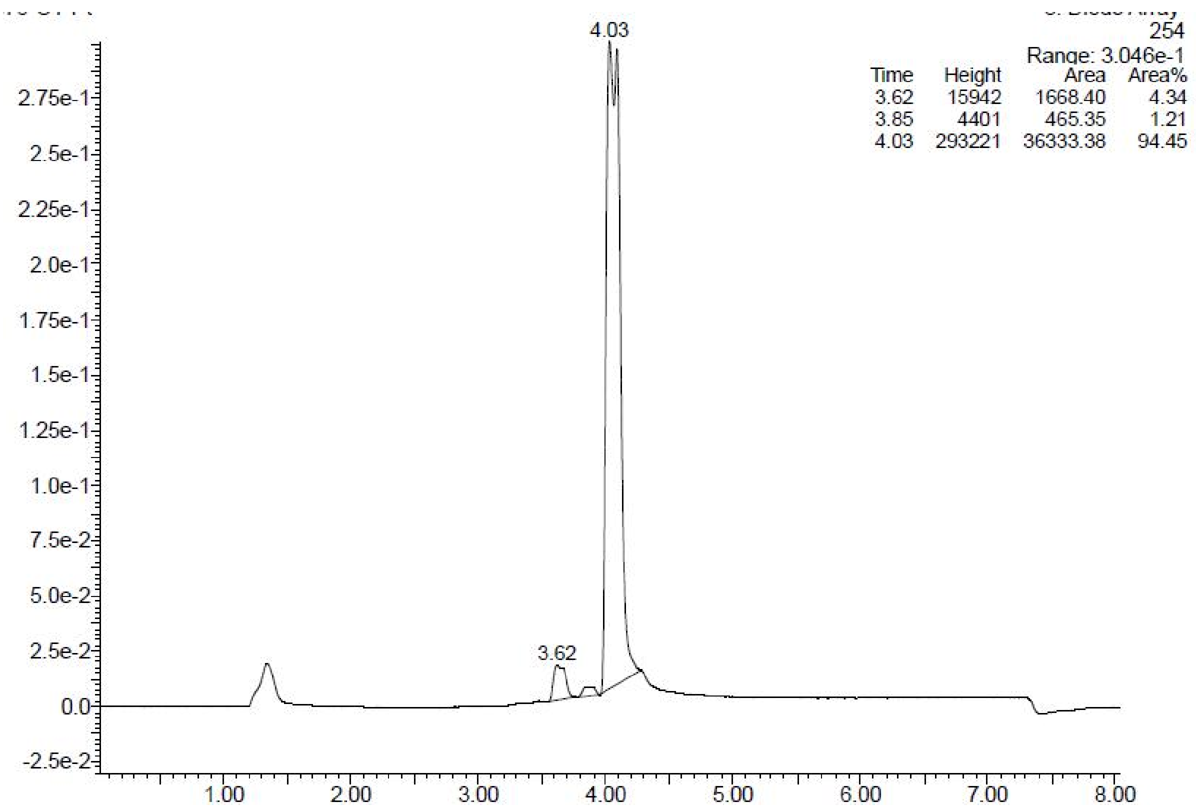

Liquid chromatogram of **4i**

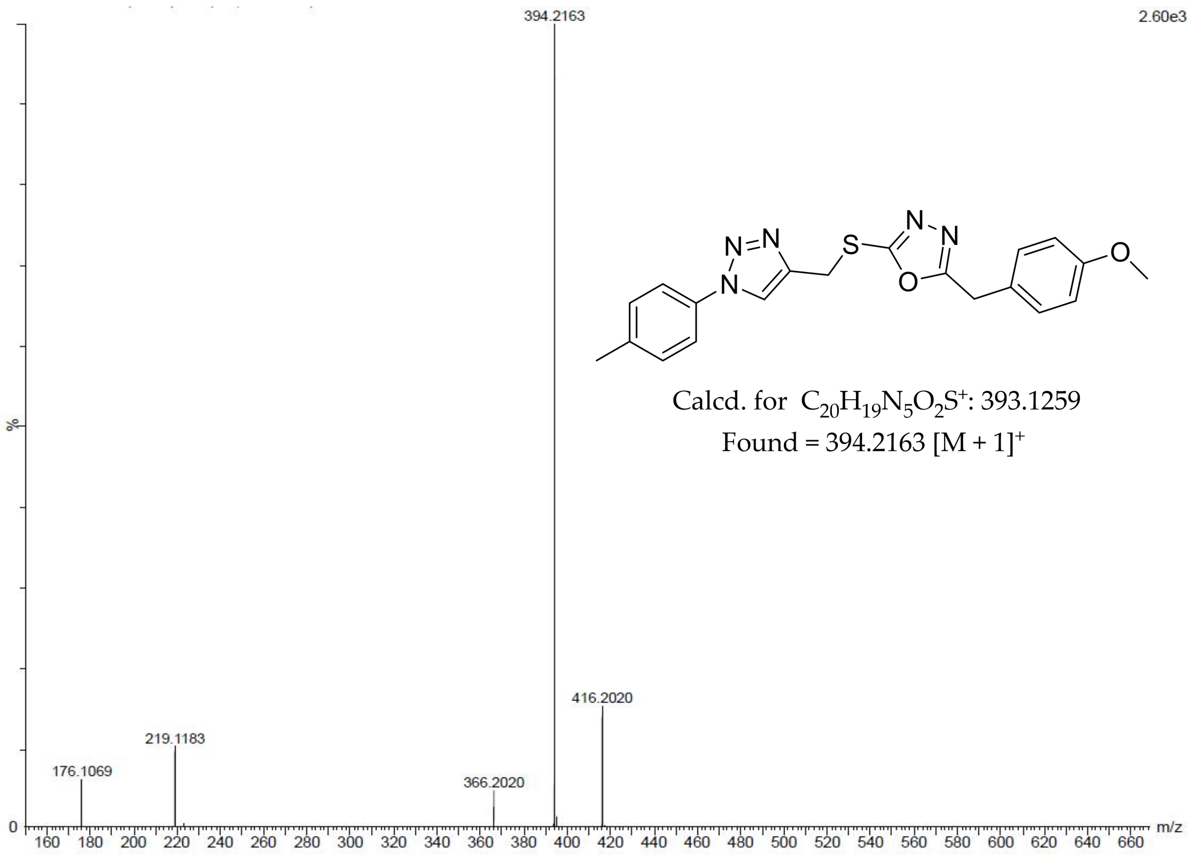

Mass spectra of **4i**

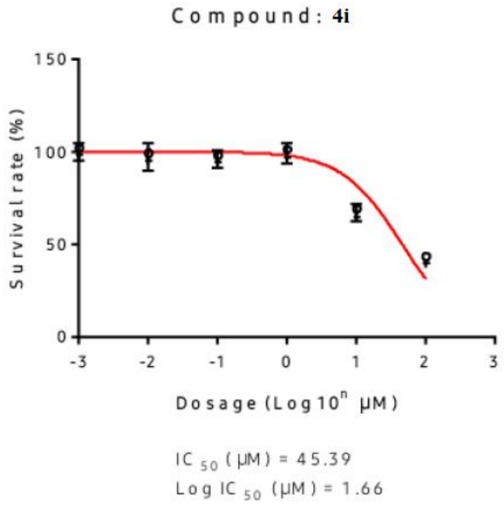

- Cell line: MCF-7 (2000 cells/per well 96-well plate)
- Treated time: 72hrs
- Assay: alamarBlue (4hrs incubated)
- Data: **4i**

Conc. (μM)	Viability	
	AVE.	± SD.
0	100.00	4.39
0.01	97.05	7.43
0.1	96.06	4.82
1	99.11	5.58
10	67.03	4.60
100	41.41	1.38

Cytotoxicity assay of **4i**

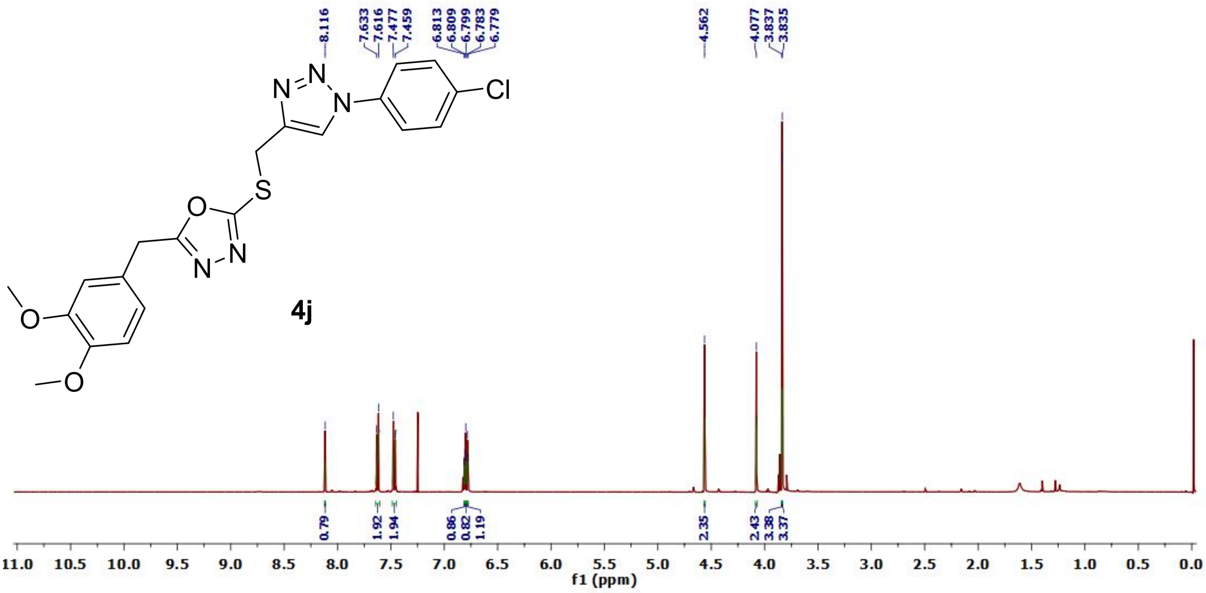

^{1}H NMR of **4j**

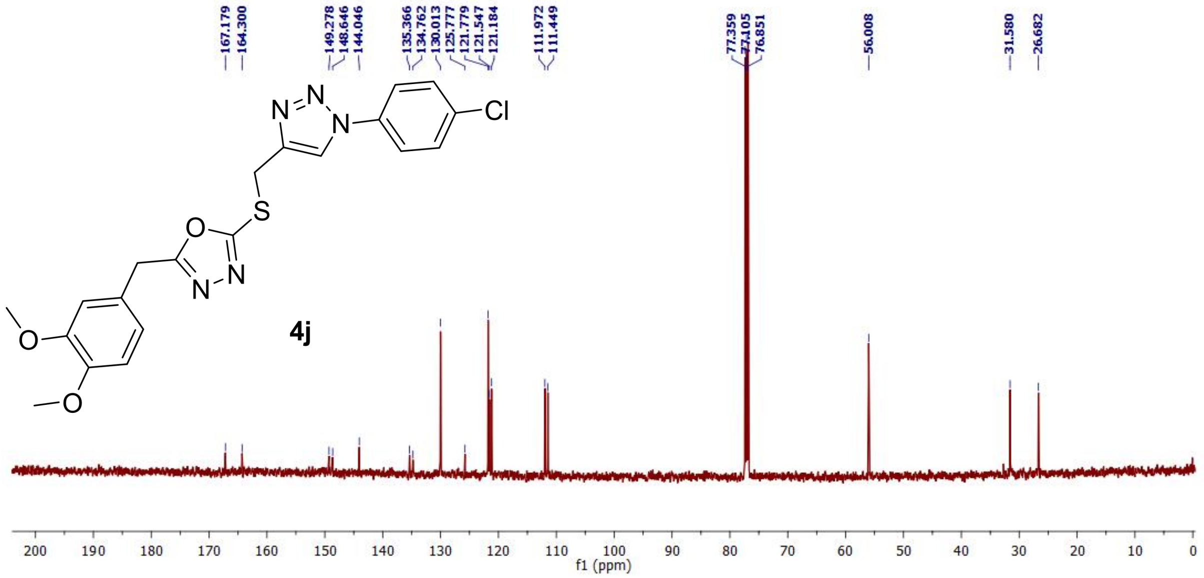

^{13}C NMR of **4j**

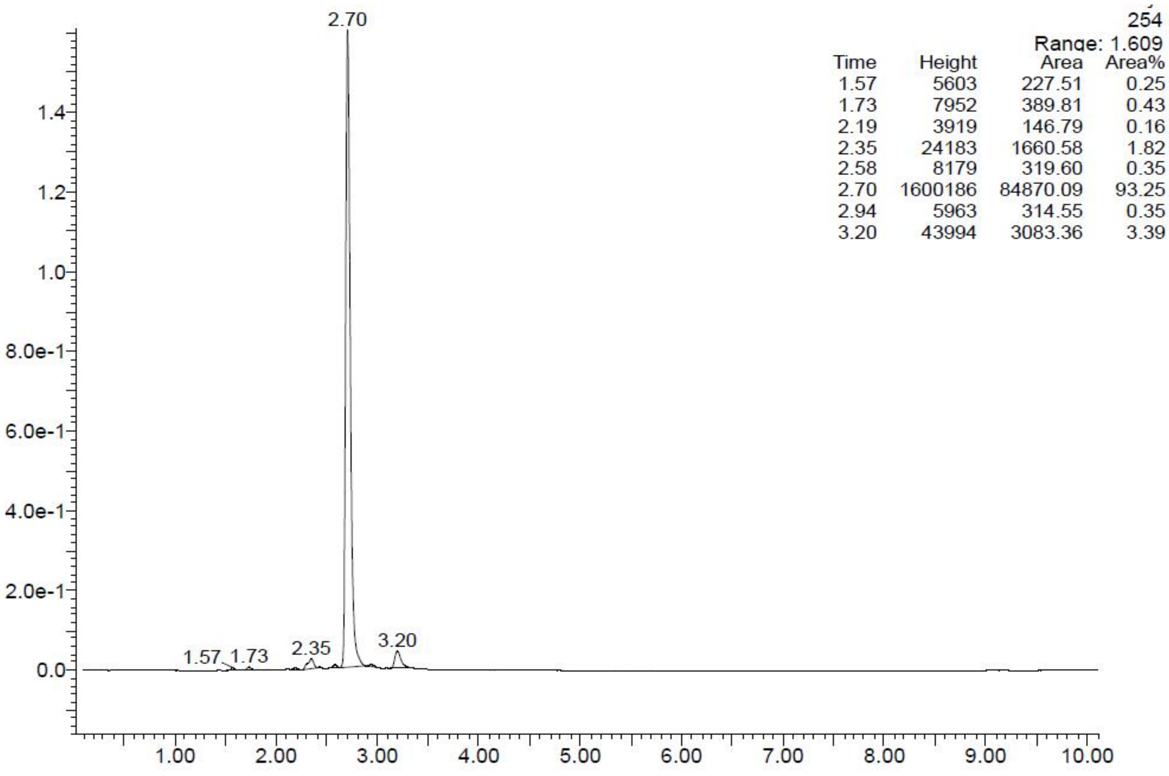

Liquid chromatogram of **4j**

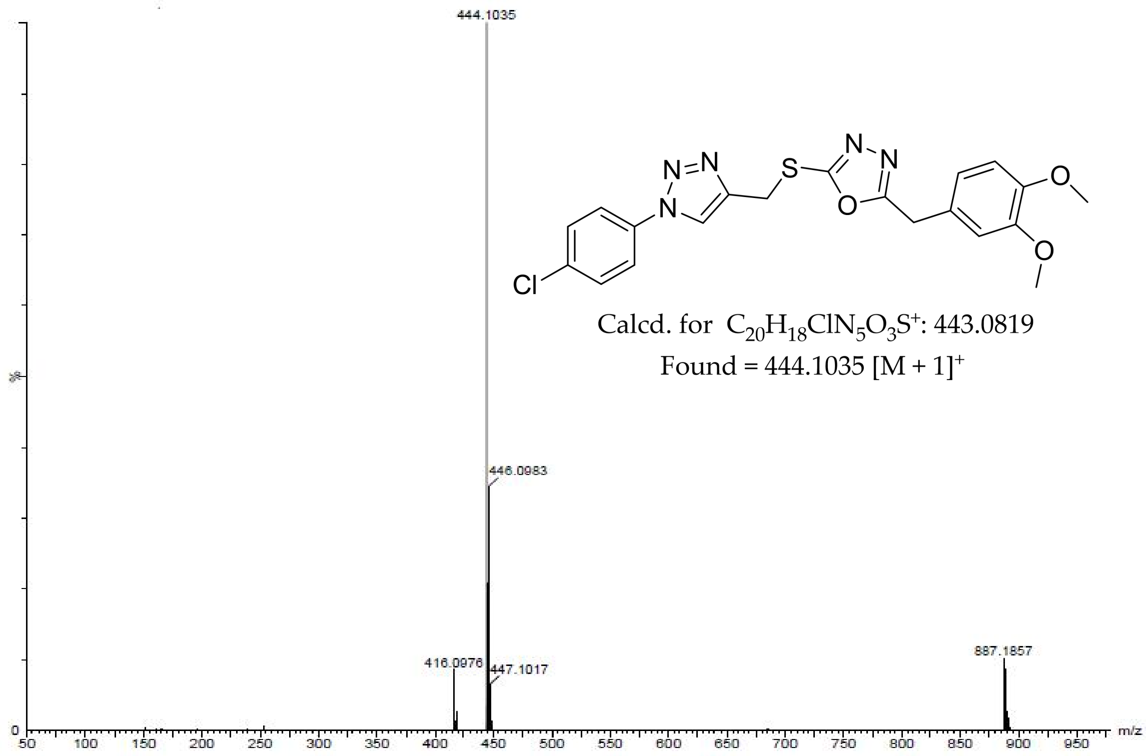

Mass spectra of **4j**

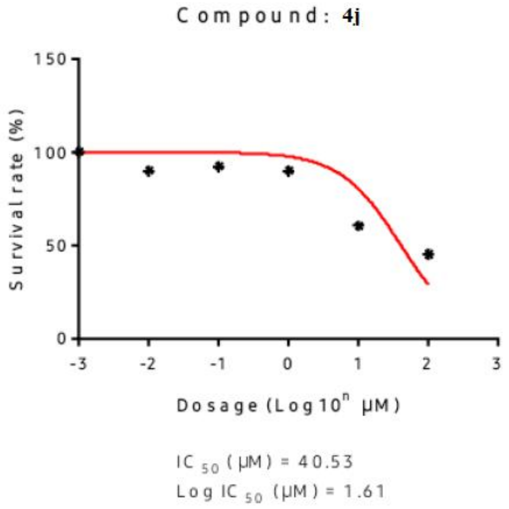

- Cell line: MCF-7 (2000 cells/per well 96-well plate)
- Treated time: 72hrs
- Assay: alamarBlue (4hrs incubated)
- Data: 4j

Conc. (μM)	Viability	
	AVE.	± SD.
0	100.00	2.45
0.01	89.98	2.34
0.1	92.37	1.92
1	89.75	2.73
10	60.40	1.51
100	45.16	0.89

Cytotoxicity assay of **4j**

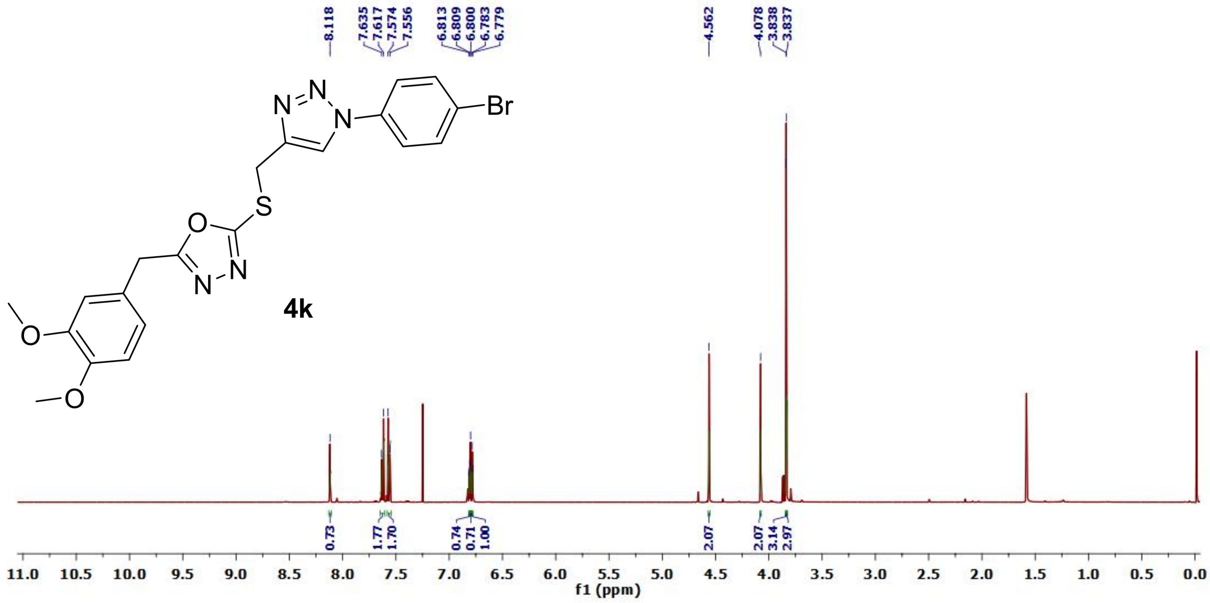

^{1}H NMR of **4k**

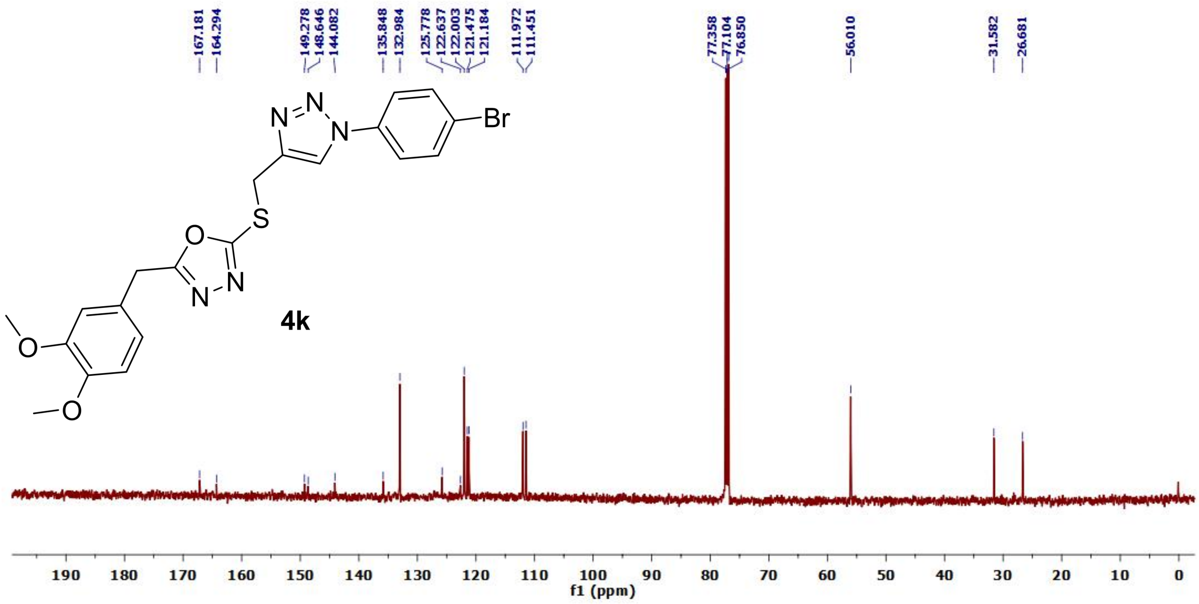

^{13}C NMR of **4k**

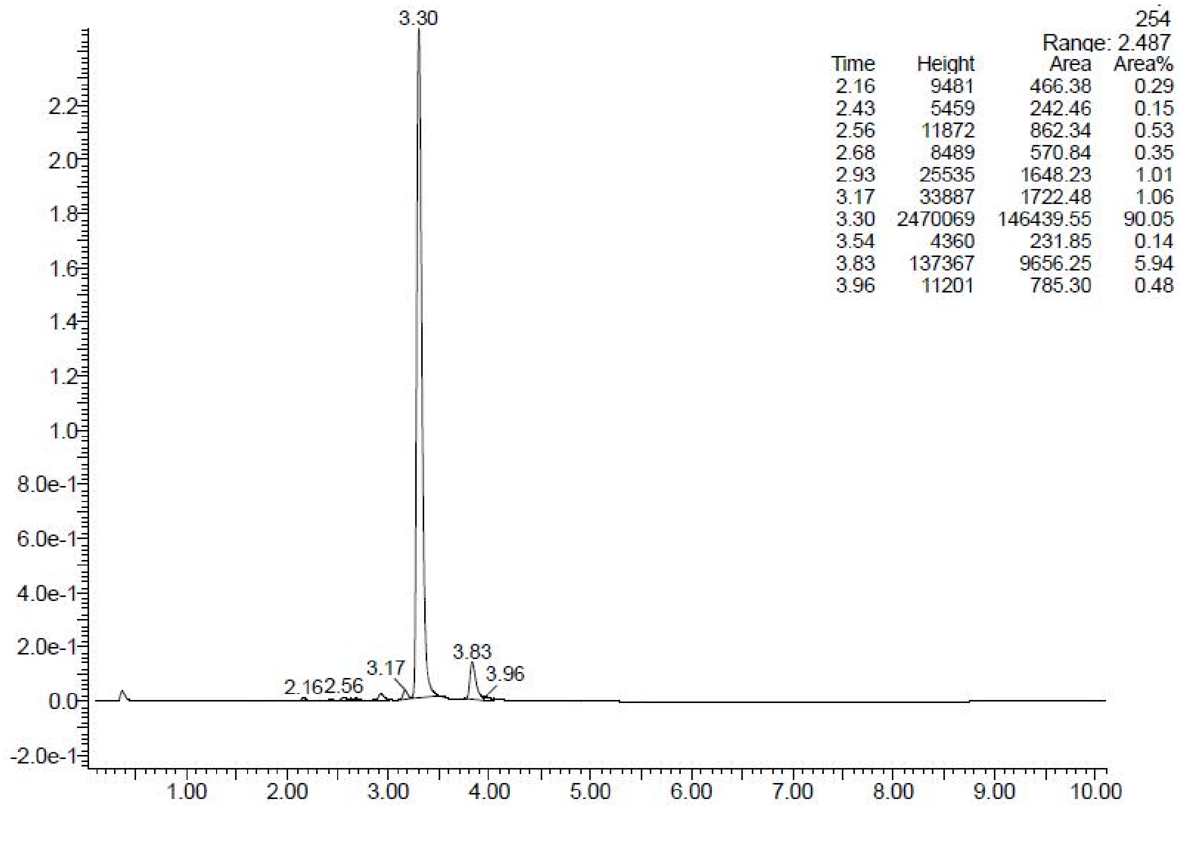

Liquid chromatogram of **4k**

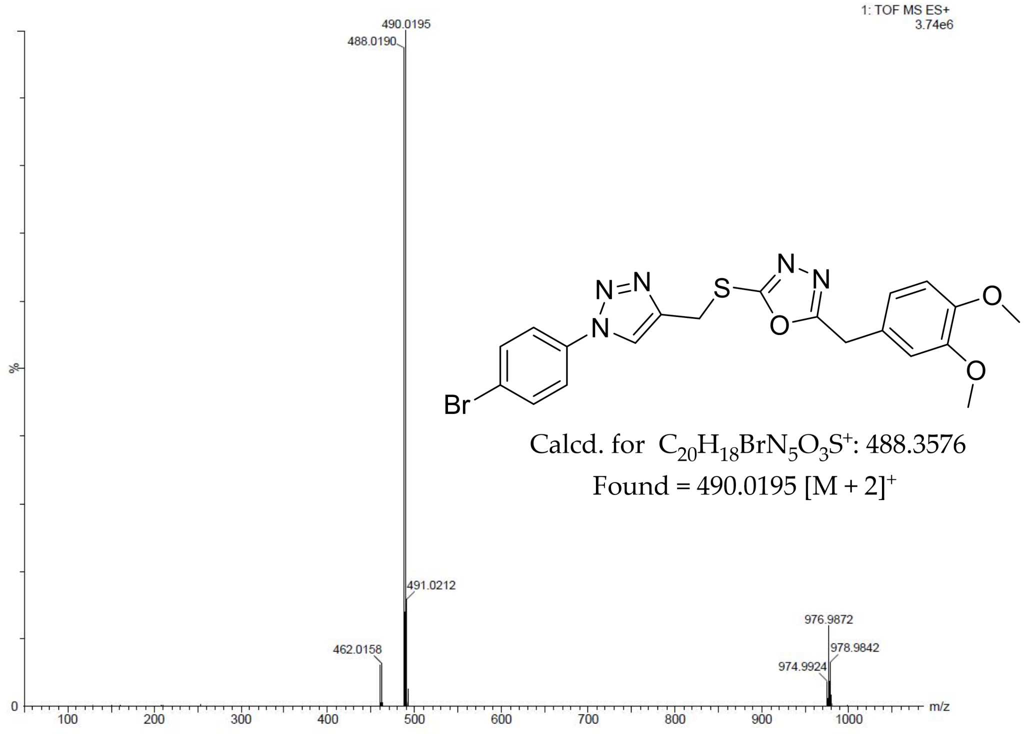

Mass spectra of **4k**

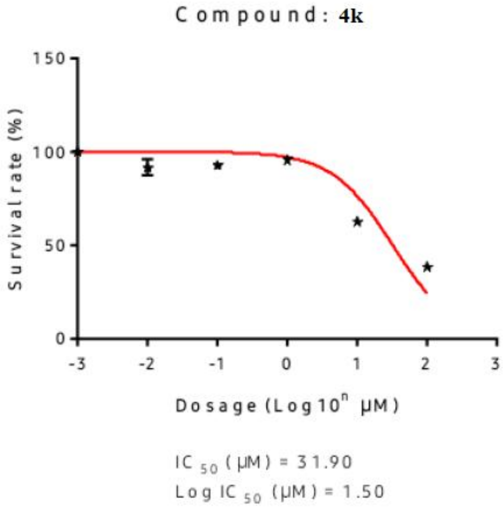

- Cell line: MCF-7 (2000 cells/per well[96-well plate])
- Treated time: 72hrs
- Assay: alamarBlue (4hrs incubated)
- Data: **4k**

Conc. (μM)	Viability	
	AVE.	± SD.
0	100.00	2.45
0.01	91.51	4.45
0.1	92.52	1.81
1	95.54	1.54
10	62.40	2.35
100	38.16	0.24

Cytotoxicity assay of **4k**

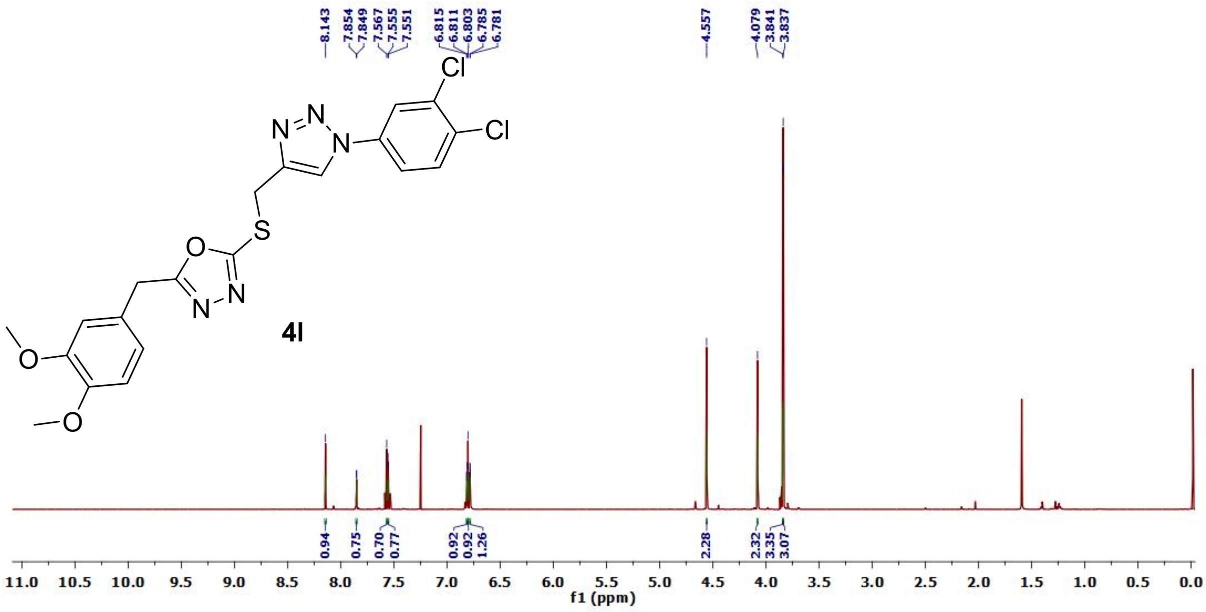

^{1}H NMR of **4l**

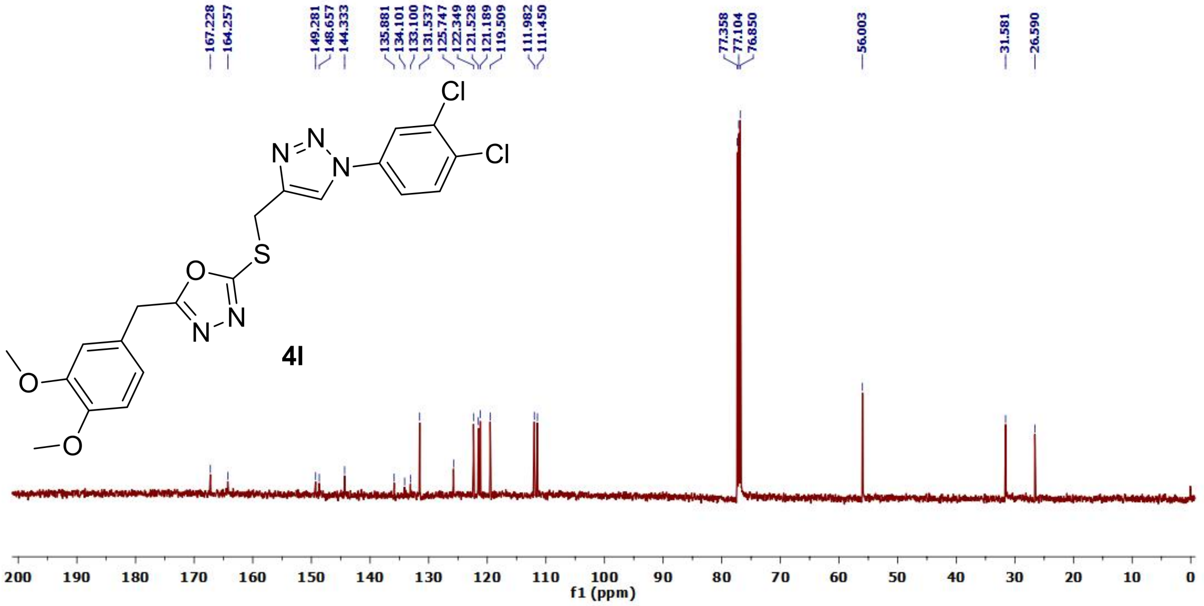

^{13}C NMR of **4l**

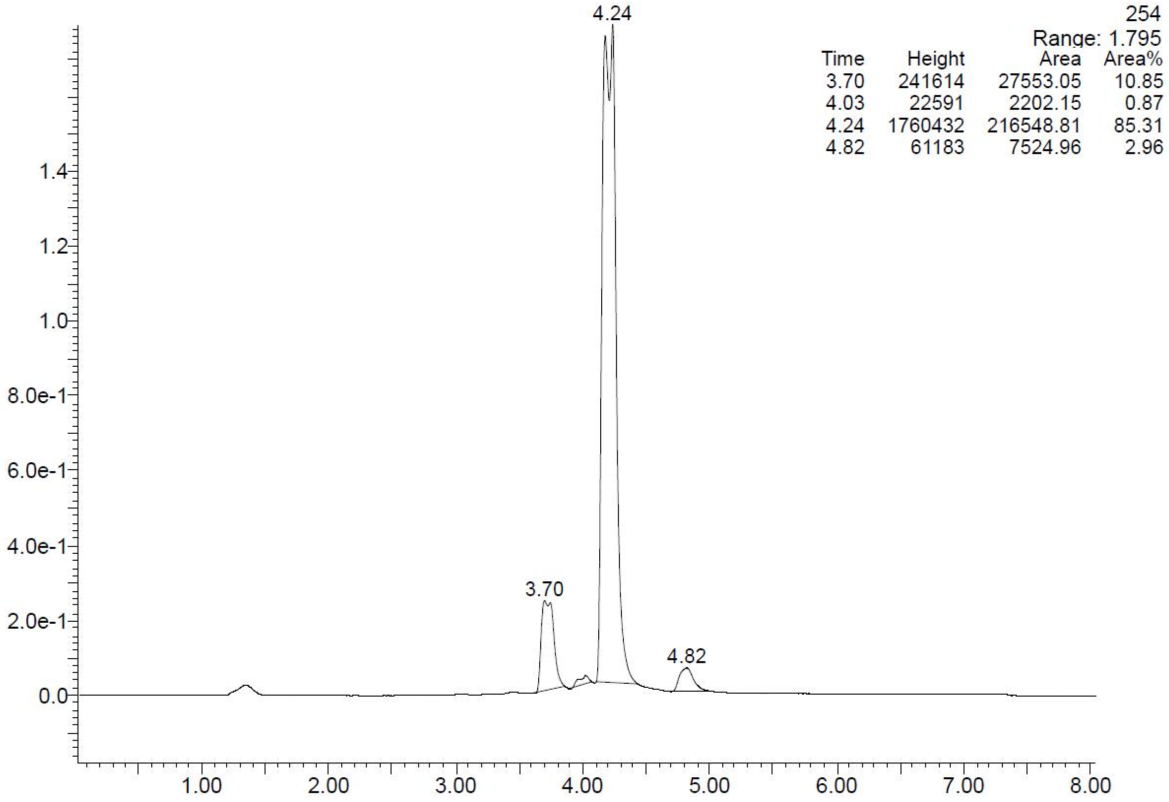

Liquid chromatogram of **4l**

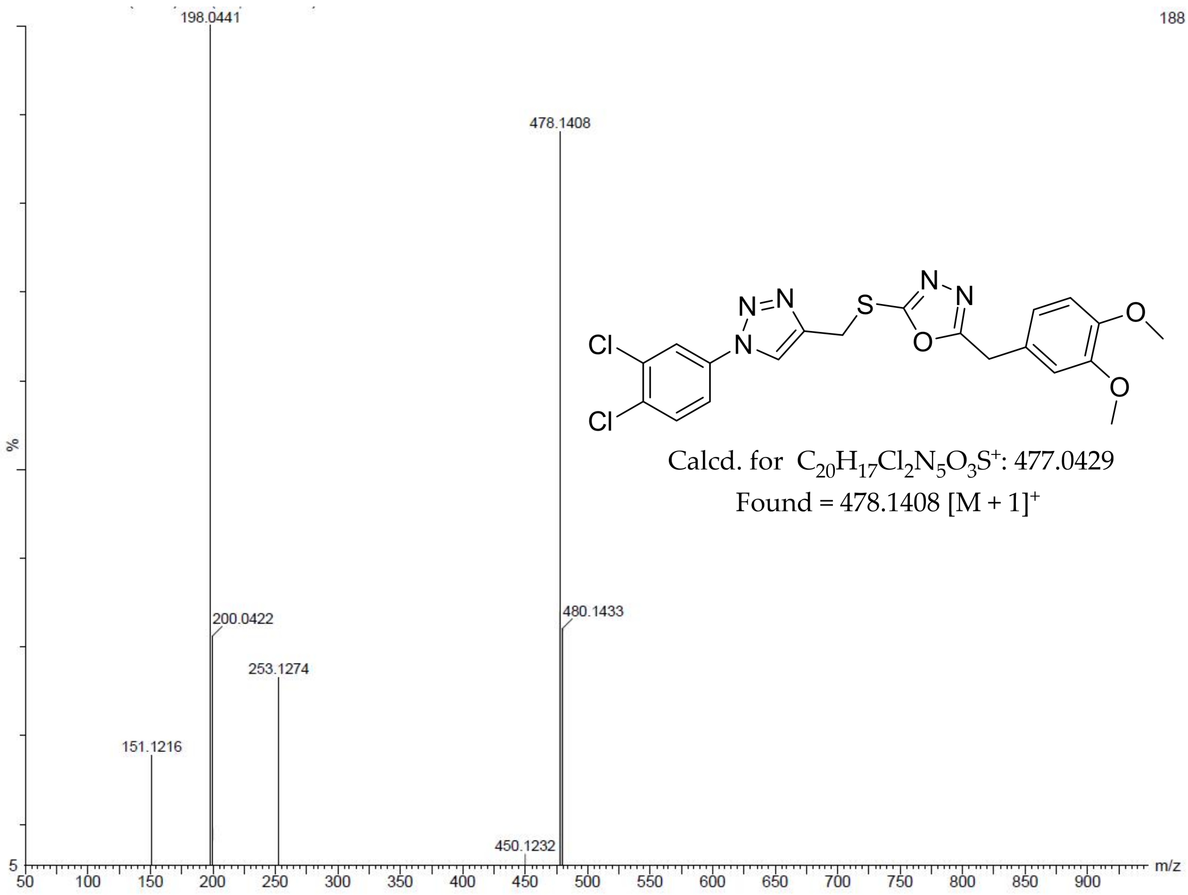

Mass spectra of **4l**

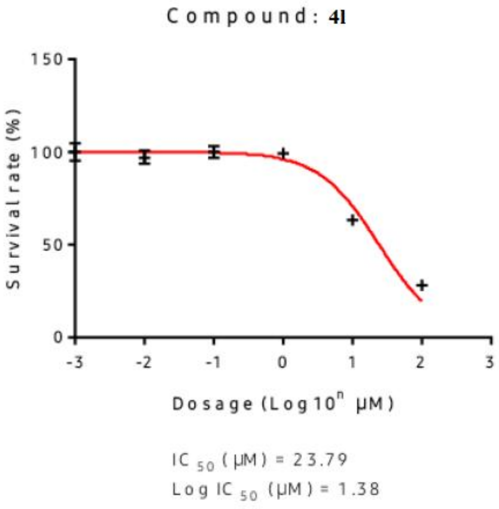

- Cell line: MCF-7 (2000 cells/per well[96-well plate])
- Treated time: 72hrs
- Assay: alamarBlue (4hrs incubated)
- Data: 4l

Conc. (μM)	Viability	
	AVE.	± SD.
0	100.00	4.88
0.01	97.10	3.69
0.1	99.81	3.32
1	98.95	2.06
10	63.26	2.05
100	28.07	0.86

Cytotoxicity assay of **4l**

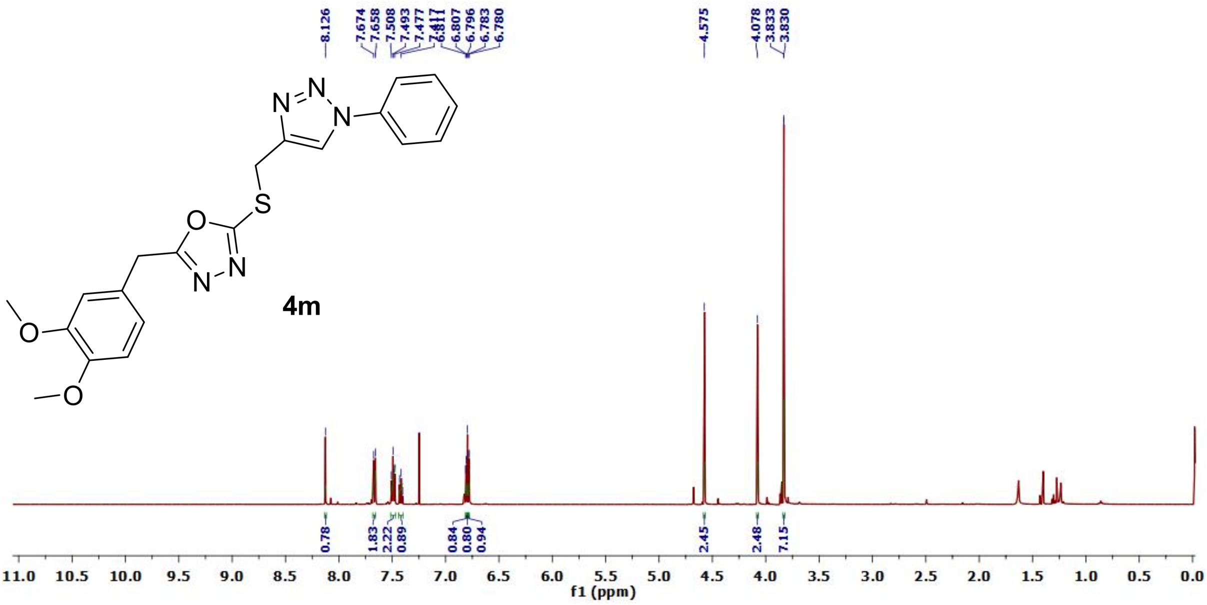

^{1}H NMR of **4m**

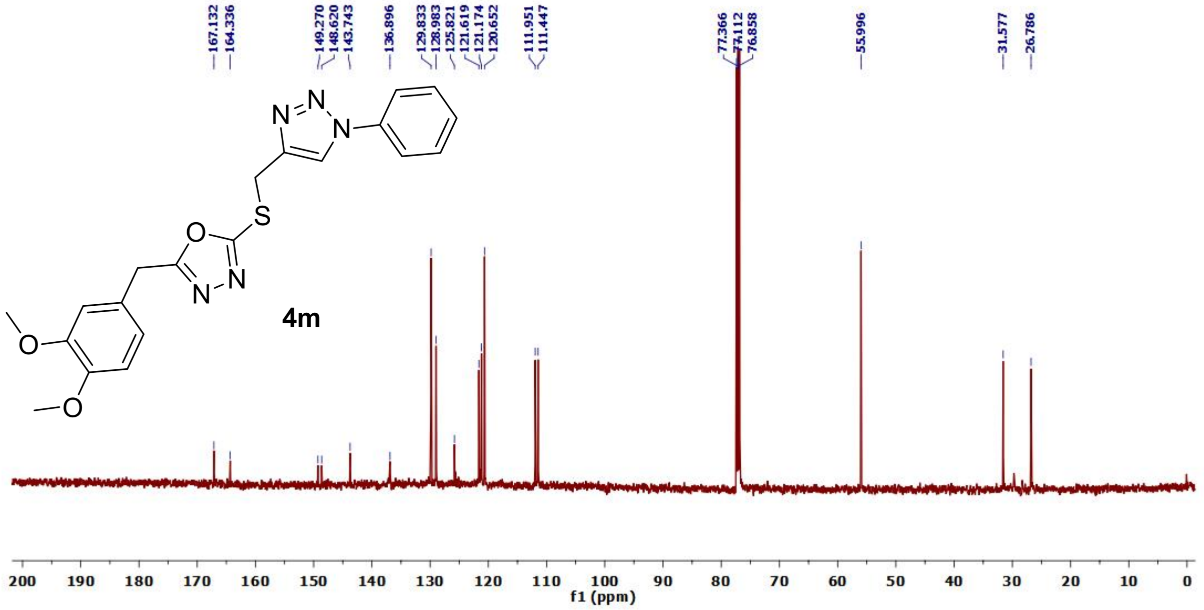

^{13}C NMR of **4m**

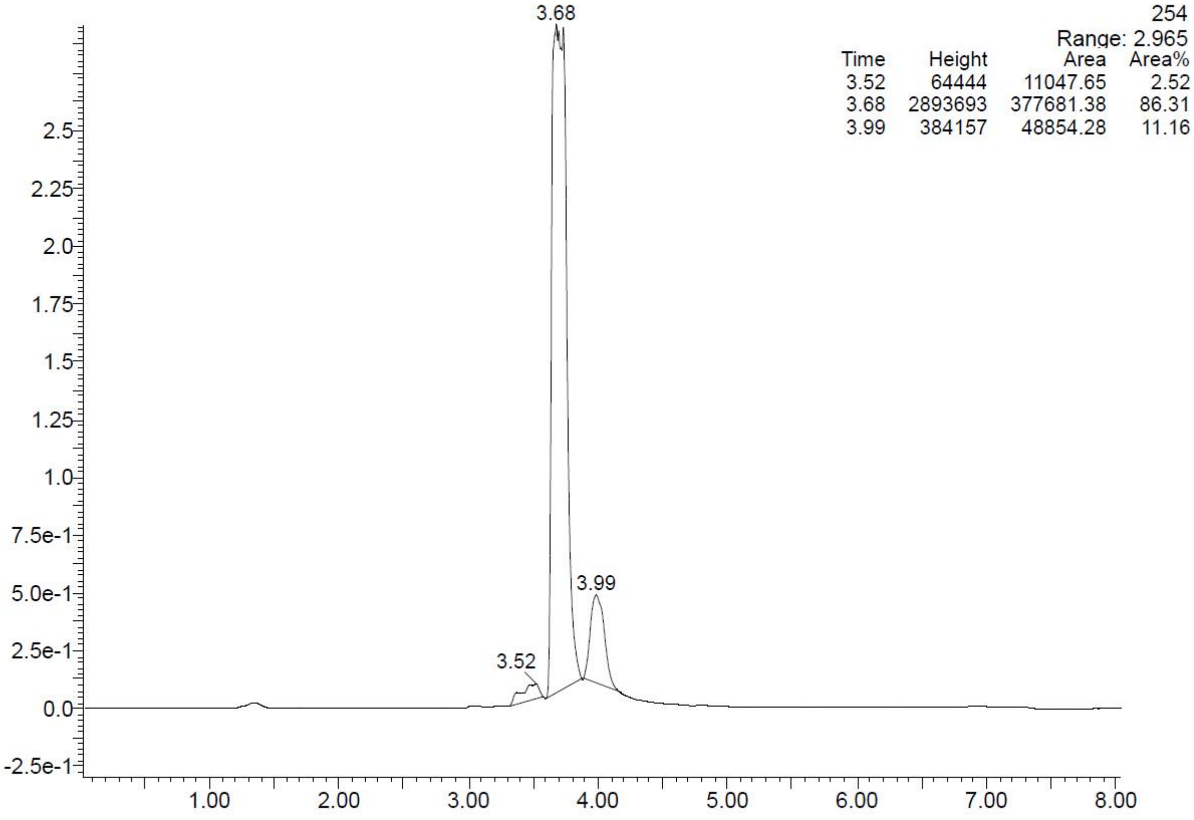

Liquid chromatogram of **4m**

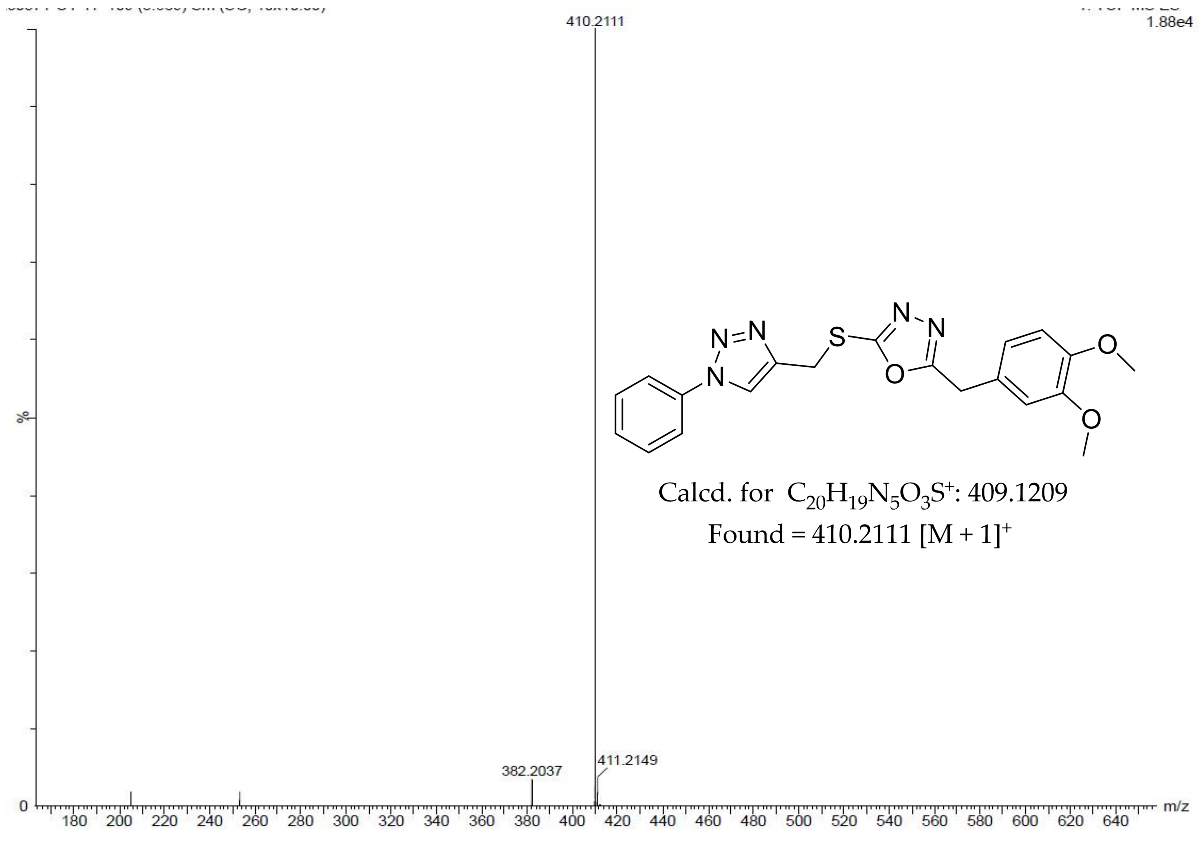

Mass spectra of **4m**

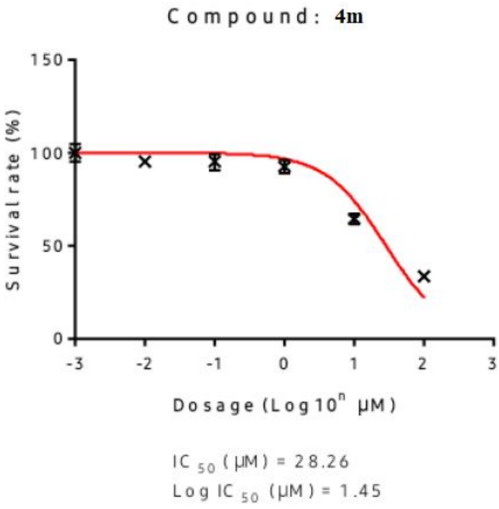

- Cell line: MCF-7 (2000 cells/per well$^{96\text{-well plate}}$)
- Treated time: 72hrs
- Assay: alamarBlue (4hrs incubated)
- Data: **4m**

Conc. (μM)	Viability	
	AVE.	± SD.
0	100.00	4.88
0.01	95.48	1.53
0.1	94.92	4.31
1	92.58	3.55
10	64.30	2.96
100	33.66	0.82

Cytotoxicity assay of **4m**

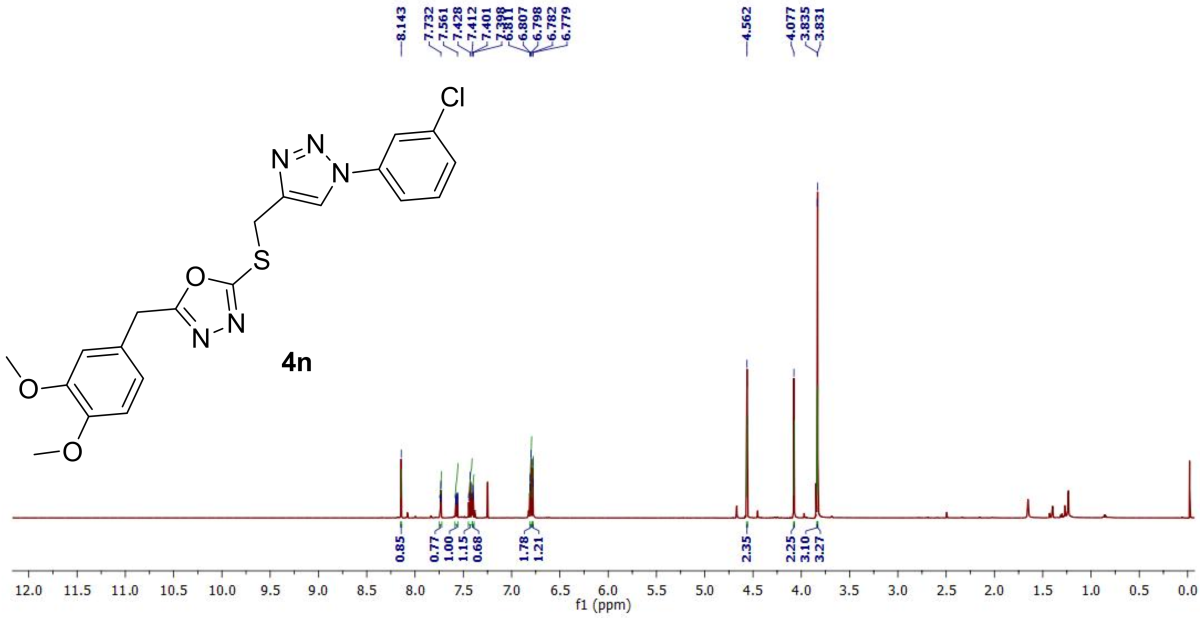

^{1}H NMR of **4n**

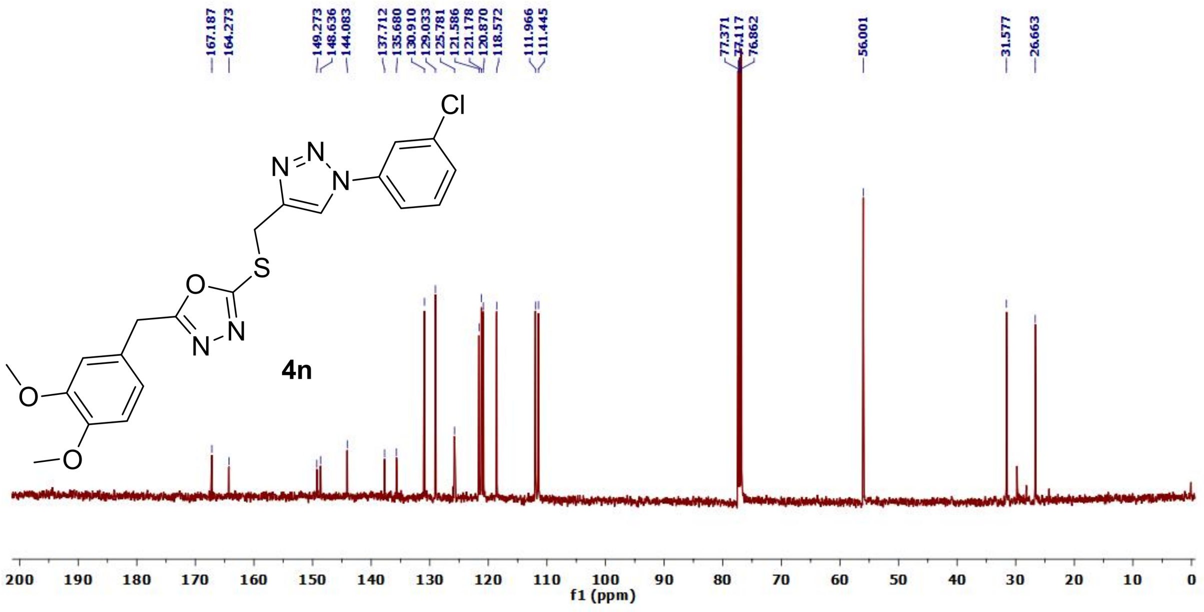

^{13}C NMR of **4n**

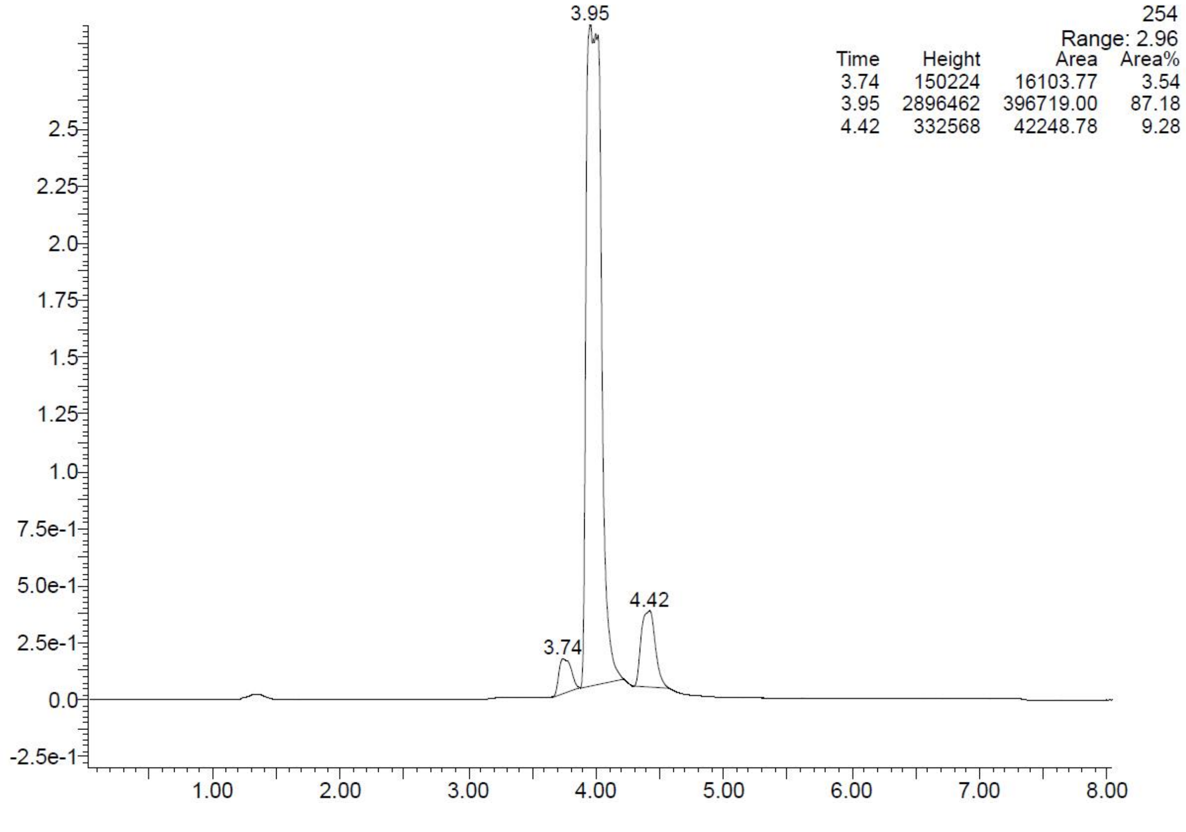

Liquid chromatogram of **4n**

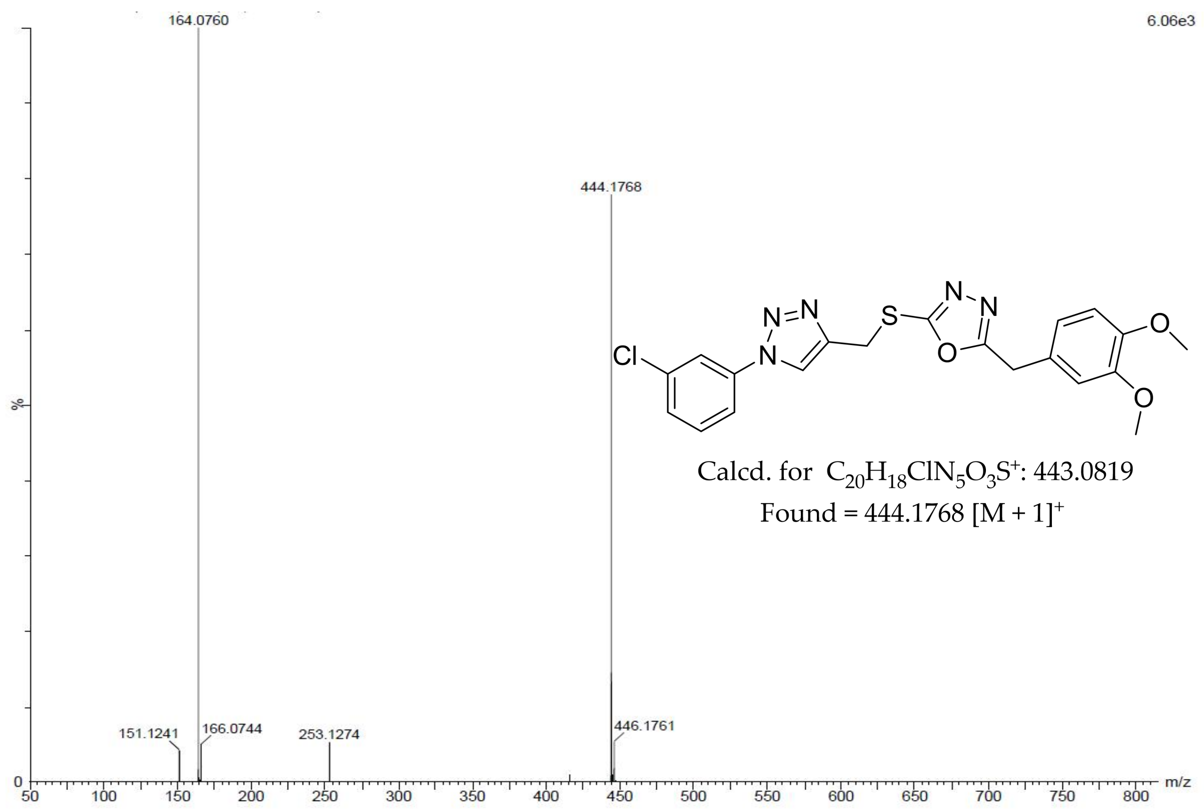

Mass spectra of **4n**

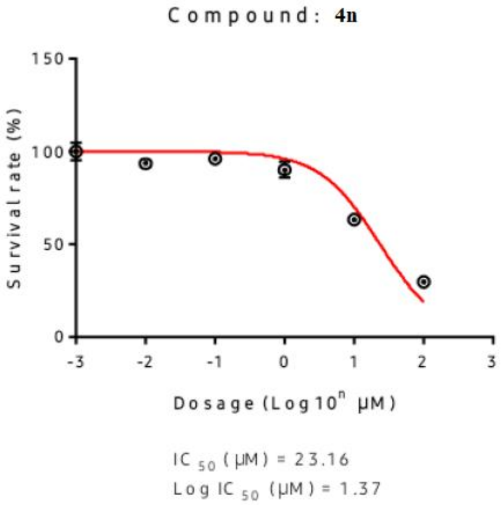

- Cell line: MCF-7 (2000 cells/per well 96-well plate)
- Treated time: 72hrs
- Assay: alamarBlue (4hrs incubated)
- Data: **4n**

Conc. (μM)	Viability	
	AVE.	± SD.
0	100.00	4.88
0.01	93.34	2.45
0.1	95.85	1.55
1	90.03	4.33
10	63.09	1.44
100	29.76	0.55

Cytotoxicity assay of **4n**

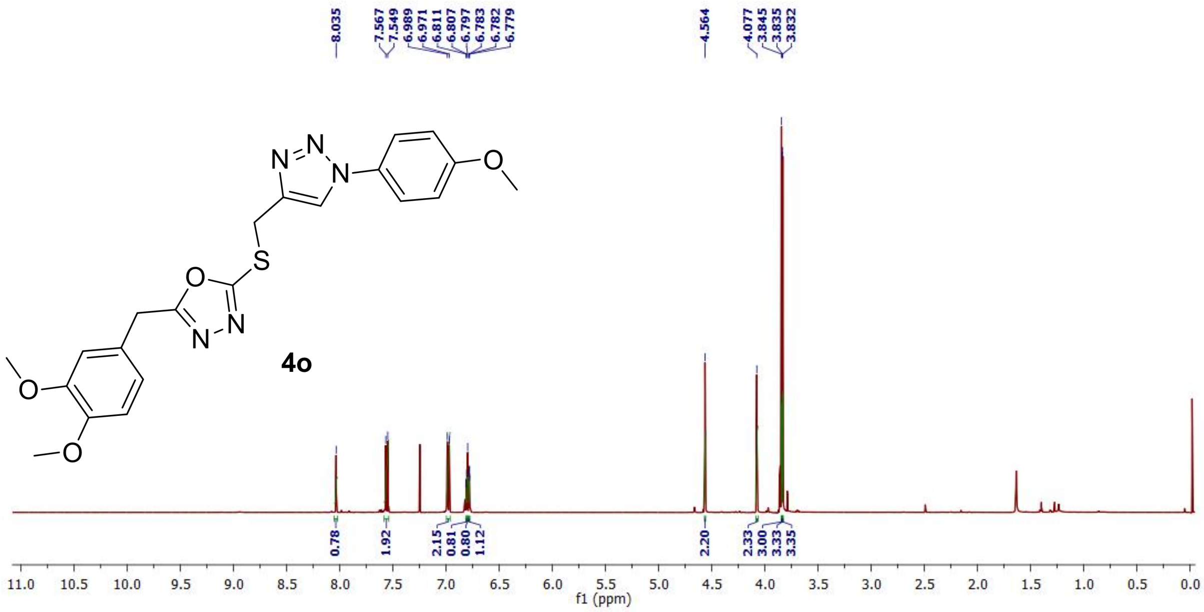

^{1}H NMR of **4o**

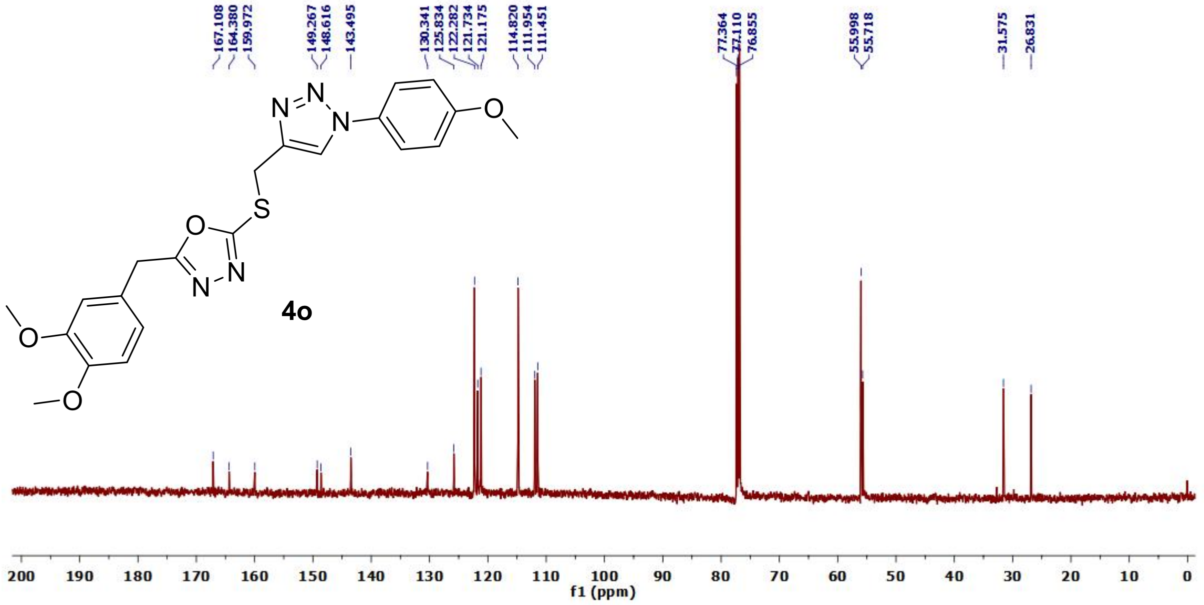

^{13}C NMR of **4o**

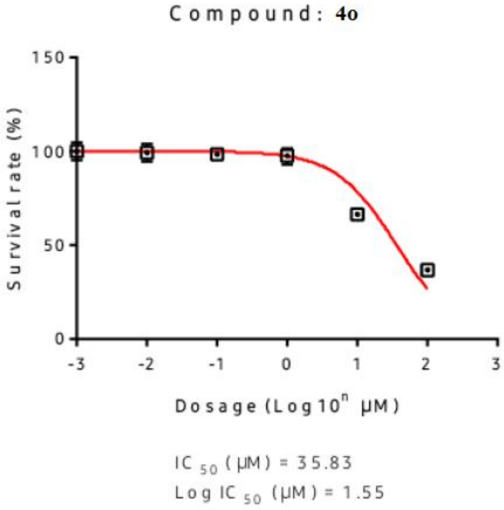

- Cell line: MCF-7 (2000 cells/per well 96-well plate)
- Treated time: 72hrs
- Assay: alamarBlue (4hrs incubated)
- Data: **4o**

Conc. (μM)	Viability	
	AVE.	± SD.
0	100.00	4.88
0.01	99.23	4.63
0.1	98.28	2.95
1	97.35	4.15
10	66.42	1.91
100	36.72	0.69

Cytotoxicity assay of **4o**

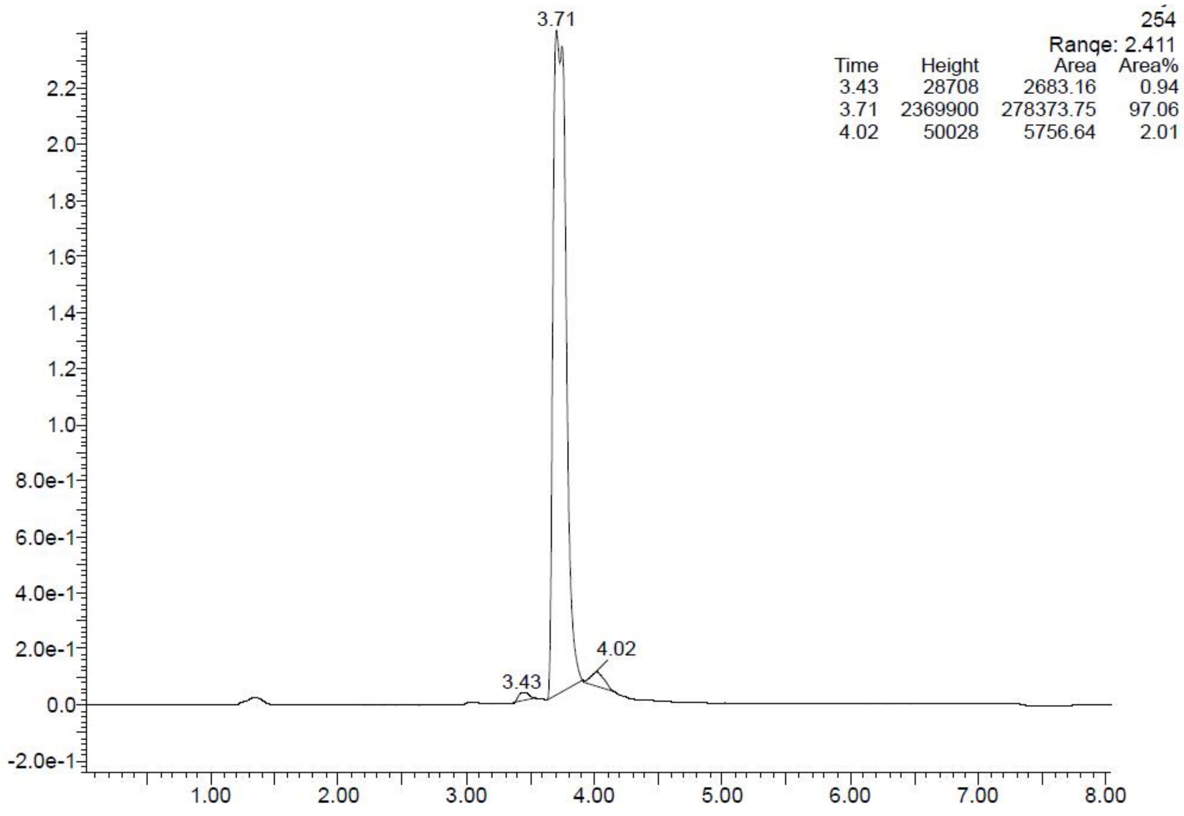

Liquid chromatogram of **4o**

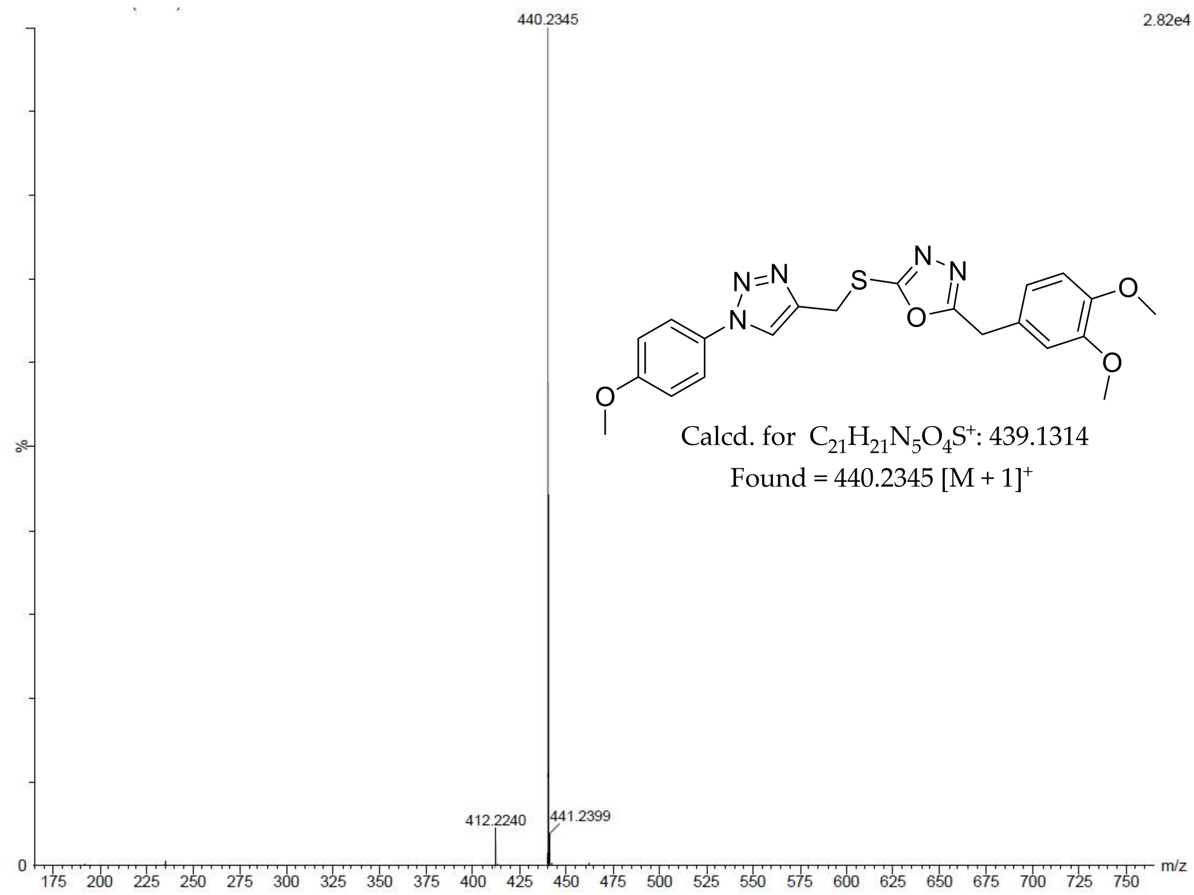

Mass spectra of **4o**

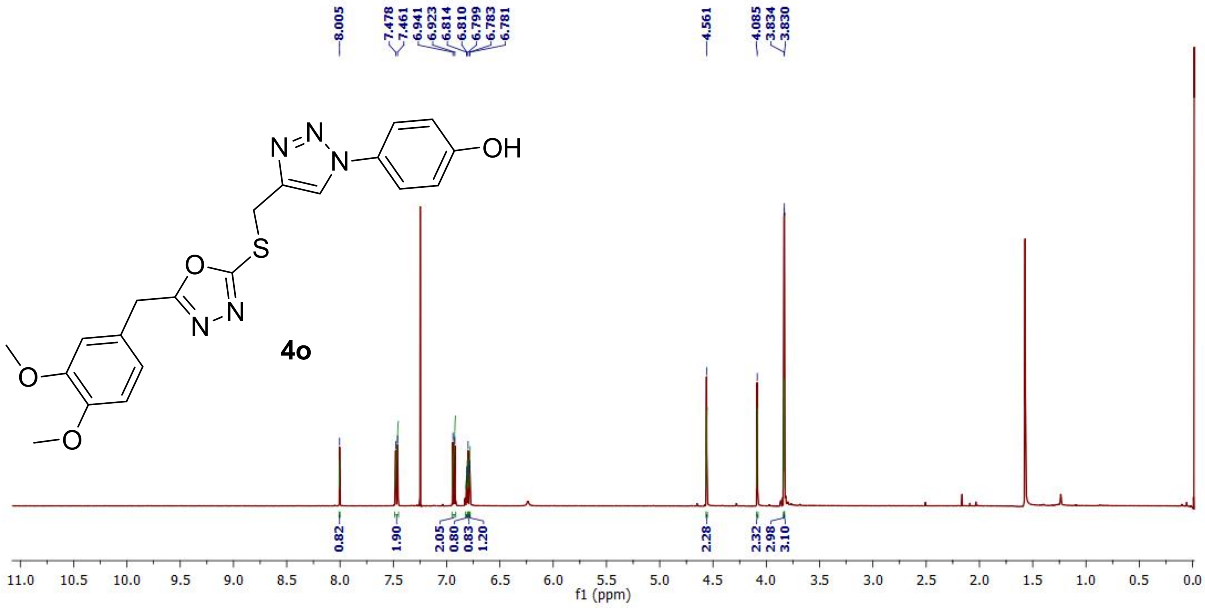

^{1}H NMR of **4p**

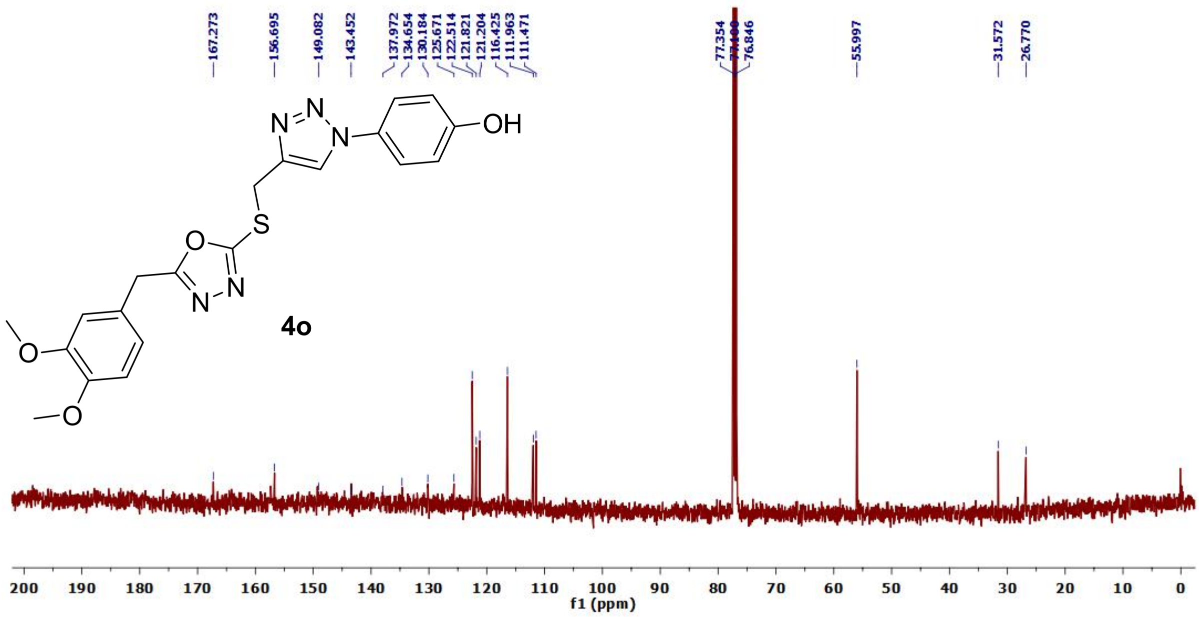

^{13}C NMR of **4p**

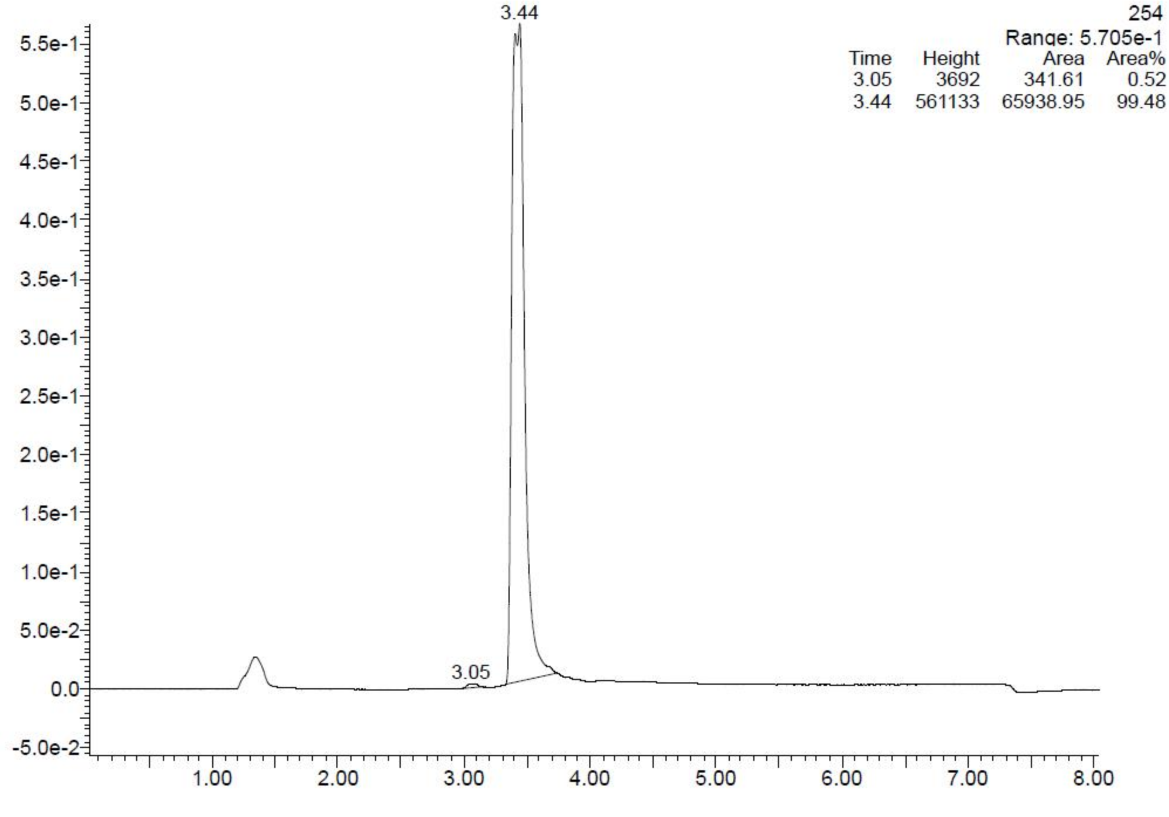

Liquid chromatogram of **4p**

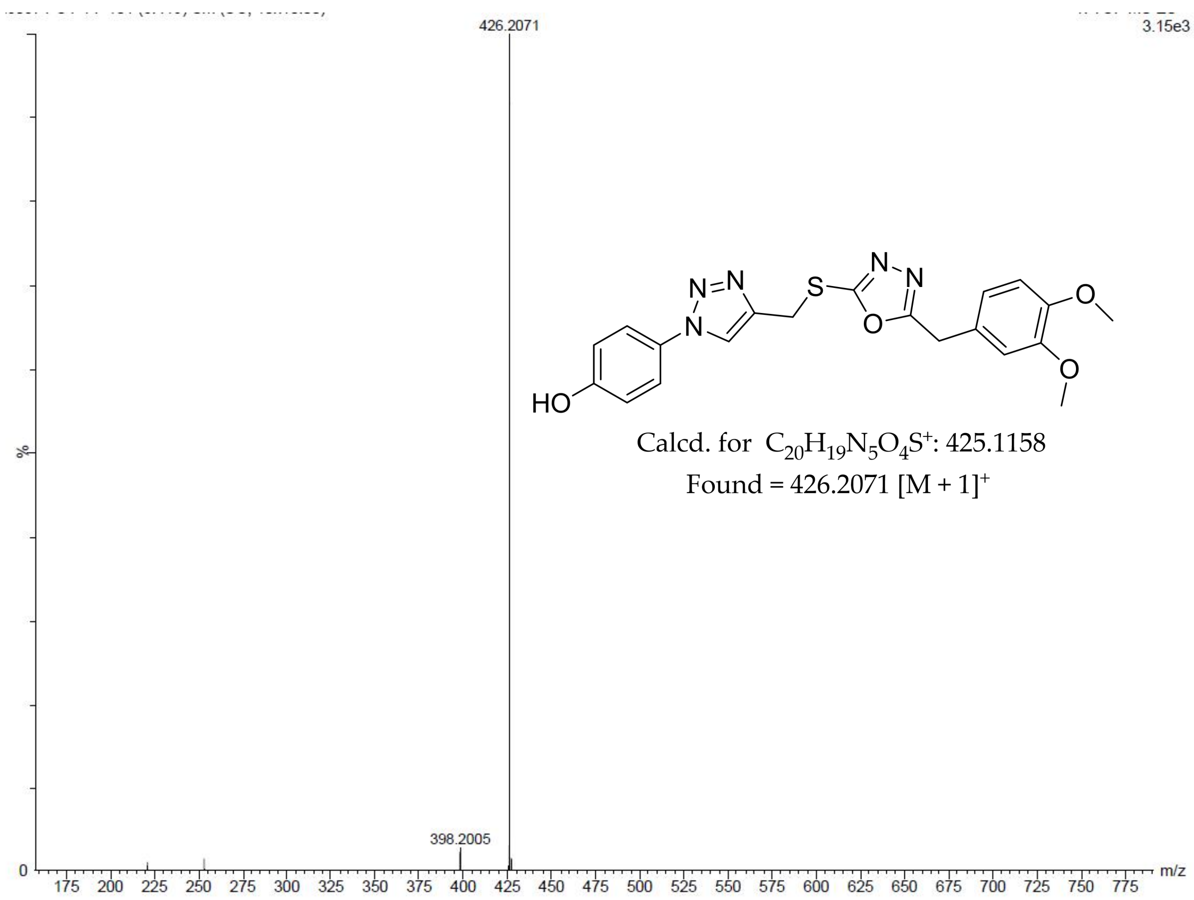

Mass spectra of **4p**

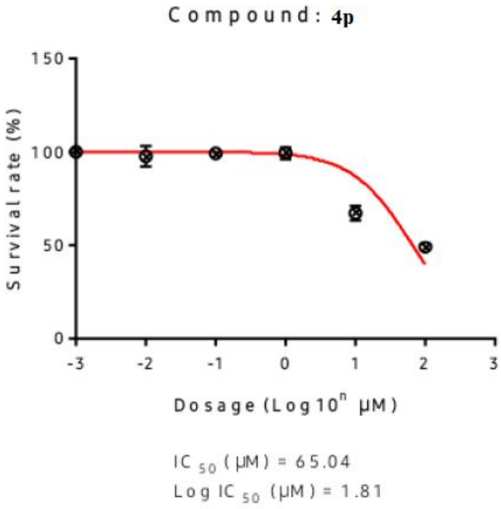

- Cell line: MCF-7 (2000 cells/per well[96-well plate])
- Treated time: 72hrs
- Assay: alamarBlue (4hrs incubated)
- Data: **4p**

Conc. (μM)	Viability	
	AVE.	± SD.
**0**	100.00	0.89
**0.01**	97.49	5.84
**0.1**	98.93	2.67
**1**	99.38	3.18
**10**	67.17	4.00
**100**	48.80	2.04

Cytotoxicity assay of **4p**

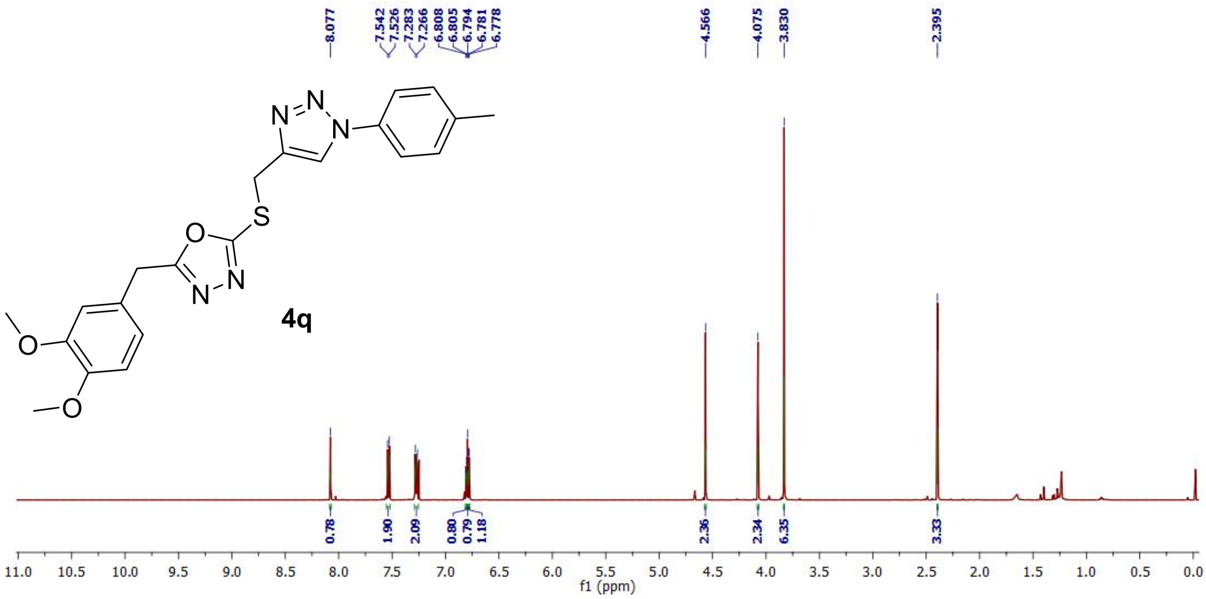

^{1}H NMR of **4q**

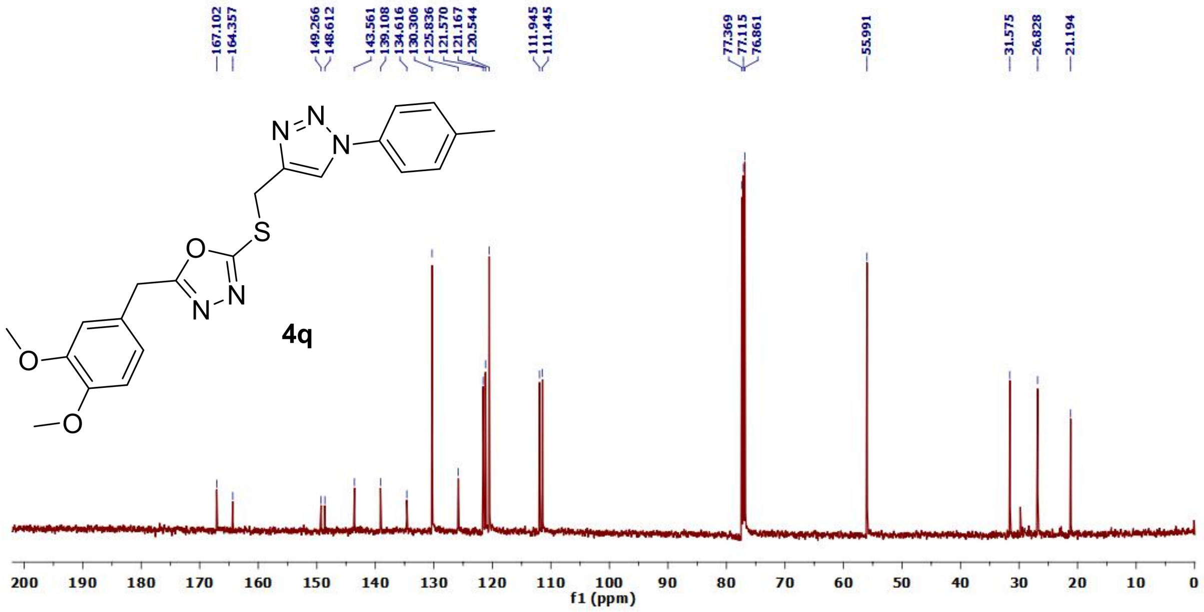

^{13}C NMR of **4q**

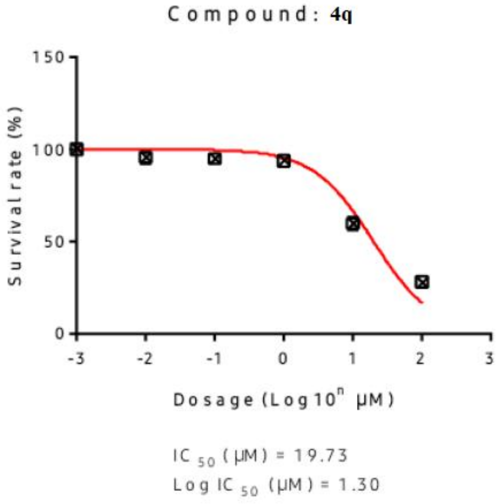

- Cell line: MCF-7 (2000 cells/per well[96-well plate])
- Treated time: 72hrs
- Assay: alamarBlue (4hrs incubated)
- Data: **4q**

Conc. (μM)	Viability	
	AVE.	± SD.
0	100.00	0.89
0.01	95.57	3.08
0.1	94.87	1.45
1	93.72	1.29
10	59.71	3.40
100	27.94	0.01

Cytotoxicity assay of **4q**

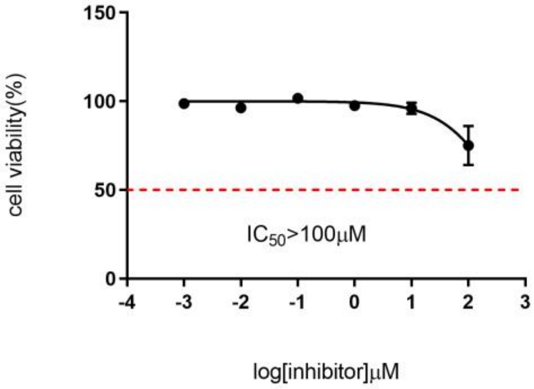

Conc.(μM)	Viability	
	AVE.	±SD
0	100.00	2.66
0.01	96.27	2.39
0.1	101.67	2.52
1	97.57	2.28
10	96.00	3.21
100	75.00	11.00

Cytotoxicity assay for the compound **4q (MCF-10A)**

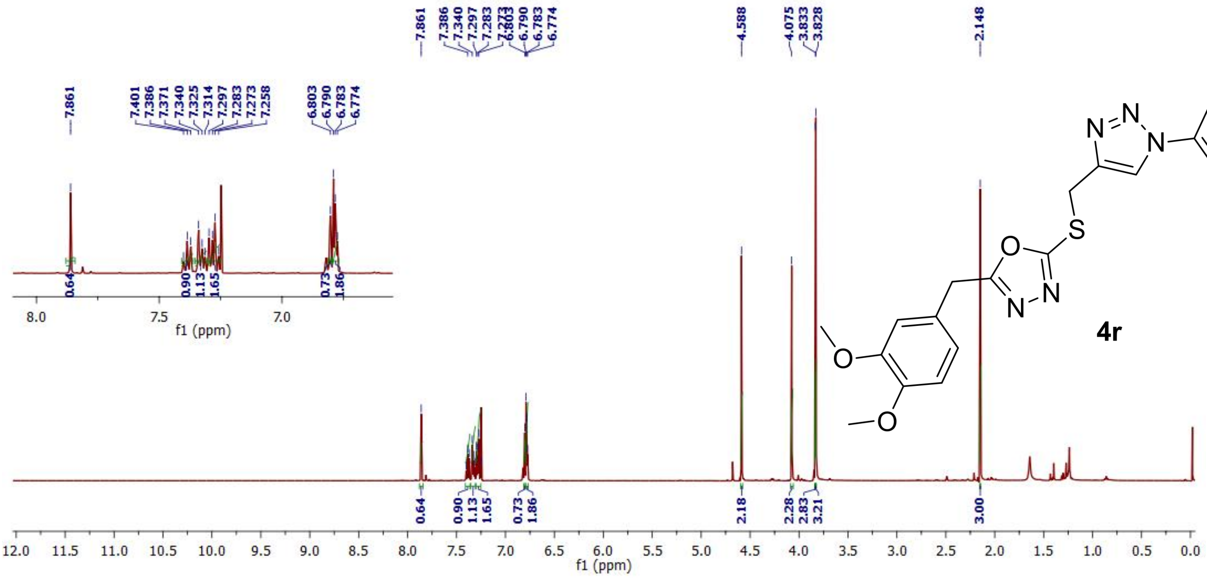

^{1}H NMR of **4r**

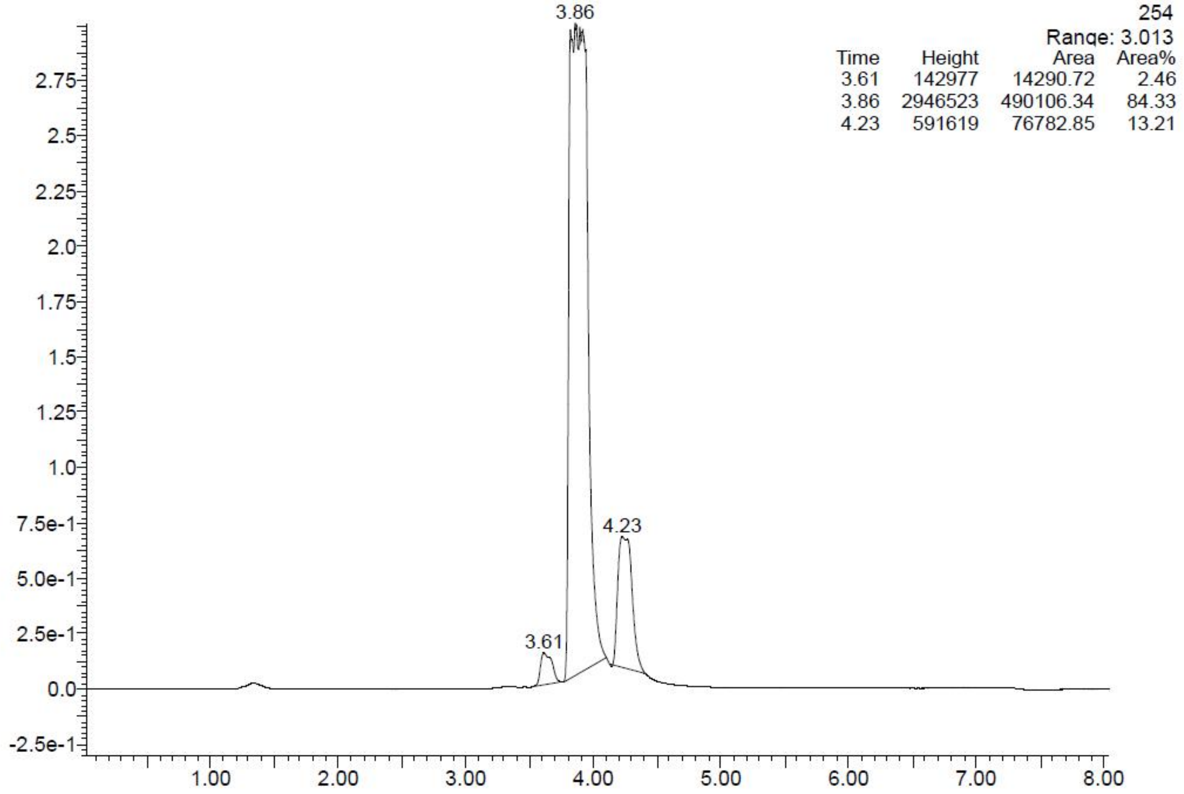

Liquid chromatogram of **4r**

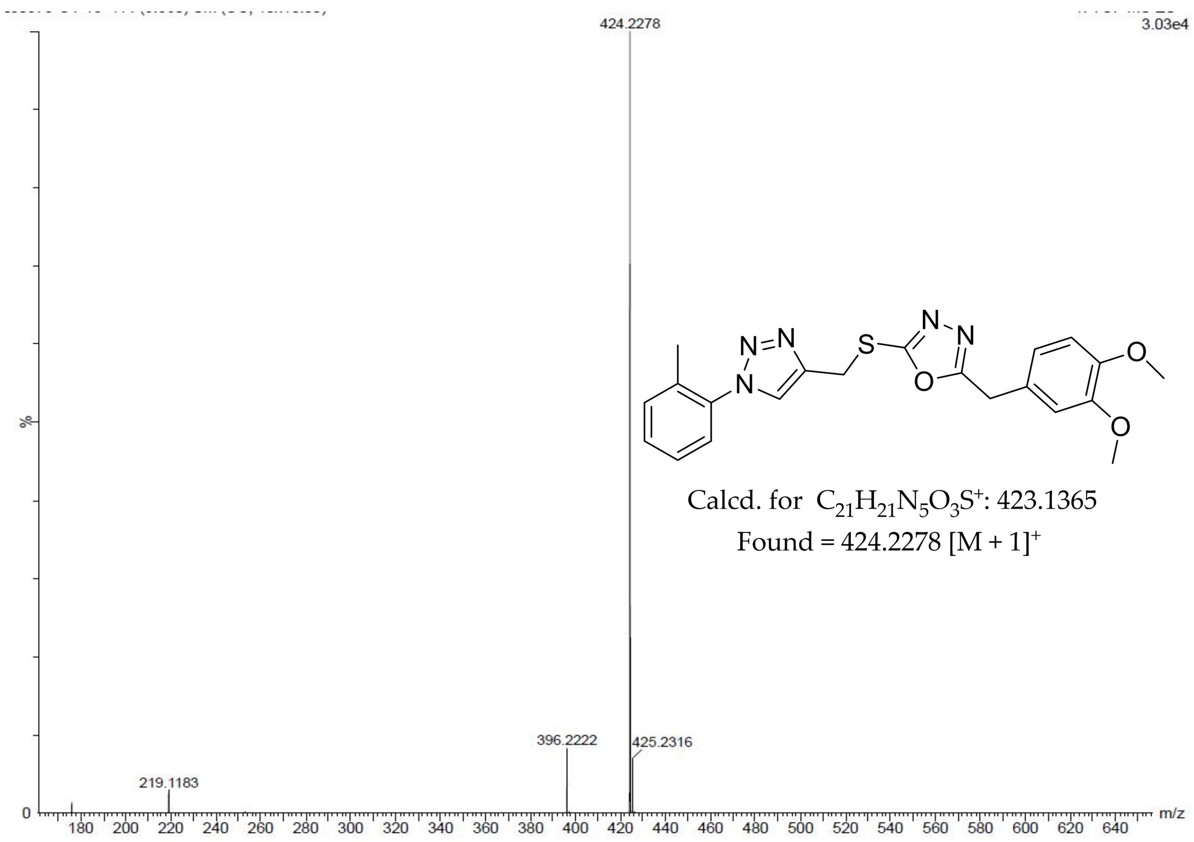

Mass spectra of **4r**

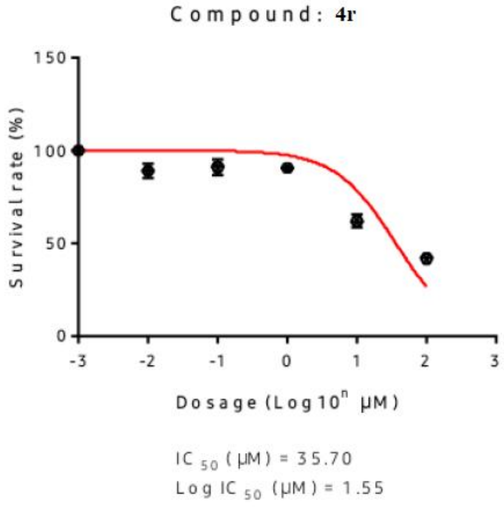

- Cell line: MCF-7 (2000 cells/per well[96-well plate])
- Treated time: 72hrs
- Assay: alamarBlue (4hrs incubated)
- Data: **4r**

Conc. (μM)	Viability	
	AVE.	± SD.
0	100.00	0.89
0.01	89.10	4.09
0.1	90.93	4.21
1	90.67	1.19
10	61.89	3.54
100	41.66	1.56

Cytotoxicity assay of **4r**
